# Regional drying over the Western U.S. driven by enhanced atmospheric subsidence amid global moistening from 1980 to 2020

**DOI:** 10.1038/s41467-026-71818-w

**Published:** 2026-04-16

**Authors:** Qinghua Ding, Tiffany Shaw, Hailan Wang, Ian Baxter, Jiang Zhu

**Affiliations:** 1 Department of Geography, and Earth Research Institute, University of California, Santa Barbara, Santa Barbara, CA USA; 2 Department of the Geophysical Science, The University of Chicago, Chicago, IL USA; 3 NOAA/Climate Prediction Center, College Park, MD USA; 4 NSF National Center for Atmospheric Research, Boulder, CO USA

**Keywords:** Hydrology, Hydrology, Atmospheric dynamics, Attribution

## Abstract

As the global climate has warmed anthropogenically over the past decades, the atmosphere across most of the globe has experienced significant moistening, except for a “moistening hole” (MH) -like change over the Western U.S. This regional anomaly since 1980 is at odds with the forced response of climate models to global warming in this region. Here, through analysis of a wide array of observations and water-tagging enabled simulations, we find that atmospheric forcing originating from the North Pacific contributes to the MH. A barotropic high-pressure circulation trend over the North Pacific, driven by observed sea surface temperature cooling in the tropical Eastern Pacific, enhances atmospheric sinking over the Western U.S. through equatorward cold air advection. This intensified atmospheric descent suppresses precipitation and weakens land-sourced evaporation, which are critical for replenishing atmospheric moisture in the region. We suggest that focusing on low-frequency changes of atmospheric vertical motion may offer insights into assessing and projecting climate stress and drought risks posed by long-term atmospheric moisture deficits in arid regions.

## Abstract (web summary)

This study shows that despite widespread atmospheric moistening under global warming, the Western U.S. has experienced a persistent multidecadal drying trend over the past four decades, sustained by atmospheric subsidence and reduced regional moisture recycling triggered by tropical eastern Pacific cooling.

## Introduction

Over recent decades, tropospheric atmospheric moisture has increased across most of the Northern Hemisphere (NH), driven by rising temperatures associated with global warming^1–3^. This heightened moisture content has significantly influenced many aspects of the global climate system, including atmospheric and oceanic conditions, the energy budget, the hydrological cycle, and the characteristics of weather and climate variability^4–7^. However, a notable exception to this widespread moistening trend is observed in the Western U.S., where atmospheric specific humidity has slightly declined since 1980^8,9^. This phenomenon, termed the “moistening hole” (hereafter referred to as MH) in this study, represents an iconic feature of the local hydrological cycle over the past few decades, contributing to prolonged droughts across the meteorological, agricultural, hydrological, and socioeconomic sectors of the region^10–13^.

The causes of this long-term atmospheric drying trend in the Western U.S., which contrasts with the broader NH moistening pattern, remain unclear^9^. Most coupled climate models, when forced by observed historical changes in anthropogenic forcing over the past decades, exhibit moistening trends nearly everywhere across the globe, especially in the NH, demonstrating a robust constraint of atmospheric temperature on moisture following the Clausius-Clapeyron relationship^14^. The absence of this expected moistening over the Western U.S. highlights the complex interplay of multiple processes within the local hydrological cycle in arid regions, such as vapor pressure deficit, precipitation, evaporation, soil moisture, atmospheric moisture transport, surface heat fluxes, and atmospheric instability and weather activity, which individually or collectively regulate the sensitivity of atmospheric moisture to temperature changes^15–25^. This discrepancy may also reflect the incomplete representation of physical processes or inaccurate parameterizations of key feedback mechanisms over dry climate zones in our climate models^9,26,27^, raising concerns about their ability to accurately project future regional moisture trends in arid areas. Alternatively, the observed MH may partly result from remote variations with internal or anthropogenic origins, a potential driver that has received sustained attention in recent research focused on this region^15,28,29^.

Remote variations, particularly those linked to low-frequency tropical and extratropical sea surface temperature (SST) variability in the Pacific and Atlantic, can generate large-scale circulation patterns that influence storm activity, and moisture recycling and transport in North America, thereby contributing to long-term humidity anomalies^30–37^. For instance, during La Niña years or negative phases of the Pacific Decadal Oscillation (PDO), the Western U.S. often experiences extended periods of dryness or clusters of drought years due to shifts in large scale circulation patterns and reduced moisture transport from the Pacific^38–42^. Other factors, such as Arctic sea ice loss, have also been proposed as potential contributors to drought in the region^43^. However, it remains uncertain whether the recent atmospheric drying trend in the U.S. West can be attributed to similar processes and, if so, what mechanisms underlie these processes.

Recent advances in modeling techniques, particularly the development of water-tagging and nudging methods in CESM1, have provided powerful tools for investigating the sources and pathways of atmospheric moisture^44–47^. These methods represent a significant improvement over traditional techniques, such as diagnostic moisture budgets^48^, regional water tagging^49^, and offline water tracking methods^50^, by enabling precise tracing of moisture from its origin to its destination under observed wind and temperature conditions on a global scale. This capability is instrumental in identifying key global drivers of regional moisture variability and the processes influencing local moisture recycling.

In this study, we leverage these advanced tools, along with analyses of reanalysis data, large ensemble simulations, and AMIP experiments (Methods), to investigate the potential roles of different climate forcings in the formation of the MH. This approach allows us to disentangle the complex interactions between anthropogenic forcing and internal variability that result in regional deviations in atmospheric specific humidity over the U.S. West, contrasting with the widespread global warming-induced moistening trend. Our findings provide insights into the mechanisms governing interactions among the atmosphere, ocean, and land that contribute to regional moisture trends, both historically and in the future. The MH as referred to in this study, was previously discussed for the period 1980–2020^9^. Building on this research^9^, our study also uses a diverse array of observational and model datasets within the same timeframe (Methods). In particular, we primarily use annual mean data to investigate long-term trends in the following analysis, as atmosphere-ocean–land interactions typically possess complex cross-seasonal variations that warrant further analysis (Methods).

In the following analysis, we primarily present results based on ERA5, which exhibits a stronger MH trend than other reanalyzes (Methods). It remains unclear whether this difference reflects inhomogeneities in the original observations or a real physical signal. Because ERA5 fields are also used in our nudging simulations, any possible bias in ERA5 may be transmitted into the nudging experiments. Our analysis should be viewed as a qualitative examination of the formation of the MH rather than a strictly quantitative assessment.

## Results

### Observed large-scale climate trends over North America

**Fig. 1:**
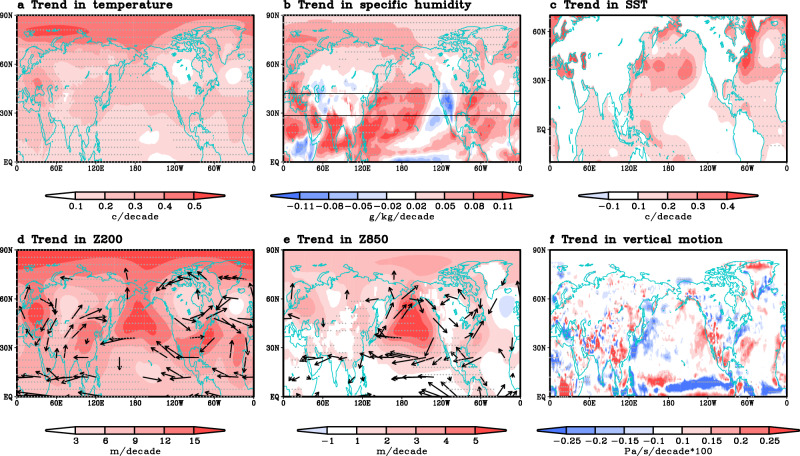
Observed annual mean trends of multiple large-scale fields in the Northern Hemisphere from 1980 to 2020. Linear trends of annual mean: **a** tropospheric temperature (averaged between 200–1000 hPa, °C/decade), **b** lower tropospheric specific humidity (averaged between 650–1000 hPa, g/kg/decade), **c** sea surface temperature (SST, °C/decade), **d** 200-hPa geopotential height (Z200, m/decade) and wind vectors (showing only statistically significant trends at coarse resolution), **e** 850-hPa geopotential height (Z850, m/decade) and wind vectors (showing only statistically significant trends at coarse resolution), and **f** tropospheric vertical motion (averaged between 300–800 hPa, scaled by 100 times, Pa/s/decade × 100; positive shading indicates downward motion) from ERA5 (ERSST5 for SST) during 1980–2020. Trends significant at the 95% confidence level are marked with dots. Regions used in subsequent zonal-vertical cross-section analyses are highlighted in (**b**).

**Fig. 2:**
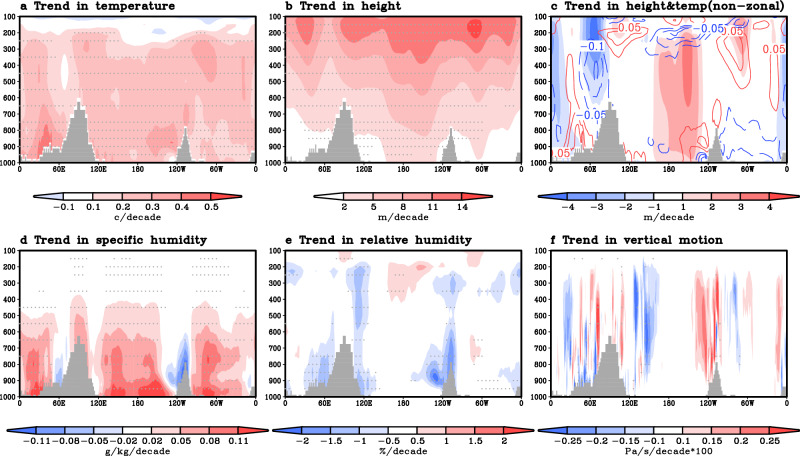
Observed annual mean trends of multiple large-scale fields along a vertical transect (28-42˚N) from 1980 to 2020. Linear trends of meridional (averaged between 28°–42°N) annual mean tropospheric variables: **a** temperature (°C/decade), **b** geopotential height (m/decade), **c** non-zonal components of geopotential height (shading, m/decade) and temperature (contours, °C/decade), **d** specific humidity (g/kg/decade), **e** relative humidity (%/decade), and **f** vertical motion (shading, scaled by 100 times, Pa/s/decade × 100), derived from ERA5 during 1980–2020. Trends significant at the 95% confidence level are marked with dots.

Over the past four decades, the NH annual mean atmospheric temperature in the troposphere (200–1000 hPa) has increased by 0.16 °C/decade (Fig. 1a). The warming is most significant in the Arctic (poleward of 60°N) and moderate in the subtropics (30–45°N). Along with this widespread atmospheric warming, the NH annual mean specific humidity in the lower troposphere has risen by ~1%/decade relative to the 1980–1989 average across most regions (Fig. 1b). However, a notable exception is observed over the Western U.S., where specific humidity has declined significantly by approximately −2%/decade (relative to the 1980-1989 average), particularly in the lower troposphere (650 hPa to the surface) (Fig. 2d). This drying trend in the lower troposphere is the most pronounced across the NH extratropics, both in magnitude and spatial extent. We also examine in-situ radiosonde-measured vapor pressure over North America and find that the MH is clearly manifested in the Western U.S. (Methods, Supplementary Fig. 1 and 2). ERA5 captures this observed MH pattern well, particularly in the lower troposphere, while MERRA2 shows significant moistening across the continent and slight drying over the Western U.S. Accordingly, the following analysis focuses on this lower tropospheric layer (650–1000 hPa), where the MH feature is most striking (Fig. 2d) and consistently seen in both ERA5 and in-situ observations (Methods).

**Fig. 3:**
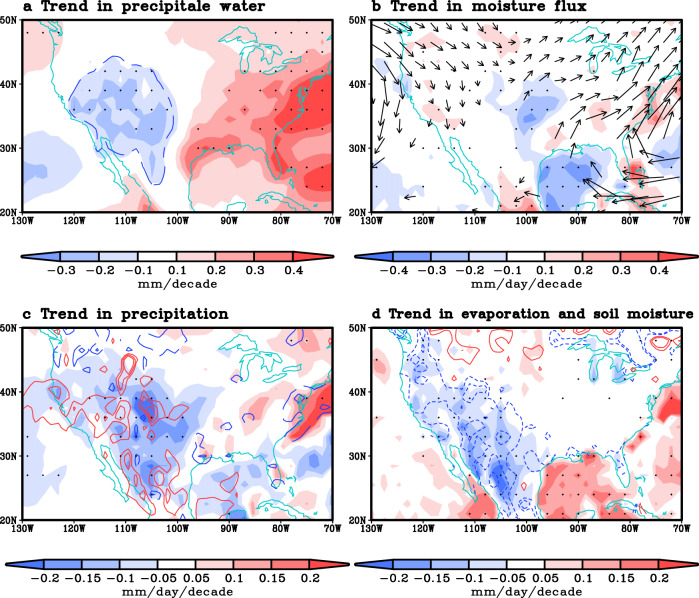
Observed annual mean trends of multiple hydrological fields in and around the U. S. from 1980 to 2020. Linear trends of annual mean: **a** precipitable water (averaged between 650–1000 hPa, mm/decade), **b** Integrated Vapor Transport (IVT) and its divergence (IVT was derived from hourly data^79^, mm/day/decade, positive shading indicates a convergence of atmospheric moisture), **c** precipitation (mm/day/decade, shading), and d) evaporation (mm/day/decade, shading) from ERA5 during 1980–2020. Contours in (**c**) show tropospheric vertical motion trends (averaged between 300–800 hPa), where positive values indicate downward motion. Contours in (**d**) show annual mean soil moisture trends (mm/day/decade, CPC soil moisture). Significant trends (shown as shading in each panel) above the 95% confidence level are marked with dots. In (**a**), the blue dashed contour indicates the moistening hole region, where the drying trend of specific humidity (or precipitable water) averaged between 650–1000 hPa is strongest over the period.

This atmospheric drying over the West occurs throughout the year, with the most significant declines observed in spring and summer (Methods). During the same period, circulation changes, illustrated by linear trends in annual mean geopotential height at 200 hPa (Z200; Fig. 1d) and geopotential height at 850 hPa (Z850; Fig. 1e), show barotropic high-pressure trends over the subtropical Pacific and Atlantic, with moderate rises over North America. To the east of the strengthened subtropical North Pacific high-pressure system, enhanced northwesterly flow and subsidence (Fig. 1f) are evident, likely inhibiting moisture intrusion into the Western U.S. and suppressing local precipitation (Fig. 3c). This circulation pattern resembles the Pacific-North American (PNA) pattern, which is often linked to ENSO- or PDO-related SST anomalies in the tropical and North Pacific^51^. The non-zonal component of the Z200 trend and the associated wave-activity flux reveal a clear Rossby wave train emanating from the tropical Eastern Pacific, suggesting a tropical origin for the anomalous high-pressure trend over the North Pacific (Supplementary Fig. 3).

Over the past four decades, little to no SST warming trends are observed in the tropical Eastern Pacific and surrounding regions (Fig. 1c), in contrast to significant SST warming of 0.1–0.3 °C per decade across most other regions. An apparent “no SST trend” in the tropical Eastern Pacific may result from a slight cooling trend superimposed on the global warming signal in the tropics. This SST cooling may reflect either a negative phase of the PDO^36^ or an increased frequency of La Niña events since 2000^52^.

To further illustrate the vertical structure of these long-term circulation trends, a zonal-vertical cross-section averaged over 28.5°–42°N (encompassing the Western U.S.) is presented in Fig. 2. Zonally uniform warming and height rises dominate much of the atmosphere along these latitudes, particularly from the dateline to the U.S. West Coast (Fig. 2a, b). Removing the zonal mean component, which primarily reflects global warming responses, reveals a distinct regional high-pressure anomaly over the subtropical North Pacific (Fig. 2c). To the east of this anomaly, stronger subsidence (Fig. 2f), warming (Fig. 2a), and drying in both specific (Fig. 2d) and relative humidity (Fig. 2e) are observed in the lower troposphere.

Diagnostic analysis using the traditional omega equation (Methods) reveals that these anomalous circulation changes (Fig. 1d&e) over the Western U.S. induce long-term sinking motion, primarily through enhanced cold air advection (${V}_{g}\cdot {\nabla }_{p}T$, term 2 in Eq. 2), rather than long-term changes in the vertical differential advection of geostrophic absolute vorticity (term 1 in Eq. 2, Supplementary Fig. 4). This sinking motion may further hinder precipitation, inducing diabatic cooling that, in turn, reinforces the atmospheric sinking (Supplementary Fig. 5c and f). The spatial alignment of trends in cold air advection (Supplementary Fig. 5a and b) and vertical motion in the horizontal spatial pattern, as well as along this latitude band (Supplementary Fig. 5d and e), highlights the importance of this mechanism, justifying the use of the omega equation in this analysis. Thus, the formation of the MH pattern, as shown across multiple variables in Fig. 2, may be speculated to arise from a trend of anomalous northwesterly winds east of the barotropic high-pressure anomaly. Once a barotropic high-pressure anomaly builds up over the North Pacific, the anomalous northwesterly flow to its east generates stronger cold air advection over the Western U.S. To offset the induced cooling, large-scale sinking motion is established there, leading to adiabatic warming.

During 1980-2020, precipitable water, evaporation, and precipitation all decrease over the West (Fig. 3). While reduced lower-tropospheric relative humidity (Fig. 2e) might favor stronger evaporation, the decline in precipitation appears to play a more dominant role by limiting water supply, as evidenced by the decreasing soil moisture trend over the West (Fig. 3d).

To understand the processes driving atmospheric drying, a moisture budget analysis is performed (Fig. 3), focusing on the contributions of Integrated Vapor Transport (IVT) divergence, precipitation, and evaporation. Annual mean IVT shows weak trends over the past four decades, with no significant change in its divergence. In contrast, both precipitation and evaporation exhibit declining trends, contributing to an increasingly arid hydroclimate. Although uncertainties in reanalysis data and strong cancellation among these three processes prevent us from closing the moisture budget over the Western U.S., reduced evaporation seems to play a more direct role in atmospheric drying, as its long-term decrease aligns more closely with the decline of atmospheric moisture.

To investigate the role of vertical motion in mediating precipitation and evaporation over North America, grid-to-grid correlations between the three variables are calculated (Supplementary Fig. 6). These analyses reveal that, to first order, downward motion is associated with reduced precipitation, which in turn limits evaporation. Although these grid-to-grid correlation maps primarily reflect connections on interannual timescales, the same mechanisms may also operate on long-term trends. The stronger sinking motion trend observed in the U.S. West over the past four decades likely contributes to suppressed precipitation and atmospheric drying by reducing local moisture recycling (Fig. 3). This chain of processes, inferred from a series of statistical analyses based on ERA5, remains speculative at this stage and requires further validation through multiple CESM2 large ensemble experiments, AMIP runs, and water-tagging simulations, as discussed in the following sections.

### Simulated large-scale climate trends over North America

**Fig. 4:**
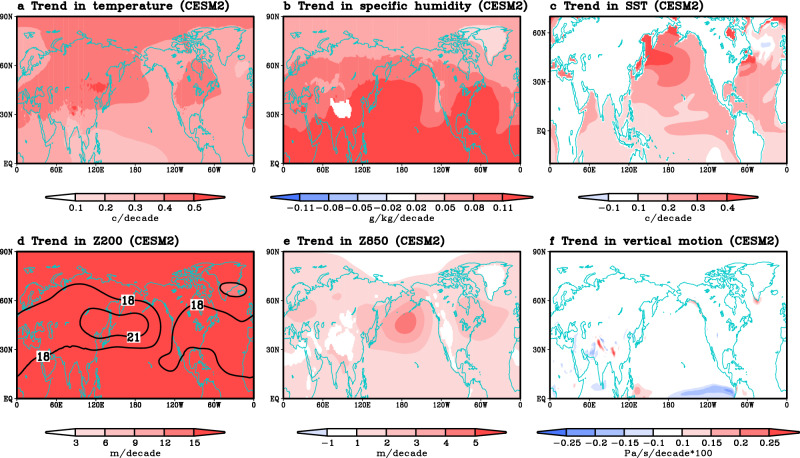
Simulated annual mean trends of multiple large-scale fields in the Northern Hemisphere by CESM2-LE from 1980 to 2020. Linear trends of annual mean: **a** tropospheric temperature (averaged between 200–1000 hPa, °C/decade), **b** lower tropospheric specific humidity (averaged between 650–1000 hPa, g/kg/decade), **c** sea surface temperature (SST, °C/decade), **d** 200-hPa geopotential height (Z200, m/decade), (**e**) 850-hPa geopotential height (Z850, m/decade), and f) tropospheric vertical motion (averaged between 300–800 hPa, scaled by 100 times, Pa/s/decade × 100; positive shading indicates downward motion) from the ensemble mean of 100 members of CESM2-LE during 1980–2020. The color scale is adapted from that of Fig. 1 for easy comparison. In (**d**), trends in Z200 exceeding the maximum value of the color scale are shown in contours. From (**a**–**e**), all values are significant at the 95% confidence level. In (**f**), no values within the entire domain are significant at the 95% confidence level.

To understand the forced climate response to global warming over the four-decade period, we examined two ensembles forced by anthropogenic forcing from 1980 to 2020: one consisting of 27 CMIP6 models (Supplementary Fig. 7) and the other using CESM2-LE with 100 members (Fig. 4). Both ensembles show remarkably consistent results across key variables, including increased warming, higher specific humidity, and no significant changes in relative humidity across the U.S. Additionally, both evaporation and precipitation exhibit slight increasing trends nationwide in CESM2-LE (Supplementary Fig. 8). Notably, the hydrological response in the West, as simulated by the ensemble means, differs substantially from the observed MH pattern. Instead, both ensembles reveal increases in atmospheric moisture, following global trends. This suggests that the Western U.S. does not exhibit distinct behavior compared to other regions in its response to global warming when considering only the effects of anthropogenic forcing in models.

In the zonal-vertical plots (Supplementary Figs. 9 and10), the simulated circulation trend patterns in the ensemble mean show substantial differences from observations, exhibiting more zonally uniform warming and widespread upward motion trends, particularly over the continents, which stand in sharp contrast with the observed trends of high-pressure anomalies over the North Pacific and associated sinking motion over the Western U.S. After removing the zonal mean component from the height trend patterns (Supplementary Figs. 9c and 10c), a weak high-pressure system emerges around the dateline with associated sinking motion to its east. However, this high-pressure system is too weak and shifted too far west compared to observations. Consequently, the vertical motion trend over the Western U.S. becomes upward. These ensemble-mean responses suggest that the observed sinking motion trend over the Western U.S. does not line up well with the model’s mean response to global warming. This is understandable, as global warming tends to favor stronger land warming, which may thermodynamically drive a planetary-scale sea breeze-like response in the lower troposphere^53^. Such a response enhances upward motion over land areas, such as the Western U.S., and downward motion over adjacent oceans (Supplementary Figs. 9f and 10f).

We also note that the observed trends in global SST and circulation patterns are poorly captured by the models (Fig. 4c and Supplementary Fig. 7c), a discrepancy highlighted in numerous previous studies^52,54,55^. The simulated SST trend resembles an El Niño-like warming pattern rather than the observed minimal warming or La Niña-like cooling in the tropical Eastern Pacific. This discrepancy has been attributed to several factors, including potential contributions from internal variability in the tropical and North Pacific^56^, anthropogenic aerosol forcing^54^, which may not be adequately represented in current large ensemble simulations, and model biases in simulating complex tropical air-sea interactions^55^ and tropical-extratropical interactions^57^. It remains an open question whether the observed lack of warming trend in the tropical Pacific is primarily anthropogenically or internally driven^52^. Further research is needed to improve our understanding of its formation.

### Composite analysis of simulated long-term trends in CESM2-LE

**Fig. 5:**
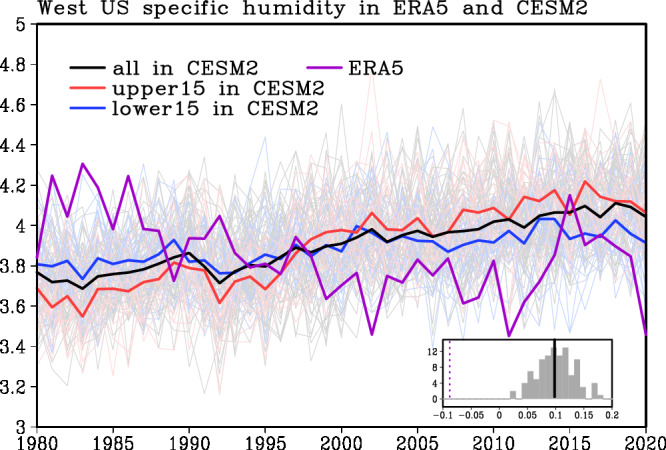
A wide range of simulated specific humidity trends over the US west in the CESM2-LE. Annual mean lower-tropospheric (averaged between 650–1000 hPa) specific humidity, averaged over the Western U.S. (outlined by the blue dashed contour in Fig. 3a, corresponding to the strongest moistening hole (MH) feature), from 1980 to 2020, derived from ERA5 (purple) and the CESM2-LE ensemble mean (black). Among the 100 members (gray), 15 exhibit the slowest moistening (lower-15 group, with blue for the group mean and thin blue lines for individual members), while 15 exhibit the fastest moistening (upper-15 group, with red for the group mean and thin red lines for individual members). The inset plot shows the probability density function of the specific humidity trend (as described above) over the MH region derived from CESM2-LE, with the ensemble mean indicated by the solid black vertical line and the ERA5 value indicated by the red dashed line.

Although most members of CESM2-LE fall short of capturing the observed MH pattern over the Western U.S. (Fig. 4b and 5), a subset of ensemble members performs slightly better in reflecting this long-term trend. This offers an opportunity to explore why this subgroup outperforms the rest. As all ensemble members share the same physical framework, their spread primarily reflects internal variability generated by the model system. In contrast, the CMIP6 ensemble exhibits a more complex scenario, where the spread is influenced not only by internal variability but also by differences in the sensitivity of physical schemes to the climate forcing specified by the CMIP6 experimental protocol. Therefore, our analysis in this section focuses on the CESM2-LE members to examine the role of internal variability in shaping the observed MH.

To identify the large-scale circulation characteristics related to the MH, we applied a composite analysis by comparing the slowest-moistening members to the fastest-moistening members in CESM2-LE. The slowest-moistening members (15 with the slowest moistening trends) exhibit the slowest increase in atmospheric specific humidity trends over the Western U.S., consistent to some degree with the observed changes of the MH. In contrast, the 15 fastest-moistening members significantly deviate from the observed drying patterns. Notably, none of the ensemble members exhibit a declining trend as observed, highlighting a limitation of the model in fully reproducing observed MH changes, even when internal variability is accounted for.

**Fig. 6:**
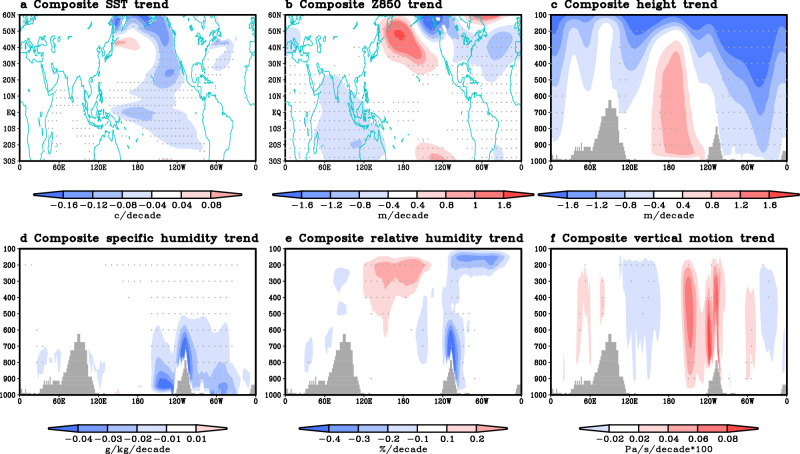
Role of internal variability in producing the observed moistening hole (MH) feature identified in CESM2-LE. The difference in composite linear annual mean trends between the lower-15 and upper-15 members of CESM2-LE (defined in Fig. 5) for: **a** sea surface temperature (SST), **b** 850-hPa geopotential height (Z850), and various meridional mean (averaged between 28°–42°) fields: **c** height, **d** specific humidity, **e** relative humidity, and **f** vertical motion. Differences between the two groups that are significant at the 95% confidence level are indicated by dots, based on a two-sample t-test.

As both groups are subject to the same external forcing, their differences highlight the contribution of internal variability. The differences between the two groups (Fig. 6 and Supplementary Fig. 11) reveal a decline in precipitable water over the Western U.S., accompanied by circulation and SST trend patterns that closely resemble observations over the North Pacific. Vertical-zonal plots show a trend of anomalous high pressure over the North Pacific and stronger sinking motions over the Western U.S., similar to observed changes (Fig. 2c, non-zonal component). Tropical Pacific SST cooling, which is not predetermined in our composite selection criteria, is manifested in the slow-minus-fast composite, suggesting it plays a crucial role in driving the drying trend in atmospheric specific humidity over the Western U.S. Further analysis of fields such as precipitation and evaporation show a closer match to observations compared to the ensemble mean. This analysis highlights the role of internally generated large-scale atmospheric descent over the Western U.S., linked to tropical Pacific SST cooling in the model, in creating favorable conditions for the MH.

To examine the process in reverse, we repeat the composite analysis to examine whether tropical Pacific SST cooling (relative to the ensemble-mean trend) can induce drying over the Western U.S. We select 15 members with the strongest SST cooling and 15 with the strongest warming trends over the tropical Eastern Pacific. The cooling-minus-warming composite shows a distinct wave train structure, leading to strong sinking motion and atmospheric drying in the Western U.S. (Supplementary Fig. 12). These results resemble several observed features, as well as those shown in Supplementary Fig. 11, reinforcing the role of tropical SST in shaping the MH pattern.

**Fig. 7:**
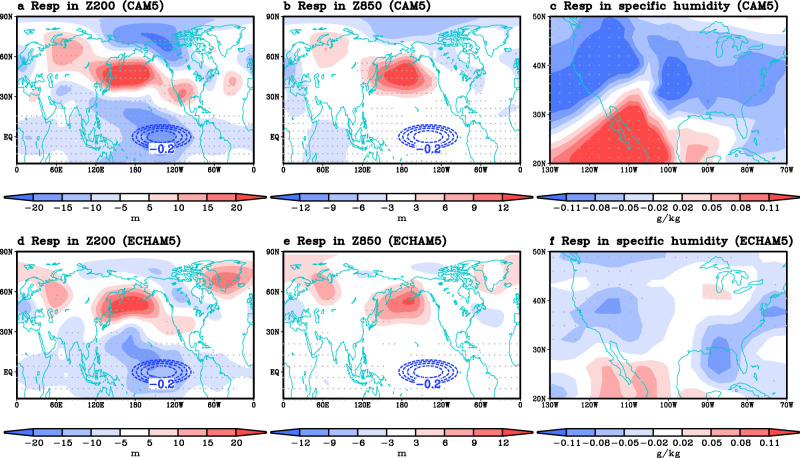
Two AGCM responses to a prescribed sea surface temperature (SST) cooling over the tropical eastern Pacific. Differences between the 30-year averages of annual-mean (**a**, **d**) 200-hPa geopotential height (Z200), (**b**, **e**) 850-hPa geopotential height (Z850), and (**c**, **f**) lower-tropospheric specific humidity averaged between 650 and 1000 hPa for the sensitivity run relative to the control run, shown for CAM5 (upper panels) and ECHAM5 (lower panels). The prescribed SST cooling anomaly in the sensitivity run relative to the control run (contour interval: −0.2 °C) is shown by blue dashed contours. Regions where the differences between the two runs are significant at the 95% confidence level are indicated by dots, based on a two-sample t test.

To more directly validate the role of boundary SST forcing in shaping the recent MH over the U.S. West, we examine the atmospheric response to a prescribed SST cooling anomaly using control and sensitivity runs from two AGCMs (Fig. 7). All model configurations (for example, global SST and sea ice, anthropogenic forcing) are identical between the control and sensitivity run, except that the latter includes an additional SST-cooling pattern imposed in the tropical Eastern Pacific (Methods). The difference between the two runs over the 30-year integrations highlights, in a qualitative sense, the important role of tropical Pacific cooling in driving an anomalous barotropic high over the North Pacific, which favors an MH-like pattern over the Western U.S., although associated atmospheric drying is stronger in CAM5 than in ECHAM5.

### Water tagging and land-atmosphere recycling

The detailed hydrological processes controlling the MH remain unclear. Therefore, this section employs water tagging experiments with the meteorological fields nudged to ERA5 (Methods) to identify the critical evaporation source regions contributing to the moisture deficit over the Western U.S. In the first experiment, 54 tagging regions spanning the global domain are defined, each representing a 60-by-20-degree zone (Methods). The total moisture amount or its long-term trend over the Western U.S. is then the sum of evaporation contributions from each region. The model reasonably simulates observed humidity and precipitation trends over the period, even at a lower resolution where topographic effects are not fully captured. This provides confidence that the model’s hydrological cycle reflects realistic features to some extent when the meteorological fields are nudged to ERA5.

**Fig. 8:**
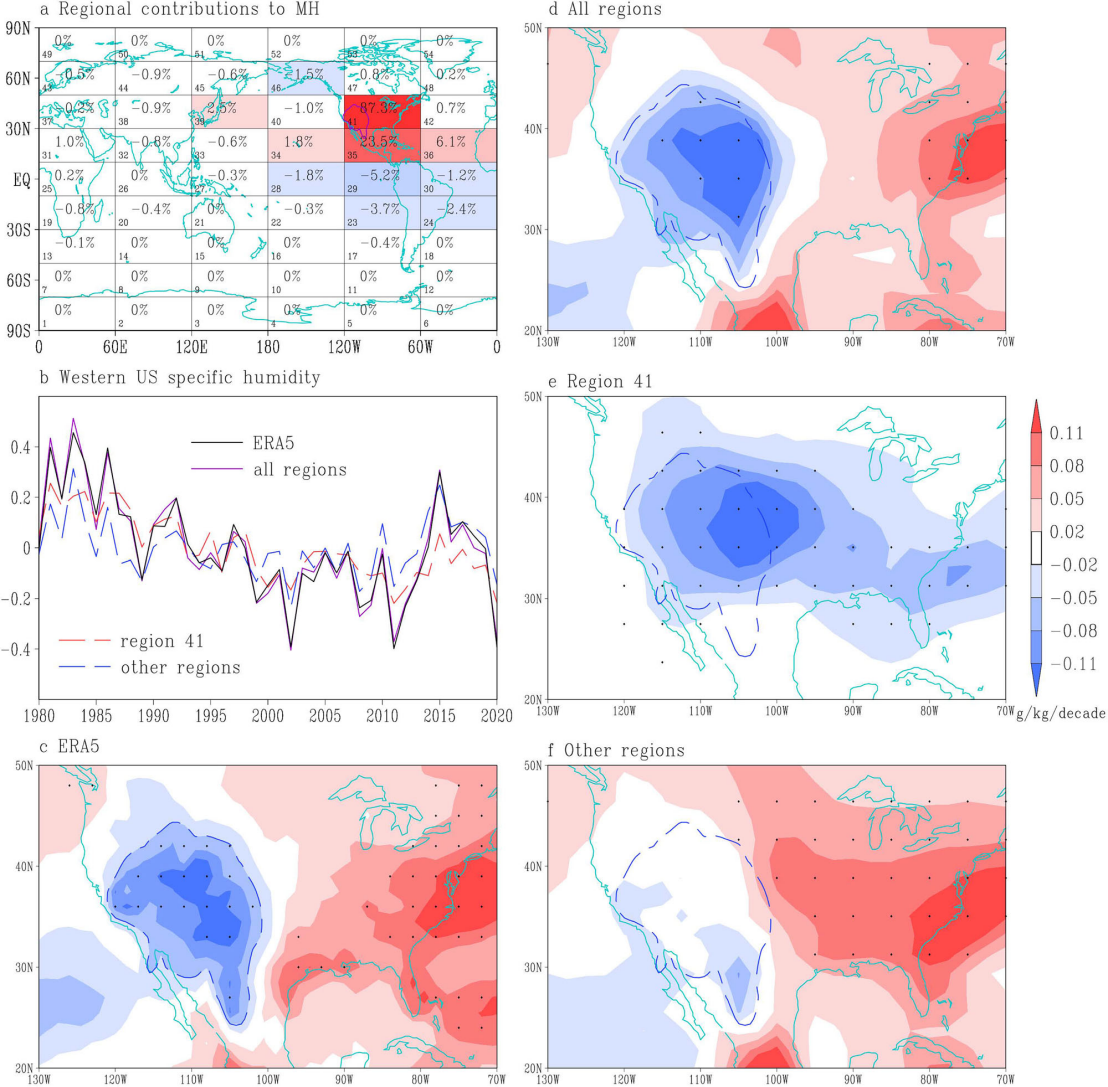
Contribution of each source region to the observed moistening hole (MH) trend from 1980 to 2020. **a** Fraction of the trend of annual mean lower-tropospheric (averaged between 650-1000 hPa) specific humidity over the Western U.S. (averaged within the purple contour) from 1980 to 2020, contributed by each tagging region in the global tagging experiment. For each tagging region, the number in the lower-left corner indicates its sequence from 1 to 54. The second percentage indicates the contribution of each source region to the linear trend of annual mean lower-tropospheric specific humidity over the Western U.S. from 1980 to 2020. Darker colors indicate higher contributions. Red indicates a positive contribution (>1%) to the trend, while blue (<–1%) indicates a negative contribution to the atmospheric drying trend over the Western U.S. **b** Time series of annual mean lower-tropospheric (averaged from 650 to 1000 hPa) specific humidity averaged over the Western U.S. (within the dashed blue contour in c) from 1980 to 2020, in ERA5 (black), and in the global tagging experiment: all regions (purple), Zone 41 (red dashed), and the other 53 regions (blue dashed). Spatial patterns of linear trends in annual mean lower-tropospheric specific humidity from (**c**) ERA5, and from the global tagging experiment: **d** all tagging regions, **e** Zone 41 only, and **f** the other 53 regions. Trends significant at the 95% confidence level are marked with dots.

Climatologically, 13 key regions spanning the North Pacific to the North Atlantic collectively contribute almost 99% of the total atmospheric moisture over the Western U.S. Of these 13 regions, Zone 35 (23%, over Central America) and Zone 41 (20%, over North America) contribute the most to the climatological moisture amount (Supplementary Fig. 13a). Regarding the fraction of the long-term trend of the MH contributed by each source region, Zone 41 (over North America) stands out, contributing 88%, followed by Zone 35 (24%) (Fig. 8a and Supplementary Fig. 13b; note that some regions contribute opposite trends, e.g., the tropical Eastern Pacific, -5%). It is clear that moisture evaporating from Zone 41 is critical for shaping the observed MH pattern in ERA5. The remaining 53 zones primarily capture year-to-year variability in atmospheric specific humidity over the Western U.S. (Fig. 8b) rather than long-term trends in the region. This result is consistent with our moisture budget analysis, suggesting that local moisture sources are likely the primary drivers of atmospheric drying associated with the MH during this period.

Since Zones 35 and 41 cover large areas, spanning Central America to North America, a second experiment refined these regions by splitting them into smaller 10-by-10-degree areas (Supplementary Fig. 14a). These finer tagging runs reveal that the region contributing most to the MH is primarily over the Western U.S. (30–40°N, 110°W–120°W), particularly over land areas near New Mexico. This zone alone accounts for 45% of the atmospheric drying of the MH over the 41-year period. Furthermore, the trend of moisture origin from this tagging zone, primarily responsible for drying the atmosphere, shifts slightly northeastward. This finding is supported by our moisture budget and correlation analysis using interannual data (Fig. 3 and Supplementary Figs. 15 and 16, Methods), which show that atmospheric specific humidity over the Western U.S. is highly correlated with evaporation over New Mexico in ERA5.

## Discussion

**Fig. 9:**
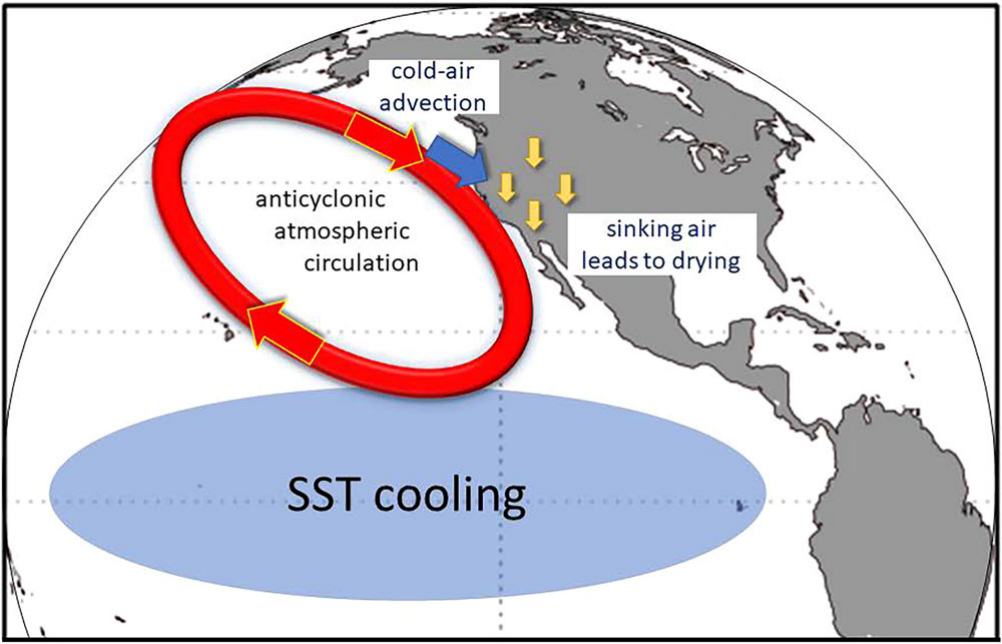
A dynamical process linking tropical Pacific sea surface temperature (SST) cooling to the moistening hole (MH). Schematic diagram illustrating the formation of the MH, in which tropical SST cooling plays a key role in generating the entire process.

Our study integrates comprehensive analyses of reanalysis datasets, large-ensemble historical simulations, AMIP runs, and water tagging experiments with meteorological fields nudged to reanalysis. Collectively, our analyses reveal that tropical SST cooling-driven large-scale circulation trends, manifested as a barotropic high pressure system over the North Pacific near the U.S. West Coast, create conditions conducive to anomalous sinking motion over the Western U.S. over the past four decades. This subsidence, primarily resulting from enhanced cold-air advection, suppresses precipitation and reduces land evaporation, weakening the region’s land–atmosphere moisture recycling and intensifying atmospheric drying (Fig. 9). These processes help explain the establishment of the MH over the West, in contrast to the hemisphere-wide long-term moistening induced by global warming over the same period. Because most climate models forced by anthropogenic forcing fail to reproduce the observed tropical Pacific SST cooling trend, they are unable to capture the observed MH pattern. Our analysis offers a valuable perspective that complements existing studies, which primarily emphasize the role of anthropogenic forcing in driving the recent drying trend in the region through various thermodynamic processes^15–24^. In addition, we find that long-term changes of vertical motion may play similar roles in other regions. Over the past four decades, moderate atmospheric drying has also been observed in Central Africa and the Amazon, accompanied by strengthening sinking motion (Supplementary Fig. 17). This highlights a common mechanism whereby atmospheric vertical motion shapes atmospheric moisture over long-term timescales across different climate zones.

Our results also have important implications for understanding observed hydroclimate anomalies in the Western U.S. over the past decades, including increased soil dryness, reduced groundwater levels and river discharge, more frequent wildfires, prolonged agricultural droughts, and heightened ecosystem stress since 2000^20,58^. Given the dominant role of La Niña-like SST cooling processes in driving the recent MH pattern, a potential weakening or reversal of the tropical Eastern Pacific SST cooling trend could mitigate the drying trend in the coming decade. Moreover, future global warming, favoring an El Niño-like SST pattern over the Eastern Pacific, may even reverse this drying trend if the climate responses in current models prove reliable^52^. Since it remains unclear whether the observed SST cooling (or minimal warming) is driven by internal variability or external radiative forcing, understanding the tropical Eastern Pacific SST responses to global warming is critical for projecting their impacts on moisture variability over the Western U.S.

Although land-atmosphere interactions are crucial for long-term atmospheric humidity changes in the Western U.S. ^24^, they appear to play a more complex role compared to changes in vertical motion and precipitation. Specifically, the land-atmosphere system in the region functions like a capacitor in an electric circuit, transferring precipitation-induced changes from large-scale circulation variability to atmospheric moisture and, in turn, enabling local control of precipitation and evaporation through atmospheric moisture conditions. In addition, our analysis is primarily based on annual-mean data, and further examination of the seasonality of MH formation indicates that land-atmosphere interactions are characterized by strong inter-seasonal connections, which may help bridge the effect of atmospheric conditions from one season to the next (Methods). However, the ability of current models to accurately simulate these two way and cross-season interactions, particularly in the absence of reanalysis data nudging, remains uncertain. This stresses the urgent need to develop robust metrics for evaluating models’ performance in capturing these observed processes, ensuring their reliability for future hydroclimate projections in the Western U.S. and other arid regions^59^.

## Methods

### Data and model experiments

The datasets used in this study include ERA5^60^ and MERRA2^61^ reanalysis data, precipitation data from GPCP^62^ and GPCC^63^, SST from ERSSTv5^64^, and CPC soil moisture^65^, all at monthly resolution. Additionally, we analyze the CESM2 Large Ensemble (CESM2-LE^66^), which comprises 100 realizations of historical (1979–2014) and future (2015–2020) simulations. The historical simulations are forced by observed radiative forcing, while future simulations follow the SSP3-7.0 emissions scenario, both following protocols established by the Coupled Model Intercomparison Project Phase 6 (CMIP6^67^). In particular, members 1–50 use the original CMIP6 biomass-burning aerosol emissions, whereas members 51–100 use a smoothed version of the CMIP6 biomass-burning dataset. We do not find any significant differences in the simulated MH trend pattern between these two subgroups. In CESM2-LE, two initialization schemes across the 100 members allow for significant internal variability to arise^66^.

To determine whether the forced response in CESM2-LE reflects a broader consensus among climate models, we analyze 27 models from the CMIP6 ensemble^67^, combining historical runs (1980–2014) with SSP5-8.5 simulations (2015–2020), as more models provide the necessary fields under SSP5-8.5. While slight differences exist between the SSP5-8.5 and SSP3-7.0 scenarios during the overlapping period (2015–2020), these are unlikely to influence our qualitative comparison of model responses.

The MH region is defined as the significant specific humidity drying over the Western U.S., as shown in Fig. 3a. To construct a domain-averaged specific humidity index representing MH variations, we only consider reanalysis and model data in the lower troposphere (below 650 hPa) and above the surface elevation.

### Significance of the MH pattern in various datasets

To evaluate whether the MH is a significant feature in both observations and reanalysis products, we analyze two in-situ monthly observational datasets in their latest versions: Integrated Global Radiosonde Archive v2.2 (IGRA2)^68^ and Met Office Hadley Center International Surface Dataset of Humidity (HadISDH, blended v 1.5)^69^. HadISDH is a low-resolution (5° × 5°) gridded dataset focused primarily on surface humidity over land and nearby ocean regions, derived from quality-controlled station measurements. IGRA2 is a global network of radiosonde observations providing measurements of geopotential height, temperature, wind, and vapor pressure at numerous standard pressure levels (e.g., 1000 hPa, 850 hPa, 700 hPa, 500 hPa, 400 hPa, and 300 hPa). With bias correction applied, IGRA2 radiosonde data has been assimilated into ERA5^60^. Specific humidity in IGRA2 is calculated from vapor pressure using Eq. 1. Due to the high elevation across the Western U.S., our analysis focuses on the 700 hPa, 500 hPa, and 400 hPa levels from IGRA2, as well as surface (2 m) specific humidity from HadISDH. Radiosonde data availability at other levels, especially over the Western U.S., is limited.

1 $${specific}\,{humidity}=\frac{0.622\times {vapor}\,{pressure}}{{surface}\,{pressure}-0.378\times {vapor}\,{pressure}}$$

Monthly radiosonde records at 700hPa are available twice daily (00 and 12 UTC). To construct monthly means, we use only those months with at least 15 valid daily observations per month; all other months are treated as missing data. For long-term trend analysis over the 1980–2020 period (492 months), we retain only stations with no more than 48 missing months, following quality control practices used in previous studies^9^. At each site, the 00 and 12 UTC values are averaged within each month to produce monthly means.

Applying these criteria, we identify 41 IGRA2 stations with broad spatial coverage across the domain (20°–50°N, 230°E–290°E), including 12 stations located in the Western U.S. (Supplementary Fig. 1a). All these 12 stations have nearly complete records, with only 2–3 missing months at two sites. Thus, the specific humidity fields derived from IGRA2 offer excellent temporal coverage for the 41-yr period. We interpolate these monthly station values, excluding those missing months, to a regular grid for each month using Laplace interpolation, and then compute annual means and linear trends over the entire land area. The long-term trend of 700hPa specific humidity over the Western U.S., based on regridded IGRA2 data (−0.036 g/kg/decade, Supplementary Fig. 1a), is ~62% of the trend estimated from ERA5 ( − 0.058 g/kg/decade, Supplementary Fig. 1b). However, because ERA5 incorporates more comprehensive observational sources, including all bias-corrected radiosonde data, a more direct comparison is to extract 700hPa specific humidity from ERA5 at the same 12 IGRA2 sites and compare it with the IGRA2 measurements. This comparison shows that the two time series have very similar magnitude and the drying trend in IGRA2 ( − 0.042 g/kg/decade) is about 93% of that in ERA5 ( − 0.045 g/kg/decade), indicating strong agreement between the two datasets over the 12 locations (Supplementary Fig. 1d). In contrast, MERRA2 shows only slight drying over the Western U.S., embedded within a broader moistening trend across North America (Supplementary Fig. 1c). This highlights a limitation in MERRA2’s ability to fully capture the observed intensity of the MH change, although it reproduces the spatial pattern to some extent.

At the surface, ERA5 also exhibits stronger drying than HadISDH, while MERRA2 shows only a weak drying signal over the Western U.S. (Supplementary Fig. 2). Notably, IGRA2 reveals a vertically coherent MH structure extending from the surface to the upper troposphere (~400hPa), whereas ERA5 shows possibly stronger but shallower drying trends, and MERRA2 shows only weak drying in the lower troposphere (Supplementary Fig. 2). This difference is further illustrated by the correlation in specific humidity at 700 hPa (Supplementary Fig. 1d). The correlation between IGRA2 and ERA5 reaches 0.87 over the 1980–2020 period, while the correlation between IGRA2 and MERRA2 is slightly lower at 0.76. When all three time series are detrended, the correlations between IGRA2 and both reanalyzes become nearly identical. This suggests that while MERRA2 reproduces the year-to-year variability of radiosonde-measured specific humidity reasonably well, it does not capture its long-term trend as effectively as ERA5, likely due to differences in the physical parameterization schemes of the underlying general circulation models used in each system^70^. Therefore, to examine the long-term trend of the MH feature in our study, we primarily rely on ERA5 in our diagnostic analysis and nudging experiments.

Although ERA5 and IGRA2 are consistent in capturing the MH feature in the lower troposphere, they exhibit quite different drying patterns in the upper troposphere over the Western U.S. This discrepancy is likely related to the temperature bias correction applied in ERA5 to address a radiosonde warm bias in the upper troposphere during the 1980s^60^. Because IGRA2 vapor pressure is derived from direct measurements of temperature and relative humidity^71^, this warm bias can induce a wet bias over land in the 1980s, leading to an apparent strong drying trend over the period. We also examined JRA55^72^, which shows results more consistent with ERA5 than with MERRA2 across all vertical levels, strengthening confidence that the MH is a robust feature in the lower troposphere rather than in the upper troposphere (Supplementary Fig. 18). Nevertheless, we note that ERA5 captures the MH at the drier end of the spectrum compared with all reanalyzes considered (ERA5, JRA55, MERRA2, IGRA2), and it remains unclear whether this difference reflects inhomogeneities in the original observations or a real physical signal. In this context, our primary aim is to qualitatively investigate the underlying mechanisms of the MH rather than to quantify the magnitude of its long-term trend.

### Diagnostic analysis of vertical motion trends

To investigate processes contributing to long-term trends of vertical motion in the extratropics, we apply a diagnostic analysis based on the traditional quasi-geostrophic omega equation^73^ in pressure coordinates (Eq. 2).

2 $$\left({\nabla }_{p}^{2}+\frac{{f}_{0}^{2}}{\sigma }\frac{{\partial }^{2}}{{\partial p}^{2}}\right)\omega=\frac{{f}_{0}}{\sigma }\frac{\partial }{\partial p}\left[{V}_{g}\cdot {\nabla }_{p}\left({\zeta }_{g}+f\right)\right]+\frac{{R}_{d}}{\sigma p}{\nabla }_{p}^{2}\left({V}_{g}\cdot {\nabla }_{p}T\right)+\frac{{R}_{d}}{\sigma p}{\nabla }_{p}^{2}(-{{{\rm{Q}}}})$$

where ${\nabla }_{p}^{2}$ is the Laplacian operator in pressure coordinate, ${f}_{0}$ is the Coriolis constant at the same latitude where *ω* is being calculated, *σ* is the static stability parameter, *ω* is the vertical velocity, ${V}_{g}$ is the geostrophic wind vector, ${\nabla }_{p}$ is the horizontal gradient operator on a pressure surface, ${\zeta }_{g}$ is the geostrophic relative vorticity, *f* is the Coriolis parameter, ${R}_{d}$ is the gas constant for dry air, *p* is the pressure, *T* is the temperature, and Q is the diabatic heating rate in units of K/s.

As encapsulated by Eq. 2, vertical motion is driven by three primary terms: (term 1) the vertical variation of geostrophic absolute vorticity advection by the geostrophic wind$:\frac{\partial }{\partial p}\left[{V}_{g}\cdot {\nabla }_{p}\left({\zeta }_{g}+f\right)\right]$, (term 2) the Laplacian of temperature advection by the geostrophic wind $\left({V}_{g}\cdot {\nabla }_{p}T\right)$, and (term 3) Laplacian of diabatic heating. Using ERA5 daily data, we compute terms 1 and 2 at each grid point on a daily basis. We then calculate the annual means of these terms and analyze their long-term trends to assess their relative contributions to vertical motion trends over the Western U.S. For example, to calculate term 2, daily geopotential height at each grid point is used to derive geostrophic winds, which are then multiplied by the horizontal gradient of daily temperature to obtain temperature advection by the geostrophic wind. The annual mean value of this term is then computed by averaging the daily values over the corresponding time period.

Because diabatic heating is normally calculated as a residual term in the temperature equation (once 6-h winds and temperature are given), the trend in diabatic heating cannot be treated as an independent diagnostic for understanding trends in vertical motion, as vertical motion is already used in its calculation. Following the residual diagnosis method^74^, we present the long-term trend of annual mean diabatic heating over the Western U.S. to demonstrate that its role is consistent with, rather than offsetting, the contribution of term 2 in driving vertical motion in the region (Supplementary Fig. 5).

### Climate drivers of atmospheric humidity over the Western U.S. on interannual timescales

The observed relationship between long-term changes in vertical motion, tropical Pacific SST, and atmospheric moisture trends over the Western U.S. may reflect covarying secular trends across all variables, rather than physically meaningful causality. A useful way to assess this is by examining their relationships on interannual timescales, with long-term trends removed. If similar patterns emerge with a sufficiently large sample size, it would strengthen confidence in the robustness of the inferred long-term relationship based on the 41-year trends.

To investigate the interannual connection between lower-tropospheric specific humidity over the Western U.S. and large-scale climate drivers, we construct a regional specific humidity index averaged over the area exhibiting the strongest MH feature (dashed blue contour in Supplementary Fig. 15). This index is then correlated with large-scale fields known to influence humidity changes in the region (Supplementary Fig. 15). To minimize the impact of secular trends in the datasets and focus on robust physical links, we use detrended data for the correlation analysis in both ERA5 and model results. ERA5 reveals a relatively weak connection between humidity and fields outside the U.S. West, with stronger linkages to local evaporation and precipitation rates. Specifically, reduced evaporation, particularly over land areas near New Mexico, favors atmospheric drying, and a similar relationship is evident between precipitation and humidity. The relationship with IVT is primarily confined to the Western U.S., with little extension to upstream regions over the tropical or subtropical Pacific. This indicates the dominant role of local processes in governing a wide range of humidity variations, from interannual to interdecadal timescales, and may also influence the 41-year long-term trend.

Additionally, tropical Eastern Pacific SST anomalies are strongly associated with atmospheric humidity over the Western U.S. (Supplementary Fig. 15d). Vertical motion over the region appears to exert direct forcing on year-to-year humidity variability (Supplementary Fig. 15f). In ERA5, this year-to-year relationship, where a wetter atmosphere coincides with low pressure and warm SST to the west and local upward motion, mirrors the coherent variation of the same variables on long-term timescales (Figs. 1 and 2) in its reversed phase. This reinforces the causal relationship, inferred from the diagnostic analysis of the omega equation, between tropical Eastern Pacific SST trends and atmospheric drying in the region over the past four decades, mediated by the long-term trend in vertical motion over the Western U.S. CESM2-LE shows correlation patterns similar to those in ERA5 (Supplementary Fig. 16) albeit with weaker magnitude, indicating that the model can reasonably capture observed year-to-year variations associated with specific humidity fluctuations over the Western U.S.

### AMIP runs examining the role of tropical SST cooling in driving the MH

To assess the role of tropical forcing in driving the MH feature, we conduct two AMIP-type simulations using CAM5 (the same atmospheric component as in iCESM, with F19 resolution and 32 vertical levels) and ECHAM5^75^ (T42 resolution with 19 vertical levels). For each model, we perform two sets of runs: (1) a control run with climatological SST and sea ice and fixed anthropogenic forcing, and (2) a sensitivity run with the same setup (boundary forcing and initialization) but with an imposed SST cooling pattern added in the tropical Eastern Pacific (10˚S-10˚N, 170˚E-270˚E). We integrate these experiments for 30 years in both models to examine whether the responses are robust across two distinct model physics. To mimic the observed SST cooling trend over the past 41 years, we impose cooling using an idealized pattern with a maximum amplitude of −1 °C at the center and zero cooling along the edges (blue contours in Fig. 7a).

### Integrated, coupled modeling approach using iCESM with water tagging and “replay” of observations

To better understand the observed hydrological cycle in North America from 1980 to 2020 and to examine atmosphere-ocean-land interactions, we use iCESM1.2 (at 1.9° × 2.5° resolution) in this study^45^, which allows us to quantify the impacts of observed atmospheric and boundary forcing on moisture, precipitation, and key land processes across North America. As our analysis represents an initial attempt to apply this nudging approach, no established protocol exists.

Following a standard procedure (Eq. 3) of the NCAR Data Assimilation Research Testbed (DART) system, we apply full nudging (α = 1) to constrain the model to evolve as closely as possible to ERA5.

$$\frac{{dx}(t)}{{dt}}=F\left(x\right)+{F}_{{nudge}}$$

3 $${F}_{{nudge}}=\alpha (R\left({t}^{{\prime} }{next}\right)-x\left(t\right))/({t}^{{\prime} }{next}-t)$$

where x(t) denotes the model state (e.g., U, V, T, and Q in iCESM1) at model time step t. F(x) represents the model’s internally generated tendency, and ${F}_{{nudge}}$ is the nudging term that drives the model toward the ERA5 fields at the next analysis time step, R(t’next), relative to the current state x(t). In our setup, R(t′next) is updated every 6 h, while x(t) is updated every 30 minutes. The coefficient α determines the nudging strength, ranging from 1 to 0. Full nudging (α = 1) allows the model to closely follow ERA5 and ensures that the hydrological cycle and water tagging results are well constrained, whereas α = 0 means no nudging. To examine the sensitivity of simulated moisture changes to the nudging settings, we initially explore three different configurations:

Setting 1: Nudging the model’s 3D wind (U, V) and temperature (T) fields (from the surface to the top of the atmosphere) and near-surface humidity (Q) to closely follow 6-h ERA5 reanalysis data, while imposing monthly SST and sea ice from ERA5 as boundary conditions. Anthropogenic forcing (e.g., CO2, CH4, N2O, F11&12) is set according to the CMIP6 scenario from 1980 to 2020. This ensures the model accurately captures observed atmospheric, oceanic and radiative forcing conditions.

Setting 2: Turning off the nudging for specific humidity (no Q nudging) at the surface while keeping all other settings the same as in Setting 1.

Setting 3: Turning off the nudging for specific humidity (no Q nudging) at the surface (same as in Setting 2) and additionally keeping radiative forcing (e.g., CO2, CH4, N2O, F11 & F12) constant at 1979 levels, while maintaining all other settings as in Setting 1. All these configurations produce very similar 41-year variations in atmospheric specific humidity over the Western U.S (Supplementary Fig. 19). We also calculate the correlation between the annual mean precipitation in each simulation and that in GPCP (Supplementary Fig. 20). The results show that all three settings effectively reproduce precipitation changes across the region, especially over the Western U.S., with correlation coefficients reaching 0.8 to 0.9. This gives us confidence that the model, regardless of nudging and radiative forcing settings, can reliably capture hydrologic variations over the region.

In addition, iCESM1.2 includes a water tagging function, which enables the tracking of atmospheric moisture from its source regions to its ultimate sinks. This capability allows us to monitor the complete lifecycle of water vapor, from evaporation to precipitation, along with intermediate hydrological processes, offering insights into both local and remote sources of moisture and precipitation variability over North America. It should also be noted that, because tracking multiple rounds of recycling is not feasible in iCESM1.2, this tagging approach tracks only the immediate, direct moisture contribution to the most recent precipitation event, rather than moisture that has undergone multiple cycles of precipitation and re-evaporation. Because nudging Q in the near-surface layer provides a better constraint on the simulation of evaporation, our analysis primarily uses the simulation setup described in Setting 1.

We also conduct three additional experiments by relaxing the nudging strength (α) to evaluate the sensitivity of MH drying to the nudging procedure:

i. Nudging only U, V, and T everywhere (no Q nudging), with anthropogenic forcing fixed at 1979 and nudging strength α = 0.5.
ii. Same as (i), but with weaker nudging strength α = 0.2.
iii. Full nudging (α = 1) of U, V, and T (no Q nudging) everywhere except over the Western U.S. (no nudging applied within the box, 22.5°N-47.5°N, 237.5°E-262.5°E, shown in Supplementary Fig. 21f). Across these experiments (Supplementary Fig. 21), the MH feature remains highly robust regardless of the nudging setup. We also note that as nudging strength decreases (from 0.5 to 0.2 to no nudging over the Western U.S.), the magnitude of the MH trend declines from ~90% to ~50% of that in Setting 1. All tagging results from these runs further indicate that local sources are critical to MH formation and are sensitive to the ERA5 meteorological fields. Thus, any potential bias in ERA5 may also be imparted to our nudging results.

### Experimental design: global and regional tagging experiments

Under Setting 1, we first conduct a 41-year (1980–2020) iCESM simulation with global nudging and 54 predefined tagging regions spanning the globe. Each tagging region covers 60° longitude × 20° latitude, with regions sequentially labeled west-to-east within a latitude band before progressing from south to north. For instance, Region 1 spans 90°S–70°S, 0°–60°E, while Region 54 spans 70°N–90°N, 300°–360°E. This global tagging ensures seamless spatial coverage.

To refine our understanding of moisture sources over North America, we conduct a second simulation focusing on regions 35 and 41, which cover Central and North America (10°–50°N, 240°E–300°E). These regions are subdivided into 24 smaller domains (10° × 10° resolution) to enable finer-scale differentiation of moisture sources. All other settings remain consistent with the global tagging experiment. As most atmospheric fields (winds and temperature) are constrained by ERA5, a single realization is sufficient for both the global and regional tagging experiments.

### Evaluation of our nudging experiments against observations

As the water supply for land evaporation comes primarily from precipitation, we also examined the moisture sources driving the climatology and trends of annual mean precipitation over the Western U.S. in the global tagging experiment (Supplementary Fig. 22). For climatological precipitation, the fractional contributions from each region resemble those for atmospheric humidity (Supplementary Fig. 13). However, the significance of each source in contributing to precipitation trends over the Western U.S. differs from that for atmospheric specific humidity trends. While up to 88% of the atmospheric specific humidity trend is locally controlled, the local source accounts for only 34% of the precipitation trend. Instead, contributions from remote regions, such as the subtropical Pacific, become more apparent for the precipitation trend. This suggests that long-term changes in atmospheric humidity over the Western U.S. are more sensitive to local recycling processes, whereas precipitation trends are jointly driven by changes in local and remote moisture sources, as well as large-scale circulation variations that mediate moisture transport from remote areas.

By nudging ERA5 fields in our experiments, we observe reasonable consistency between precipitation and evaporation trends from ERA5 and our simulated results. The temporal variability of domain averages for these two variables in ERA5 is well simulated (Supplementary Figs. 23 and 24), albeit with a slight eastward shift in the centers of maximum decline. This shift is spatially collocated with a trend of downward motion in the nudging run. While ERA5 winds are imposed as accurately as possible, discrepancies in vertical motion persist due to limitations in model resolution and coarse topography settings. Although this slight shift may indicate a minor bias in the model configuration, it strongly suggests that downward motion plays a key role in shaping precipitation and evaporation over the Western U.S.

Precipitation and evaporation variations in various datasets, including ERA5, are known to contain significant uncertainty. To better understand these uncertainties, we compare trends in these two fields across multiple datasets. Although different datasets show diverse results, they consistently illustrate a 41-year declining trend in both precipitation and evaporation over the Western U.S., with ERA5 exhibiting the strongest decline. In particular, GPCP and MERRA2, which incorporate instrument-based and satellite observations, respectively, exhibit a similar decreasing trend in precipitation and evaporation over the region as seen in our nudging run. Additionally, our model, when nudged by ERA5 reanalysis, shows a high correlation between its precipitation and GPCP rainfall on an annual basis over the study period (Supplementary Fig. 20), indicating that the simulation of the hydrological cycle, including precipitation and evaporation, in the model closely captures observed characteristics.

### Seasonality of the MH pattern

Consistent with previous work^9^, we find that the MH is a year-round phenomenon, with the strongest drying signals occurring in MAM and JJA (Supplementary Fig. 25). Seasonal trends of IVT in and around the MH region show no clear patterns in any season (Supplementary Fig. 26), suggesting that local precipitation and evaporation changes primarily shape the seasonal drying signal.

Analysis of seasonal trends in vertical motion over the MH region indicates that strong sinking trend prevails in almost all seasons except JJA (Supplementary Fig. 25). At first glance, these trends in vertical motion do not appear consistent with changes in specific humidity. However, as shown in Supplementary Fig. 25e–l, evaporation and precipitation exhibit strong lead–lag correlations. As noted previously^13,76^, summer evaporation mainly responds to preceding precipitation, since the Western U.S. acts as a climatological moisture source during summer. This suggests that, throughout the year, precipitation exerts both immediate and delayed effects on local evaporation, implying that circulation-driven changes in precipitation can induce lagged responses in evaporation and atmospheric humidity through local recycling. Consequently, the pronounced drying of specific humidity in JJA over the Western U.S. may partly result from circulation trends in preceding seasons.

### Significance test

To assess and visualize the significance of trends, correlations and composite differences across spatial grids, we apply a statistical criterion that accounts for the false discovery rate^77^. For correlations based on annual (monthly) mean data, we use a threshold of ±0.4 ( ± 0.2), which are statistically significant at the 95% confidence level when considering the effective degrees of freedom, generally ranging from 35 to 39 for our annual mean data.

## Supplementary information

Supplementary Information

Transparent Peer Review file

## Data Availability

The ECMWF ERA5 reanalysis is available at (https://ecmwf.int/en/forecasts/datasets/reanalysis-datasets/era5). The ERSSTv5 is assessible via NOAA (https://ncei.noaa.gov/products/extended-reconstructed-sst). GPCC is available from (https://psl.noaa.gov/data/gridded/data.gpcc.html). GPCP can be accessed through (https://psl.noaa.gov/data/gridded/data.gpcp.html). MERRA2 is hosted at (https://gmao.gsfc.nasa.gov/reanalysis/MERRA-2/data_access/) CESM2-LE is available for download from NCAR (https://cesm.ucar.edu/community-projects/lens2). Monthly means of mass-consistent vertical integral of northward water vapor flux derived from ERA5 reanalysis is accessible from (https://cds.climate.copernicus.eu/cdsapp#!/dataset/derived-reanalysis-energy-moisture-budget?tab=overview). IGRA2 can be obtained via (https://www.ncei.noaa.gov/products/weather-balloon/integrated-global-radiosonde-archive). HadISDH is publicly available at (https://climatedataguide.ucar.edu/climate-data/global-near-surface-humidity-data-hadisdh). CPC soil moisture data can be obtained from (https://psl.noaa.gov/data/gridded/data.cpcsoil.html). Our AMIP runs generated in this study have been deposited in the Zenodo database under accession code (https://zenodo.org/records/18827534).

## Code availability

Since the diagnostic analysis based on the omega equation is unique, we provide the relevant code, along with all other code, at: (https://github.com/qinghuading513/diagnostic-of-Q-G-omega-equation^78^).

## References

[1] Held, I. M. & Soden, B. J. Water vapor feedback and global warming. *Ann. Rev. Energy Environ.* **25**, 441–475 (2000).

[2] Trenberth, K. E., Fasullo, J. & Smith, L. Trends and variability in column integrated atmospheric water vapor. *Clim. Dyn.* **24**, 741–758 (2005).

[3] Santer, B. D. et al. Identification of human-induced changes in atmospheric moisture content. *Proc. Natl. Acad. Sci. USA.* **104**, 15248–15253 (2007). 17881573 10.1073/pnas.0702872104 PMC1986574

[4] Dai, A. Increasing drought under global warming in observations and models. *Nat. Clim. Change* **3**, 52–58 (2013).

[5] Sherwood, S. & Fu, Q. A drier future? *Science* **343**, 737–739 (2014). 24531959 10.1126/science.1247620

[6] Ma, J. et al. Hydrological cycle changes under global warming and their effects on multiscale climate variability. *Ann. N. Y. Acad. Sci.* **1472**, 21–48 (2020). 32223020 10.1111/nyas.14335

[7] Douville, H. et al. *Water cycle changes. In Climate Change 2021: The Physical Science Basis. Contribution of Working Group I to the Sixth Assessment Report of the Intergovernmental Panel on Climate Change* (Cambridge Univ. Press, 2021).

[8] Dunn, R. J. H., Willett, K. M., Ciavarella, A. & Stott, P. A. Comparison of land surface humidity between observations and CMIP5 models. *Earth Syst. Dynam.* **8**, 719–747 (2017).

[9] Simpson, I. R. et al. Observed humidity trends in dry regions contradict climate models. *Proc. Natl. Acad. Sci. USA.* **121**, e2302480120 (2024). 38147646 10.1073/pnas.2302480120 PMC10769846

[10] Howitt, R., Medellin-Azuara, J., MacEwan, D., Lund, J. & Sumner, D. A. Economic analysis of the 2014 drought for California agriculture. 1–20 (Center for Watershed Sciences, University of California, Davis, California, 2014).

[11] Faunt, C. C., Sneed, M., Traum, J. & Brandt, J. T. Water availability and land subsidence in the Central Valley, California, USA. *Hydrogeol. J.* **24**, 675–684 (2016).

[12] Rodell, M. et al. Emerging trends in global freshwater availability. *Nature* **557**, 651–659 (2018). 29769728 10.1038/s41586-018-0123-1 PMC6077847

[13] Jacobson, T. W. P. et al. An unexpected decline in spring atmospheric humidity in the interior Southwestern United States and implications for forest fires. *J. Hydrometeor.* **25**, 373–390 (2024).

[14] Held, I. M. & Soden, B. J. Robust responses of the hydrological cycle to global warming. *J. Clim.* **19**, 5686–5699 (2006).

[15] Seager, R. et al. Model projections of an imminent transition to a more arid climate in Southwestern North America. *Science* **316**, 1181–1184 (2007). 17412920 10.1126/science.1139601

[16] Dai, A. Drought under global warming: a review. *WIREs Clim. Change* **2**, 45–65 (2011).

[17] Cook, B. I. et al. Unprecedented 21st century drought risk in the American Southwest and Central Plains. *Sci. Adv.* **1**, e1400082 (2015). 26601131 10.1126/sciadv.1400082 PMC4644081

[18] Diffenbaugh, N. S., Swain, D. L. & Touma, D. Anthropogenic warming has increased drought risk in California. *Proc. Natl. Acad. Sci. USA.* **112**, 3931–3936 (2015). 25733875 10.1073/pnas.1422385112 PMC4386330

[19] Douville, H. & Plazzotta, M. Midlatitude summer drying: an underestimated threat in CMIP5 models? *Geophys. Res. Lett.* **44**, 9967–9975 (2017).

[20] Williams, A. P. et al. Large contribution from anthropogenic warming to an emerging North American megadrought. *Science* **368**, 314–318 (2020). 32299953 10.1126/science.aaz9600

[21] Ault, T. R. On the essentials of drought in a changing climate. *Science* **368**, 256–260 (2020). 32299944 10.1126/science.aaz5492

[22] Allan, R. P. & Douville, H. An even drier future for the arid lands. *Proc. Natl. Acad. Sci. USA.* **121**, e2320840121 (2024). 38157450 10.1073/pnas.2320840121 PMC10786295

[23] Zhuang, Y. et al. Anthropogenic warming has ushered in an era of temperature-dominated droughts in the western United States. *Sci. Adv.* **10**, eadn9389 (2024). 39504363 10.1126/sciadv.adn9389 PMC11540010

[24] Seager, R. et al. Dynamics of future soil moisture drought in Southwest North America: linkages across seasons in the ocean–atmosphere–land system. *J. Clim.* **38**, 2139–2154 (2025).

[25] Prein, A. F., Holland, G. J., Rasmussen, R. M., Clark, M. P. & Tye, M. R. Running dry: The U.S. Southwest’s drift into a drier climate state. *Geophys. Res. Lett.* **43**, 1272–1279 (2016).

[26] McKinnon, K. A., Poppick, A. & Simpson, I. R. Hot extremes have become drier in the United States Southwest. *Nat. Clim. Change* **11**, 598–604 (2021).

[27] Wang, H. et al. Potential caveats in land surface model evaluations using the US drought monitor: roles of base periods and drought indicators. *Environ. Res. Lett.* **17**, 014011 (2022).

[28] Cook, B. I. et al. Cold tropical Pacific sea surface temperatures during the late sixteenth-century North American megadrought. *J. Geophys. Res. Atmos.* **123**, 11307–11320 (2019).

[29] Steiger, N. J., Smerdon, J. E., Cook, B. I., Seager, R. & Williams, A. P. Oceanic and radiative forcing of medieval megadroughts in the American Southwest. *Sci. Adv.* **5**, eaax0087 (2019). 31355339 10.1126/sciadv.aax0087 PMC6656535

[30] McCabe, G. J., Palecki, M. A., Gray, S. & Betancourt, J. L. Pacific and Atlantic influences on multidecadal drought frequency in the United States. *Proc. Natl. Acad. Sci. USA.* **101**, 4136–4141 (2004). 15016919 10.1073/pnas.0306738101 PMC384707

[31] Schubert, S. et al. A U.S. CLIVAR project to assess and compare the responses of global climate models to drought-related SST forcing patterns: overview and results. *J. Clim.* **22**, 5251–5272 (2009).

[32] Wang, H., Schubert, S., Suarez, M. & Koster, R. The physical mechanisms by which the leading patterns of SST variability impact U.S. precipitation. *J. Clim.* **23**, 1815–1836 (2010).

[33] Kushnir, Y., Seager, R., Ting, M., Naik, N. & Nakamura, J. Mechanisms of tropical Atlantic SST influence on North American hydroclimate variability. *J. Clim.* **23**, 5610–5628 (2010).

[34] Ting, M., Kushnir, Y., Seager, R. & Li, C. Robust features of Atlantic multi-decadal variability and its climate impacts. *Geophys. Res. Lett.* **38**, L17705 (2011).

[35] Seager, R. et al. Ocean-forcing of cool season precipitation drives ongoing and future decadal drought in southwestern North America. *npj Clim. Atmos. Sci.* **6**, 141 (2023).

[36] Hamlet, A. F. & Lettenmaier, D. P. Effects of 20th century warming and climate variability on flood risk in the western US. *Water Resour. Res.* **43**, W06427 (2007).

[37] Affram, G. et al. Modulation of the Pacific Meridional Mode on the dipole pattern of the CONUS summertime precipitation. *Geophys. Res. Lett.* **51**, e2024GL109636 (2024).

[38] Meehl, G. A. & Hu, A. Megadroughts in the Indian monsoon region and Southwest North America and a mechanism for associated multidecadal Pacific sea surface temperature anomalies. *J. Clim.* **19**, 1605–1623 (2006).

[39] Liu, S., Hu, S. & Seager, R. The West Pacific teleconnection drives the interannual variability of autumn wildfire weather in the western United States after 2000. *Earth’s Future*, **12**, e2024EF004922 (2024).

[40] Delworth, T. L., Zeng, F., Rosati, A., Vecchi, G. A. & Wittenberg, A. T. A link between the hiatus in global warming and North American drought. *J. Clim.* **28**, 3834–3845 (2015).

[41] Lehner, F., Deser, C., Simpson, I. R. & Terray, L. Attributing the U.S. Southwest’s recent shift into drier conditions. *Geophys. Res. Lett.* **45**, 6251–6261 (2018).

[42] Seager, R. et al. Mechanisms of a meteorological drought onset: summer 2020 to spring 2021 in southwestern North America. *J. Clim.* **35**, 3767–3785 (2022).

[43] Cvijanovic, I. et al. Arctic sea-ice loss drives a strong regional atmospheric response over the North Pacific and North Atlantic on decadal scales. *Commun. Earth Environ.* **6**, 154 (2025).

[44] Nusbaumer, J., Wong, T. E., Bardeen, C. & Noone, D. Evaluating hydrological processes in the community atmosphere model version 5 (CAM5) using stable isotope ratios of water. *J. Adv. Model. Earth Syst.* **9**, 949–977 (2017).

[45] Brady, E. et al. The connected isotopic water cycle in the community earth system model version 1. *J. Adv. Model. Earth Syst.* **11**, 2547–2566 (2019).

[46] Harrington, T. S., Zhu, J. & Skinner, C. B. Terrestrial sources of summer Arctic moisture and the implication for Arctic temperature patterns. *npj Clim. Atmos. Sci.* **4**, 25 (2021).

[47] Ding, Q., Schweiger, A. & Baxter, I. Nudging observed winds in the Arctic to quantify associated sea ice loss from 1979 to 2020. *J. Clim.* **35**, 6797–6813 (2022).

[48] Trenberth, K. E. & Guillemot, C. J. Evaluation of the global atmospheric moisture budget as seen from analyses. *J. Clim.* **8**, 2255–2272 (1995).

[49] Dominguez, F., Miguez-Macho, G. & Hu, H. WRF with water vapor tracers: a study of moisture sources for the North American Monsoon. *J. Hydrometeorol.* **17**, 1915–1927 (2016).

[50] Zhang, Q. et al. Oceanic climate changes threaten the sustainability of Asia’s water tower. *Nature* **615**, 87–93 (2023). 36859582 10.1038/s41586-022-05643-8 PMC9977686

[51] Horel, J. D. & Wallace, J. M. Planetary-scale atmospheric phenomena associated with the Southern Oscillation. *Mon. Wea. Rev.* **109**, 813–829 (1981).

[52] Wills, R. C. J., Dong, Y., Proistosecu, C., Armour, K. C. & Battisti, D. S. Systematic climate model biases in the large-scale patterns of recent sea-surface temperature and sea-level pressure change. *Geophys. Res. Lett.* **49**, e2022GL100011 (2022).

[53] Byrne, M. P. & O’Gorman, P. A. Land–ocean warming contrast over a wide range of climates: convective quasi-equilibrium theory and idealized simulations. *J. Clim.* **26**, 4000–4016 (2013).

[54] Smith, D. et al. Role of volcanic and anthropogenic aerosols in the recent global surface warming slowdown. *Nat. Clim. Change* **6**, 936–940 (2016).

[55] Seager, R. et al. Strengthening tropical Pacific zonal sea surface temperature gradient consistent with rising greenhouse gases. *Nat. Clim. Change* **9**, 517–522 (2019).

[56] Meehl, G. A., Arblaster, J. M., Fasullo, J. T., Hu, A. & Trenberth, K. E. Model-based evidence of deep-ocean heat uptake during surface-temperature hiatus periods. *Nat. Clim. Change* **1**, 360–364 (2011).

[57] Yeager, S. G. et al. Reduced Southern Ocean warming enhances global skill and signal-to-noise in an eddy-resolving decadal prediction system. *npj Clim. Atmos. Sci.* **6**, 107 (2023).

[58] Milly, P. C. D. & Dunne, K. A. Colorado River flow dwindles as warming-driven loss of reflective snow energizes evaporation. *Science* **367**, 1252–1255 (2020). 32079679 10.1126/science.aay9187

[59] Santanello, J. A. et al. Land–atmosphere interactions: the LoCo perspective. *Bull. Am. Meteorol. Soc.* **99**, 1253–1272 (2018).

[60] Hersbach, H. et al. The ERA5 global reanalysis. *Q. J. R. Meteorol. Soc.* **146**, 1999–2049 (2020).

[61] Gelaro, R. et al. The modern-era retrospective analysis for research and applications, version 2 (MERRA-2). *J. Clim.* **30**, 5419–5454 (2017). 10.1175/JCLI-D-16-0758.1 PMC6999672 32020988

[62] Adler, R. F. et al. The version 2 global precipitation climatology project (GPCP) monthly precipitation analysis (1979–present). *J. Hydrometeorol.* **4**, 1147–1167 (2003).

[63] Schneider, U. et al. GPCC’s new land surface precipitation climatology based on quality-controlled in situ data and its role in quantifying the global water cycle. *Theor. Appl. Climatol.* **115**, 15–40 (2014).

[64] Huang, B. et al. Extended reconstructed sea surface temperature, version 5 (ERSSTv5): upgrades, validations, and intercomparisons. *J. Clim.* **30**, 8179–8205 (2017).

[65] Fan, Y., van den Dool, H. M. & Huang, B. Climate prediction center global monthly soil moisture data set at 0.5° × 0.5° resolution. *J. Geophys. Res.: Atmos.* **109**, D10102 (2004).

[66] Rodgers, K. B. et al. Ubiquity of human-induced changes in climate variability. *Earth Syst. Dynam.* **12**, 1393–1411 (2021).

[67] Eyring, V. et al. Overview of the coupled model intercomparison project phase 6 (CMIP6) experimental design and organization. *Geosci. Model Dev.* **9**, 1937–1958 (2016).

[68] Durre, I. et al. *Integrated Global Radiosonde Archive (IGRA), Version 2. NOAA National Centers for Environmental Information*, 10.7289/V5X63K0Q (2016).

[69] Willett, K. M., Dunn, R. J. H., Kennedy, J. J. & Berry, D. I. Development of the HadISDH marine humidity climate monitoring dataset. *Earth Syst. Sci. Data* **12**, 2853–2880 (2020).

[70] Johnston, B. R., Randel, W. J. & Sjoberg, J. P. Evaluation of tropospheric moisture characteristics among COSMIC-2, ERA5 and MERRA-2 in the tropics and subtropics. *Remote Sens.* **13**, 880 (2021).

[71] Elliott, W. P. & Gaffen, D. J. On the utility of radiosonde humidity archives for climate studies. *Bull. Am. Meteor. Soc.* **72**, 1507–1520 (1991).

[72] Kobayashi, S. et al. The JRA-55 reanalysis: general specifications and basic characteristics. *J. Meteor. Soc. Jpn.* **93**, 5–48 (2015).

[73] Holton, J. R. et al. *An Introduction to Dynamic Meteorology*, 4th edn. (Academic Press, 2004).

[74] Nigam, S., Chung, C. & DeWeaver, E. ENSO diabatic heating in ECMWF and NCEP reanalyses, and NCAR CCM3 simulation. *J. Clim.* **13**, 3152–3171 (2000).

[75] Roeckner, E. et al. The atmospheric general circulation model ECHAM5. Part I: Model description. MPI Rep. **349**, Max Planck Institute for Meteorology (2003).

[76] Seager, R. et al. Dynamical and thermodynamical causes of large-scale changes in the hydrological cycle over North America in response to global warming. *J. Clim.* **27**, 7921–7948 (2014).

[77] Wilks, D. S. “The stippling shows statistically significant grid points”: how research results are routinely overstated and overinterpreted, and what to do about It. *Bull. Am. Meteor. Soc.* **97**, 2263–2273 (2016).

[78] Ding, Q. et al. Regional drying over the Western U.S. driven by enhanced atmospheric subsidence amid global moistening from 1980 to 2020. diagnostic-of-Q-G-omega-equation. *Zenodo*10.5281/zenodo.19215627 (2026). 10.1038/s41467-026-71818-w PMC13265801 41991533

[79] Mayer, J., Mayer, M. & Haimberger, L. Consistency and homogeneity of atmospheric energy, moisture, and mass budgets in ERA5. *J. Clim.* **34**, 3955–3974 (2021).

