# Supplementary material for: Regional drying over the Western U.S. driven by enhanced atmospheric subsidence amid global moistening from 1980 to 2020
Source: Nat Commun. 2026 Apr 16;17:5270. doi: 10.1038/s41467-026-71818-w (PMC13265801; doi:10.1038/s41467-026-71818-w)
Supplement: Supplementary file 1 — Supplementary Information [file 41467_2026_71818_MOESM1_ESM.pdf]

Regional drying over the Western U.S. driven by enhanced atmospheric subsidence amid global moistening from 1980 to 2020

Qinghua Ding[1], Tiffany Shaw[2], Hailan Wang[3], Ian Baxter[2], Jiang Zhu[4]

1. Department of Geography, and Earth Research Institute, University of California, Santa Barbara, Santa Barbara, California, USA

2. Department of the Geophysical Science, The University of Chicago, Chicago, Illinois, USA

3. NOAA/Climate Prediction Center, College Park, Maryland, USA

4. NSF National Center for Atmospheric Research, Boulder, Colorado, USA

Corresponding author: Qinghua Ding, E-mail: qinghua@ucsb.edu

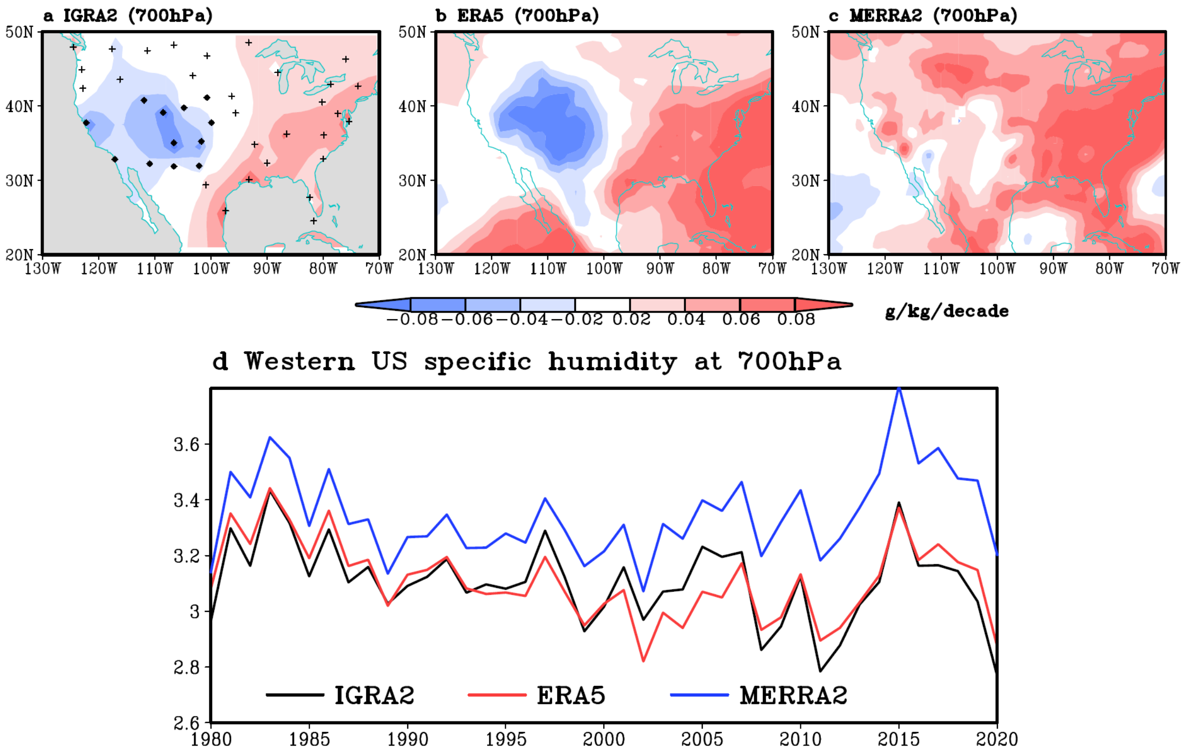

Supplementary Figure1: (a–c) Linear trends of annual mean specific humidity (g/kg/decade) at 700 hPa from IGRA2, ERA5, and MERRA2 over 1980–2020. IGRA2 station locations (41 total) are marked with crosses, and the 12 stations in the Western U.S. are additionally highlighted with closed circles. (d) Time series of annual mean specific humidity (g/kg) at 700 hPa averaged over the 12 Western U.S. stations (closed circles) from 1980 to 2020, based on IGRA2 (black), ERA5 (red), and MERRA2 (blue). The linear trend of the IGRA2 time series is −0.042 g/kg/decade, closely matching that of ERA5 (−0.045 g/kg/decade), with the IGRA2 trend being about 93% of ERA5. In contrast, MERRA2 shows a weak positive trend of +0.021 g/kg/decade. Both IGRA2 and ERA5 downward trends are statistically significant at the 95% confidence level. The correlation between the IGRA2 and ERA5 time series is 0.87, and between IGRA2 and MERRA2 is 0.76 over the full period.

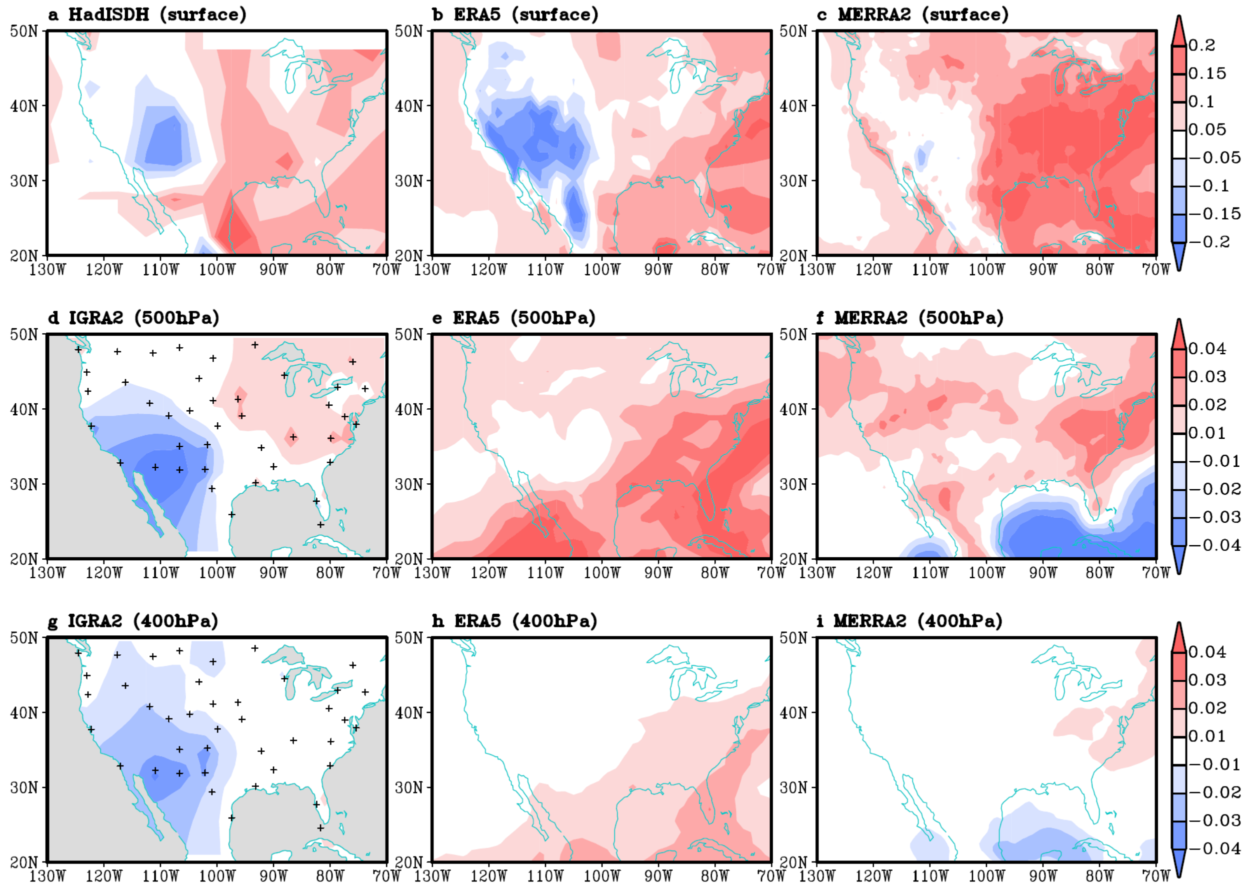

Supplementary Figure2: Linear trends of annual mean specific humidity at (a–c) surface (2 m), (d-f) 500 hPa, and (g-i) 400 hPa from HadISDH, IGRA2, ERA5, and MERRA2 over 1980–2020. IGRA2 station locations are marked with crosses. Note that the color scale for shading differs between the top row and the bottom two rows.

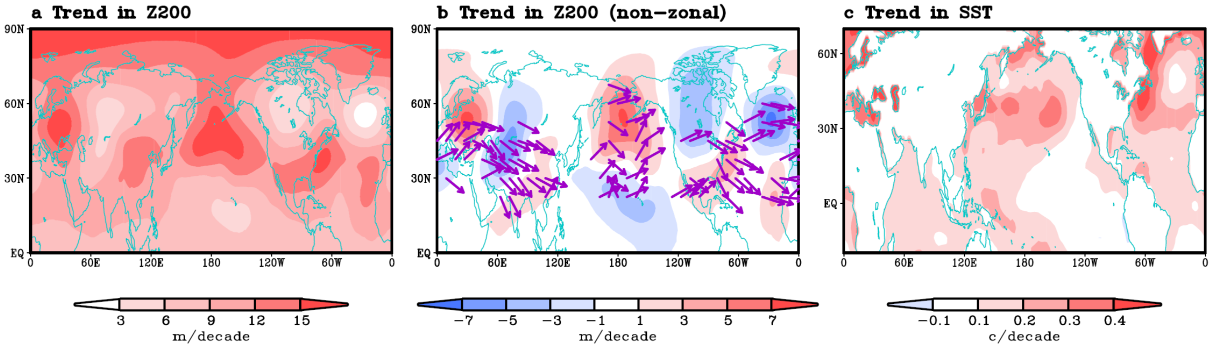

Supplementary Figure3: Linear trends of (a) annual mean 200 hPa geopotential height (Z200, m/decade), (b) the non-zonal component of the annual mean Z200 trend, and (c) annual mean SST from ERA5 (ERSST5 for SST) during 1980–2020. In (b), vectors indicate the Plumb (1985) wave activity associated with the non-zonal wave pattern of the annual mean Z200 trend.

Plumb, R. A., 1985: On the Three-Dimensional Propagation of Stationary Waves. J. Atmos. Sci., 42, 217–229.

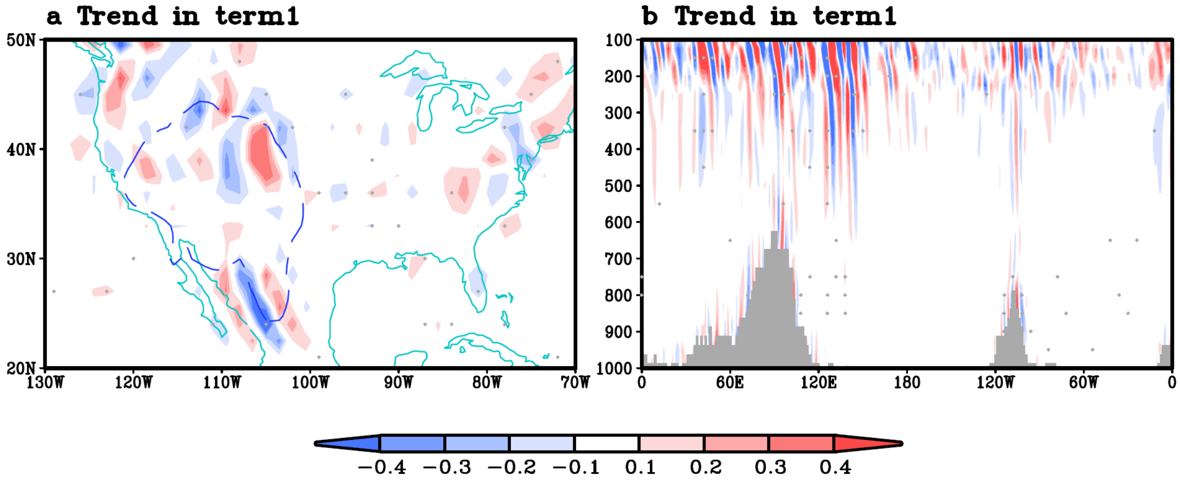

Supplementary Figure4: Linear trends of (a) annual mean vertical differential advection of geostrophic absolute vorticity by the geostrophic wind (term 1 in Eq.2, averaged between 300–800 hPa), where positive shading indicates a tendency to force downward motion, and (b) its corresponding trend patterns in zonal-vertical cross-sections from 1980 to 2020, derived from ERA5. The blue dashed contour indicates the region in the Western U.S., where the MH is most prominent during the period. Trends significant at the 95% confidence level are marked with dots.

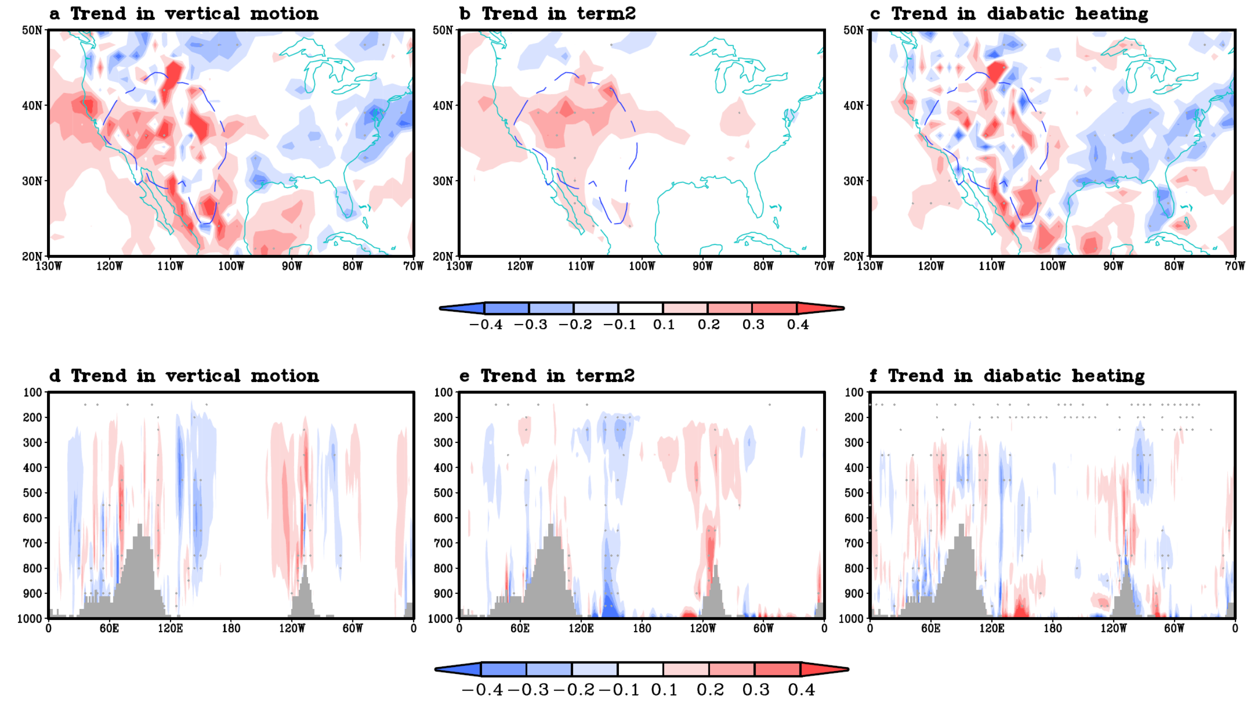

Supplementary Figure5: Linear trends of annual mean tropospheric (a) vertical motion (averaged between 300–800 hPa, scaled by 100 times, Pa/s/decade × 100), (b) horizontal temperature advection by the geostrophic wind, $V_g \cdot \nabla_p T$*, (term 2 in Eq.2, scaled by* $\frac{R_d}{\sigma p} \times 100$ *to match the units of vertical motion trends, averaged between 300–800 hPa), where positive shading indicates cold air advection and a tendency to force downward motion, (c) diabatic heating rate (sign reversed, -Q; term3 in Eq.2, scaled by* $\frac{R_d}{\sigma p} \times 100$ *to match the units of vertical motion trends, averaged between 300–800 hPa), where positive shading indicates diabatic cooling and a tendency to force downward motion, and (d to f) their corresponding trend patterns in zonal-vertical cross-sections from 1980 to 2020, derived from ERA5. The blue dashed contour indicates the region in the Western U.S., where the MH is most prominent during the period. Trends significant at the 95% confidence level are marked with dots.*

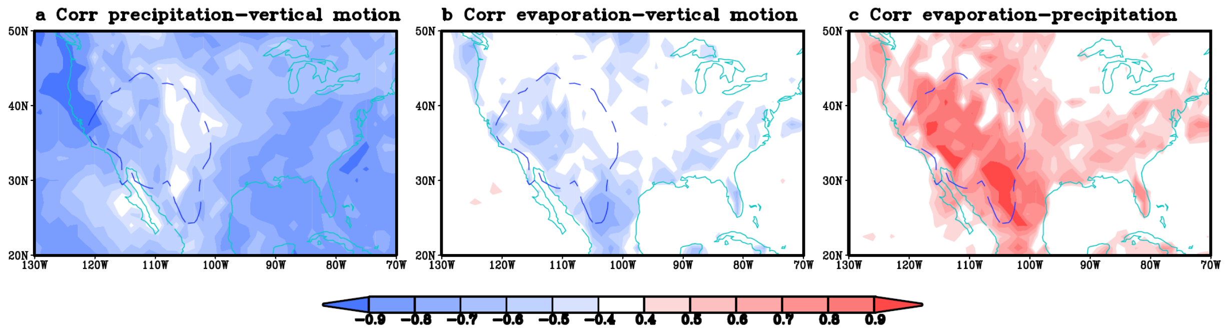

Supplementary Figure6: Grid-to-grid correlations of annual mean ERA5 fields (1980–2020): (a) precipitation vs. vertical motion, (b) evaporation vs. vertical motion, and (c) evaporation vs. precipitation. These results suggest a robust link between downward motion, reduced precipitation, and evaporation across the domain in ERA5. Since these variables that covary well locally may not be exactly co-located at each grid point, we account for this by calculating the correlation between the first variable (listed in each title) in each grid with the second variable within a 9-point neighborhood. In the plot, we display only those maximum values from this approach, which allows us to identify the strongest local correlation between the two variables at each grid point.

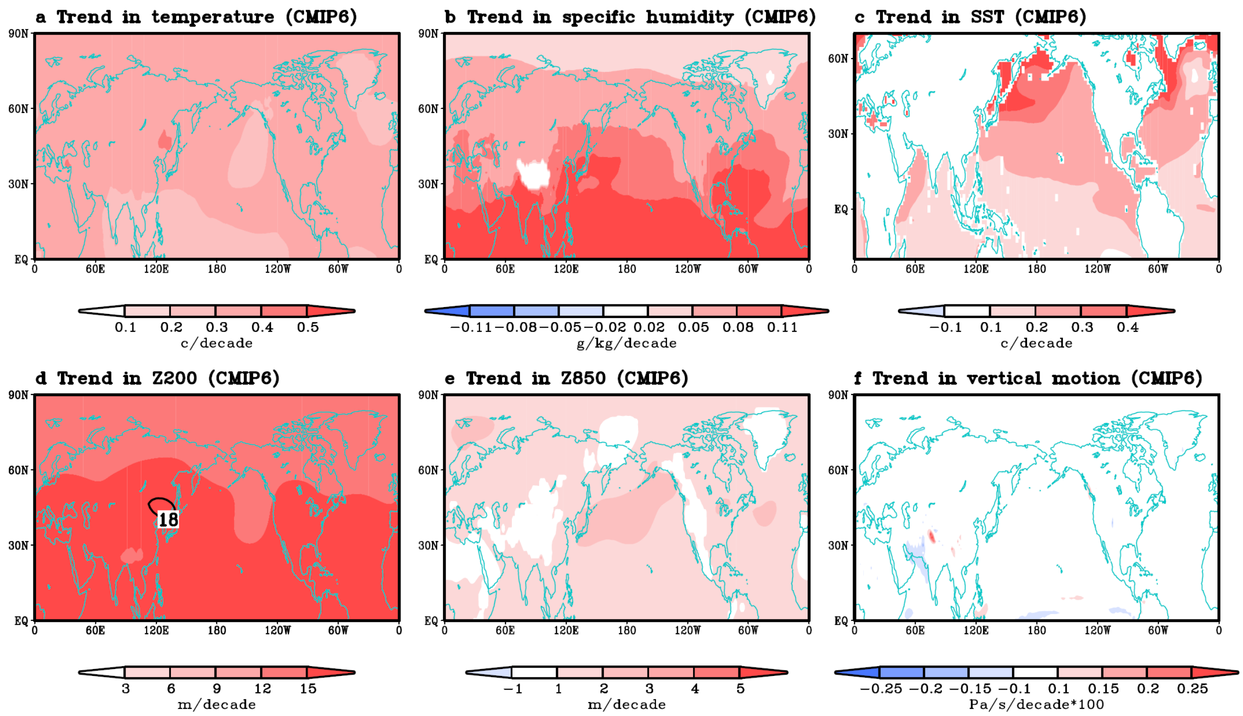

Supplementary Figure7: Same as Fig. 1 but derived from the ensemble means of 27 CMIP6 members. The color scale is adapted from that of Fig. 1 for easy comparison. In (d), trends in Z200 exceeding the maximum value of the color scale are shown in contours. From (a) to (e), all values are significant at the 95% confidence level. In (f), no values within the entire domain are significant at the 95% confidence level.

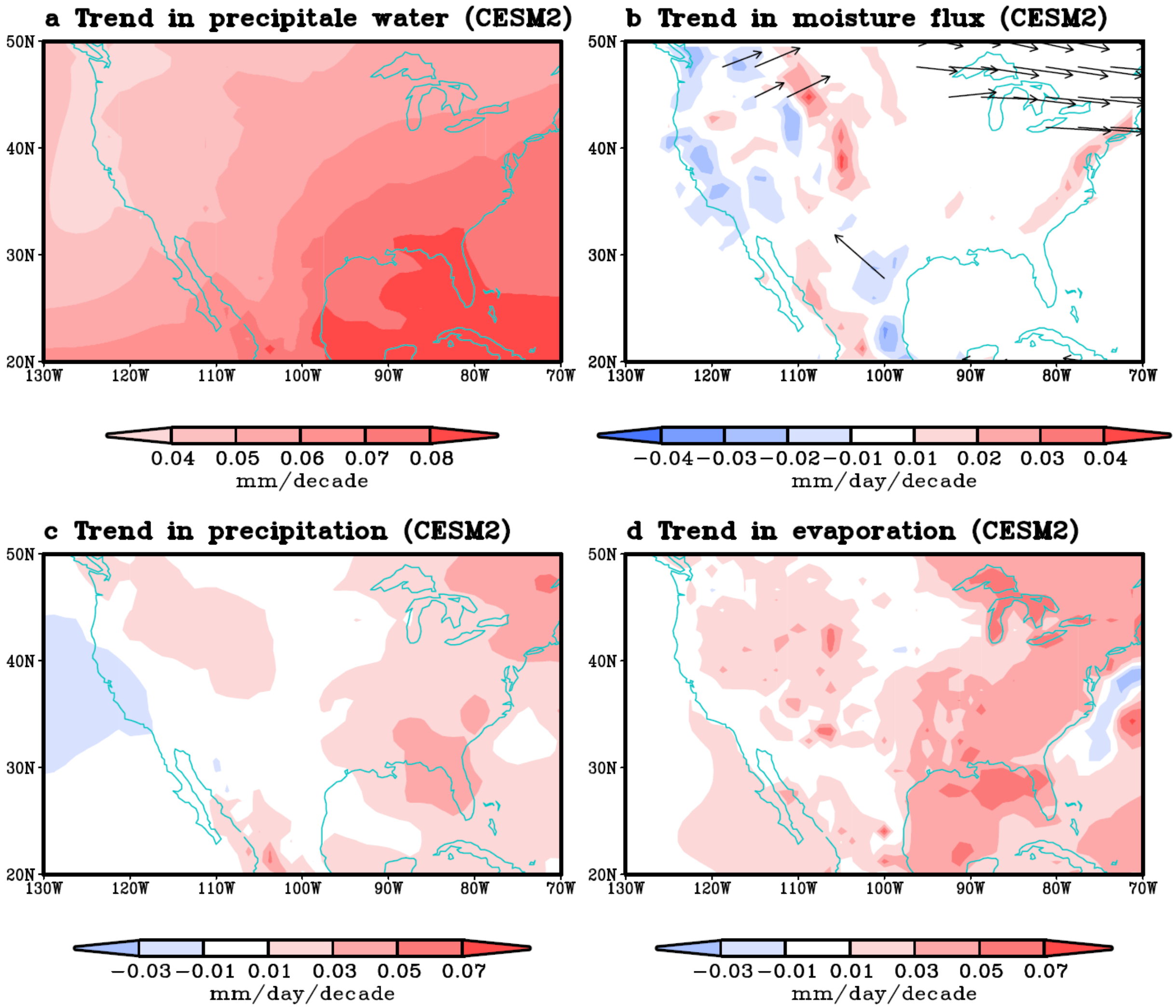

Supplementary Figure8: Same as Fig. 3. but derived from the ensemble means of 100 members of CESM2-LE.

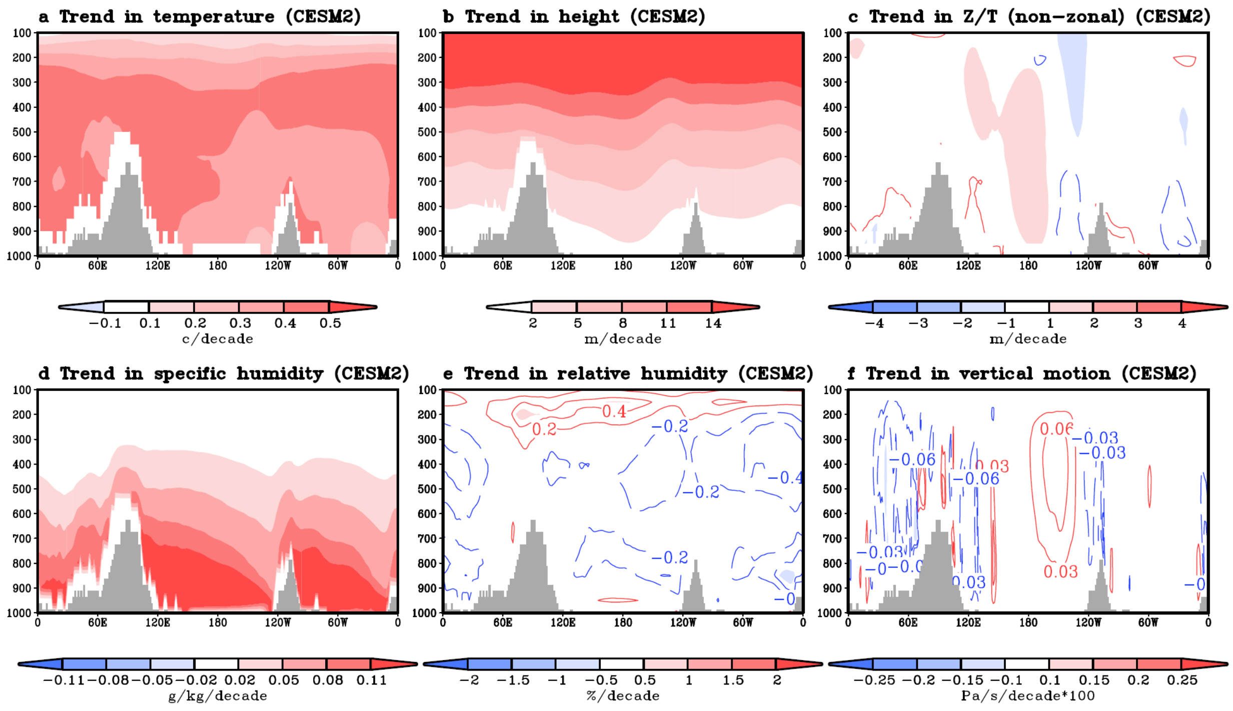

Supplementary Figure9: Same as Fig. 2 but derived from the ensemble means of 100 CESM2-LE members. The color scale is adapted from that of Fig. 2 for easy comparison. In (e) and (f), trends below the lowest value of the color scale are shown in contours. In (a), (b), and (d), all shaded values are significant at the 95% confidence level. In (e) and (f), no values are significant at the 95% confidence level.

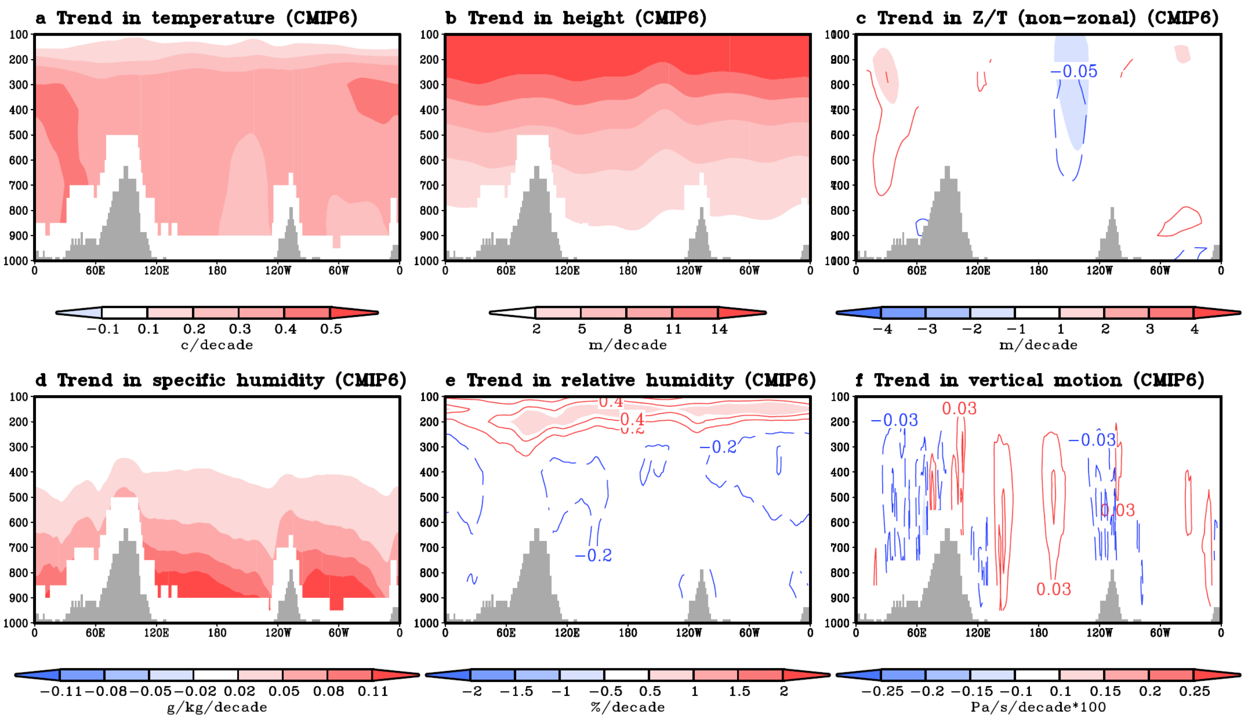

Supplementary Figure10: Same as Fig. 2 but derived from the ensemble means of 27 CMIP6 members. The color scale is adapted from that of Fig. 2 for easy comparison. In (e) and (f), trends below the lowest value of the color scale are shown in contours. In (a), (b), and (d), all shaded values are significant at the 95% confidence level. In (e) and (f), no values are significant at the 95% confidence level.

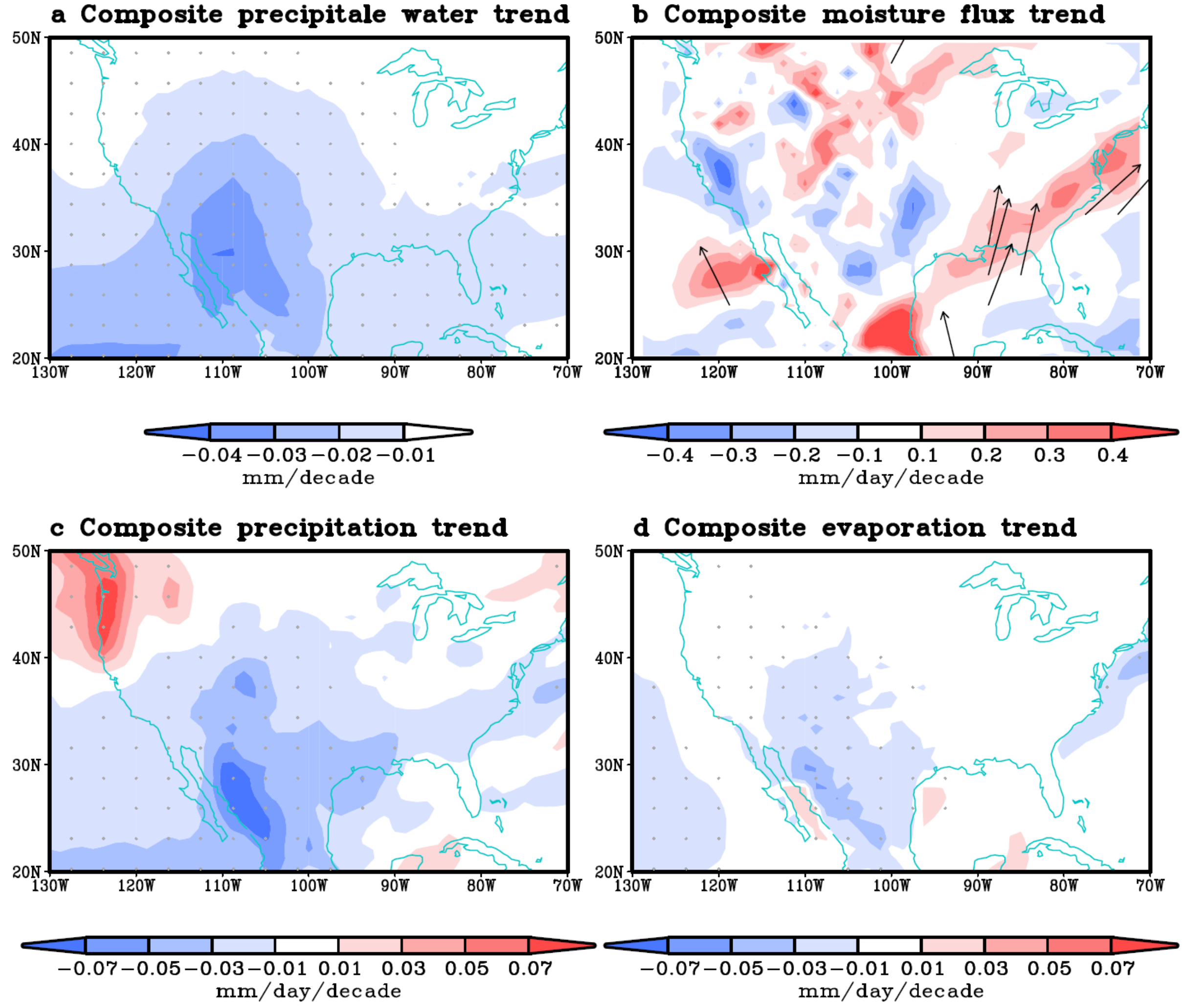

Supplementary Figure11: Same as Fig. 6, but for differences in composite linear annual mean trends of: (a) precipitable water, (b) IVT/IVT divergence (positive shading indicates a convergence of atmospheric moisture), (c) precipitation, and (d) evaporation. The differences between the two groups that are significant at the 95% confidence level are indicated by dots, based on a two-sample t-test.

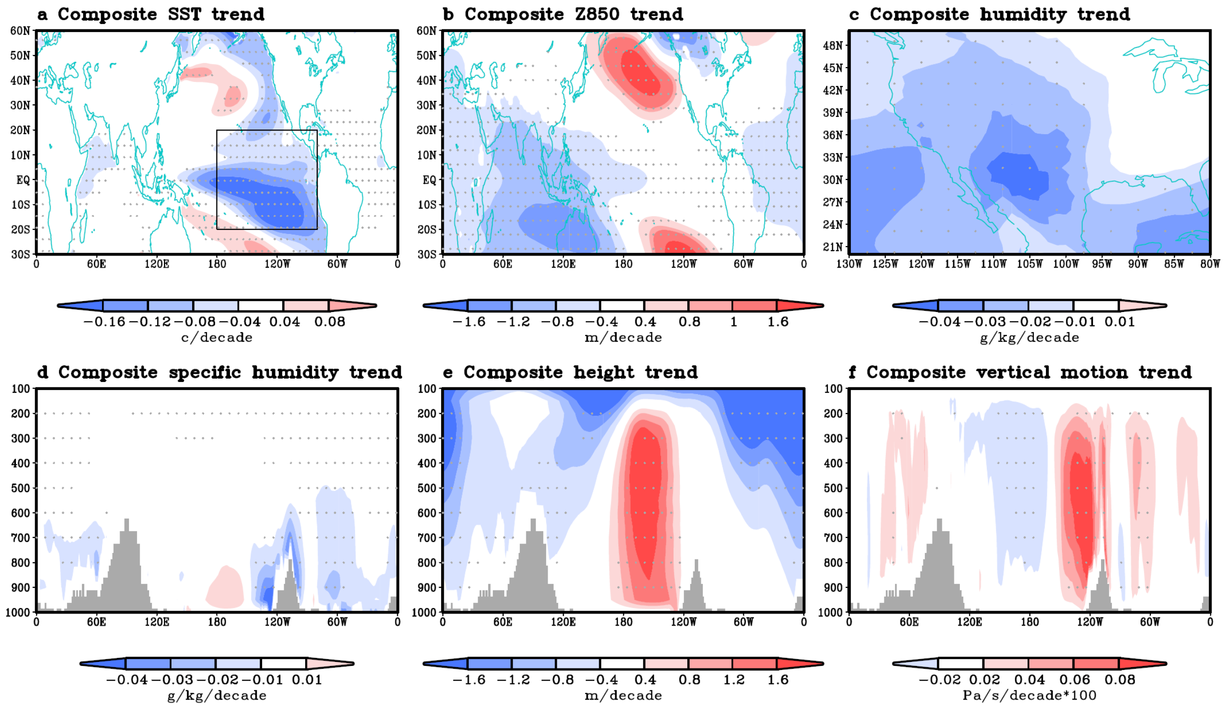

Supplementary Figure12: Differences in composite linear annual-mean trends between the 15 coldest and 15 warmest SST-trend members of CESM2-LE for (a) SST, (b) Z200, (c) lower-tropospheric specific humidity (650-1000 hPa), and meridional means (28°–42°) of (d) specific humidity, (e) geopotential height, and (f) vertical motion. Dots indicate differences significant at the 95% confidence level (two-sample t-test). The composite members are selected based on the annual-mean SST trend (1980-2020) averaged within the box in (a) for each member.

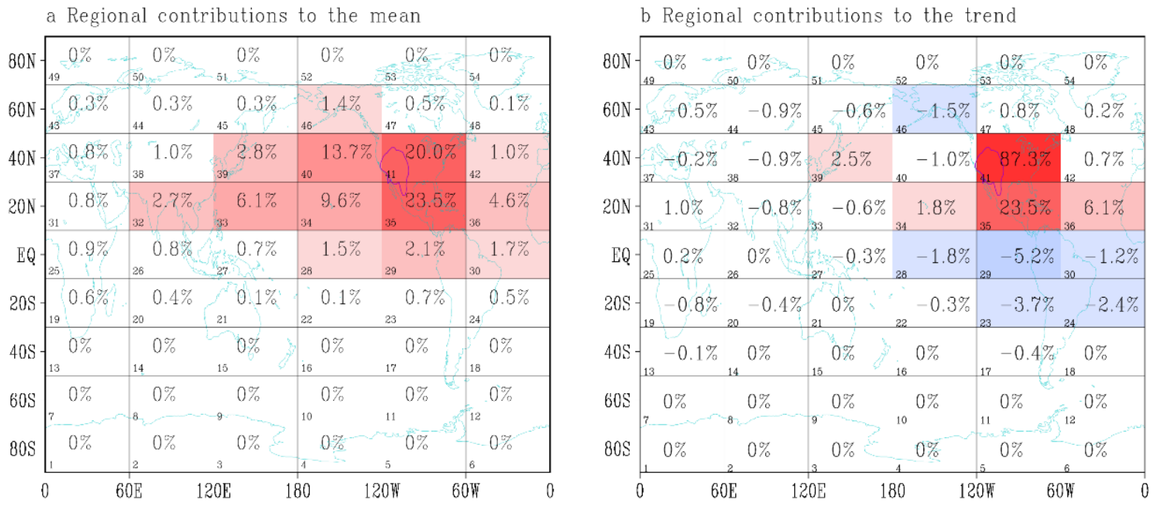

Supplementary Figure13: Fraction of (a) the climatological mean and (b) the trend of annual mean lower-tropospheric (averaged between 650-1000 hPa) specific humidity over the Western U.S. (averaged within the purple contour) from 1980 to 2020, contributed by each tagging region in the global tagging experiment. For each tagging region, the number in the lower-left corner indicates its sequence from 1 to 54. The second percentage number indicates the contribution of each source region to (a) the climatological mean and (b) the linear trend from 1980 to 2020. Darker colors indicate higher contributions. Red indicates a positive contribution (>1%) to the mean or trend, while blue (<–1%) indicates a negative contribution to the atmospheric drying trend over the Western U.S. (contributing to a wetting trend). Panel (b) is the same as Fig. 8a but shown at a larger spatial scale.

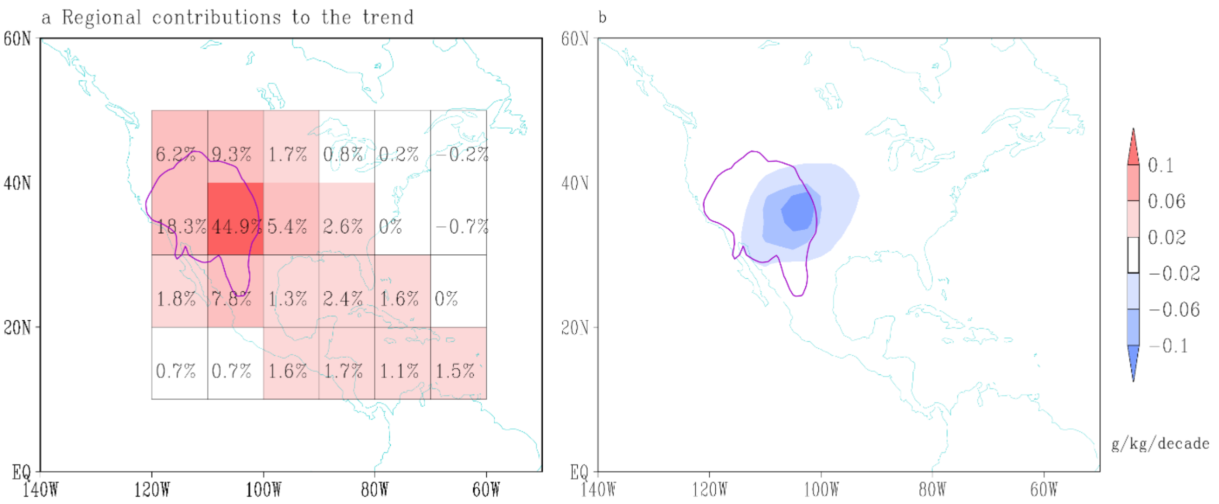

Supplementary Figure14: (a) Same as Supplementary Fig. 13, but for the fraction of the linear annual mean trend of lower-tropospheric (averaged between 650 to 1000 hPa) specific humidity over the West, from 1980 to 2020, contributed by each smaller tagging region in the regional tagging experiment. (b) Same as Fig. 8e but for the trend from the reddest region near New Mexico (contributing 45% of the trend in (a)) in the regional tagging experiment.

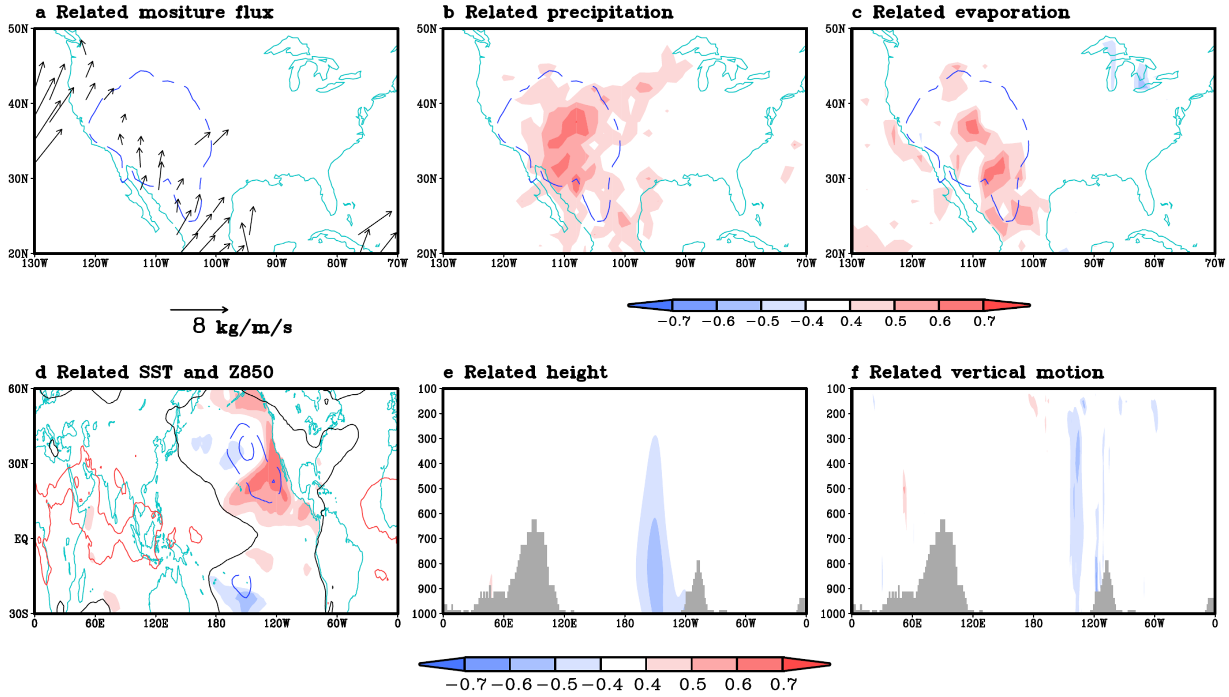

Supplementary Figure15: Correlations of detrended annual mean lower-tropospheric (averaged between 650–1000 hPa) specific humidity, averaged within the blue contour (the region with the prominent MH feature), with detrended (a) annual mean IVT (calculated using hourly data[53], arrows showing statistically significant regressed values, unit: kg/m/s), (b) annual mean precipitation, (c) annual mean evaporation, (d) annual mean SST (shading) and Z850 (contours), and meridional averages (averaged between 28°–42°) of annual mean (e) height and (f) vertical motion, based on ERA5 data from 1980 to 2020.

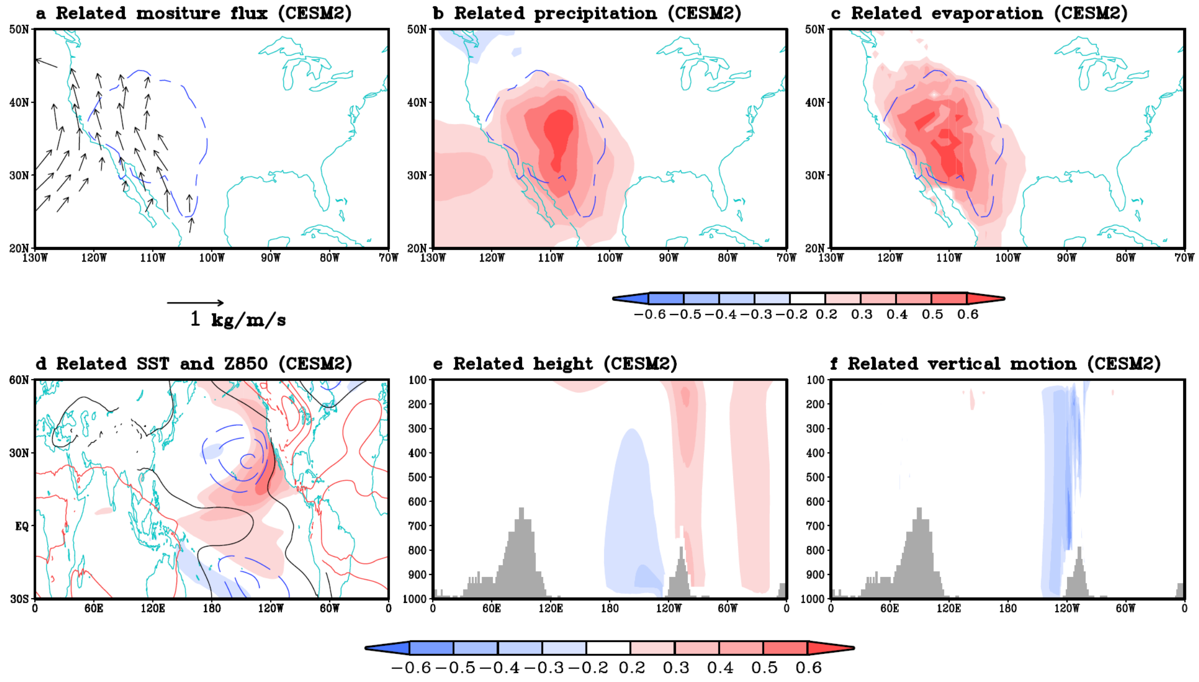

Supplementary Figure16: Same as Supplementary Fig. 15, but based on 100 members of CESM2-LE. Annual mean fields from 1980 to 2020 in each ensemble member are detrended before calculating the correlations. The correlation (or regression for (a)) patterns are then averaged across all 100 members.

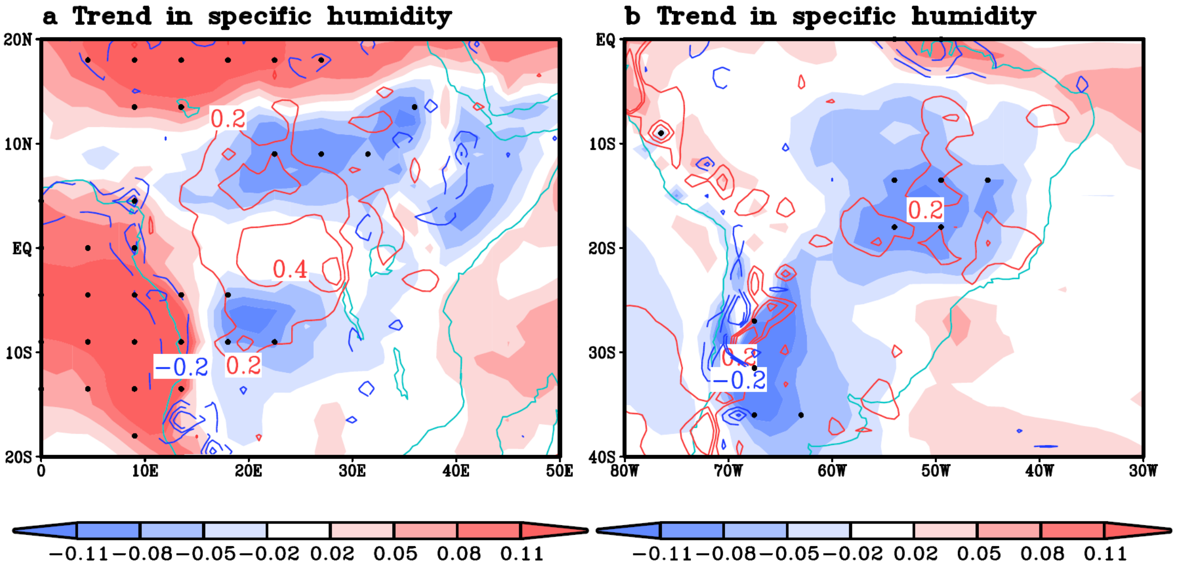

Supplementary Figure17: Linear trends of annual-mean specific humidity (averaged between 650–1000 hPa, g/kg/decade, shading) and tropospheric vertical motion (averaged between 300–800 hPa, contours) over (a) Central Africa and (b) the Amazon from ERA5 during 1980–2020. Trends in specific humidity significant at the 95% confidence level are marked with dots.

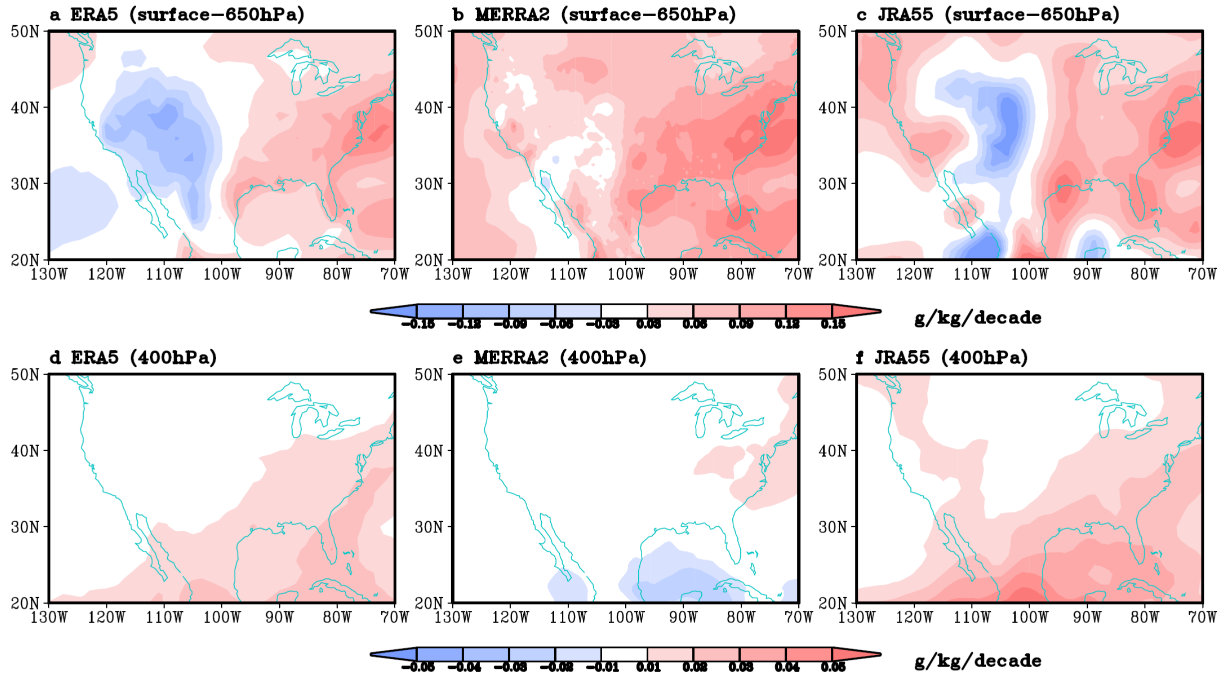

Supplementary Figure18: Linear trends of annual mean specific humidity from 1980–2020: (upper panel) averaged between 650–1000 hPa (g/kg/decade) and (lower panel) at 400 hPa (g/kg/decade), from ERA5, MERRA2, and JRA55, respectively.

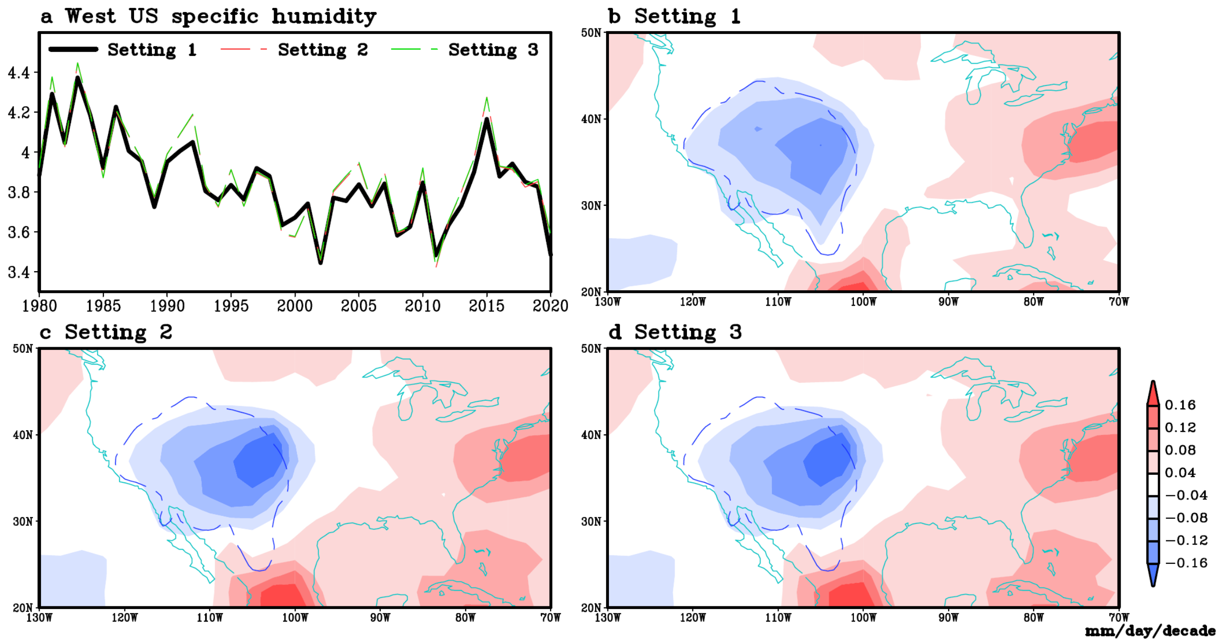

Supplementary Figure19: (a) Time series of annual mean lower-tropospheric (averaged between 650 to 1000 hPa) specific humidity averaged over the Western U.S. (within the dashed blue contour in (b)) from 1980 to 2020 in our nudging experiments under three different settings (see Methods), along with the spatial patterns of linear trends over 1980-2020 in annual mean lower-tropospheric specific humidity.

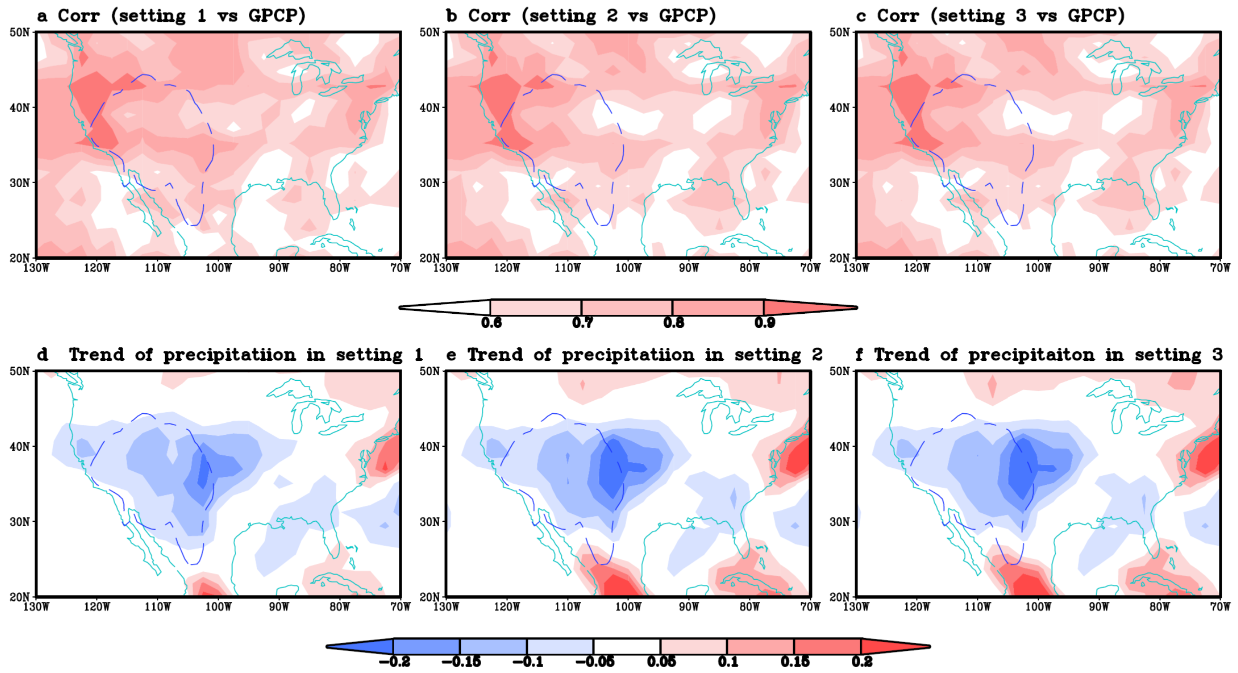

Supplementary Figure20: (a to c) Grid-to-grid correlations of annual mean GPCP precipitation at each grid point with annual mean precipitation simulated by our nudging experiments under each setting from 1980 to 2020, along with (d to f) the spatial patterns of linear trends in annual mean precipitation (mm/day/decade) simulated in our nudging runs under each setting.

.

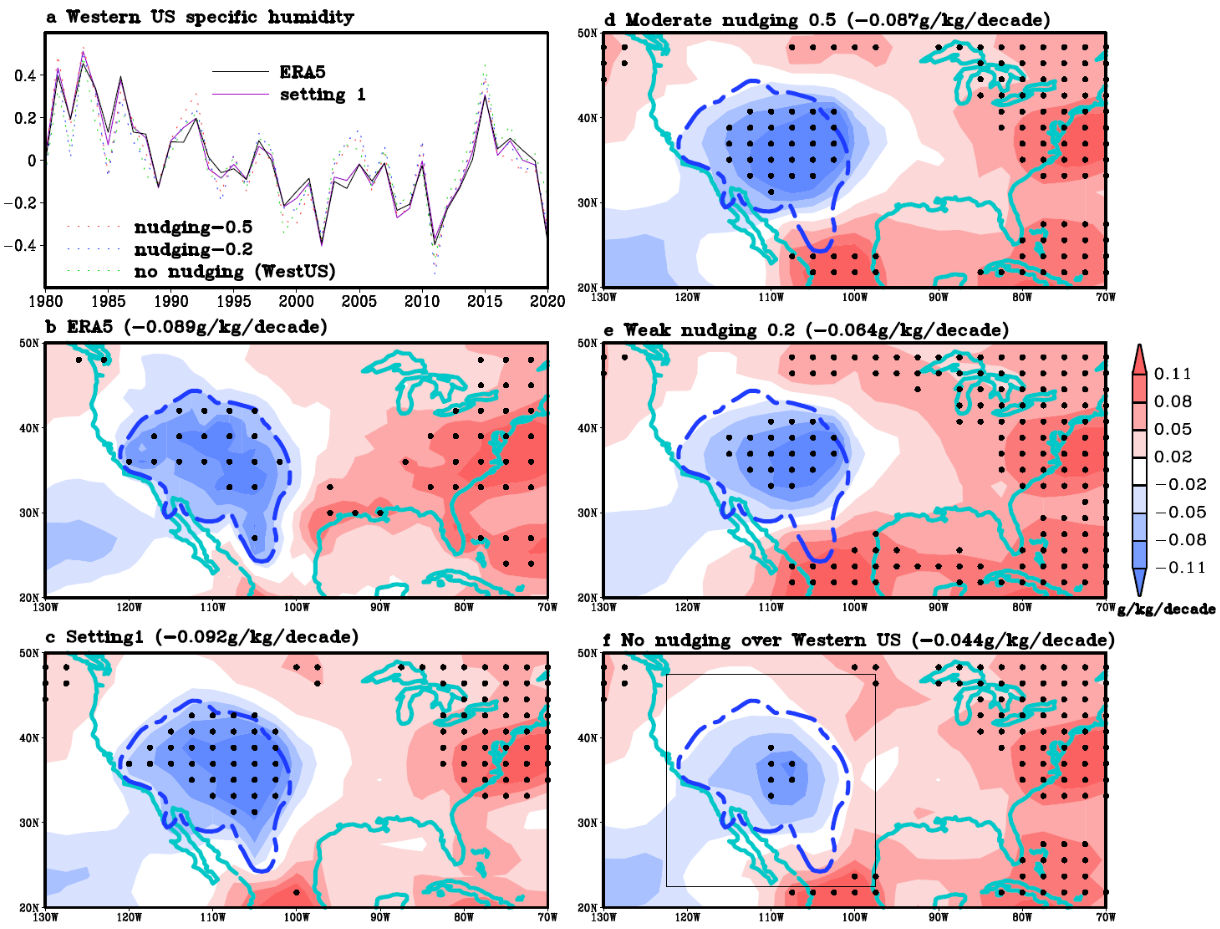

Supplementary Figure21: (a) Time series of annual-mean lower-tropospheric specific humidity (averaged from 650 to 1000 hPa) over the Western U.S. (within the dashed blue contour in (b) from 1980 to 2020, shown for ERA5, the global tagging experiment (Setting 1), and three additional tests (see Methods). The magnitude of the MH trend is indicated in each title. (b–f) Spatial patterns of linear trends in annual-mean lower-tropospheric specific humidity: (b) ERA5, (c) the global tagging experiment (Setting 1), and (d–f) the three new tests. Trends significant at the 95% confidence level are marked with dots. The box in (f) denotes the region where no nudging was applied.

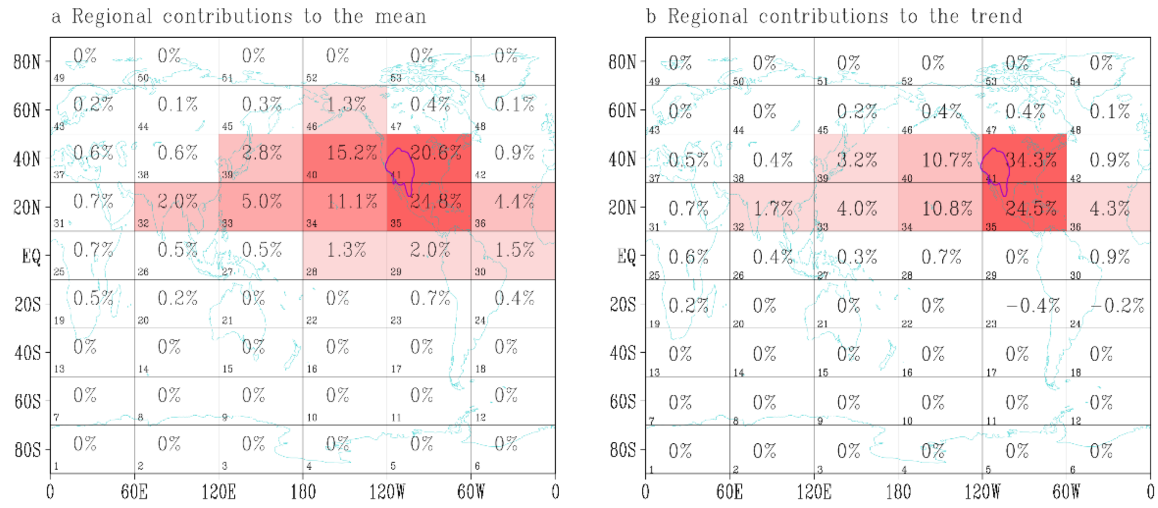

Supplementary Figure22: Same as Supplementary Fig. 13, but for the fraction of (a) the climatological mean and (b) the linear trend of annual mean precipitation over the Western U.S. (averaged within the purple contour) from 1980 to 2020, contributed by each tagging region in the global tagging experiment.

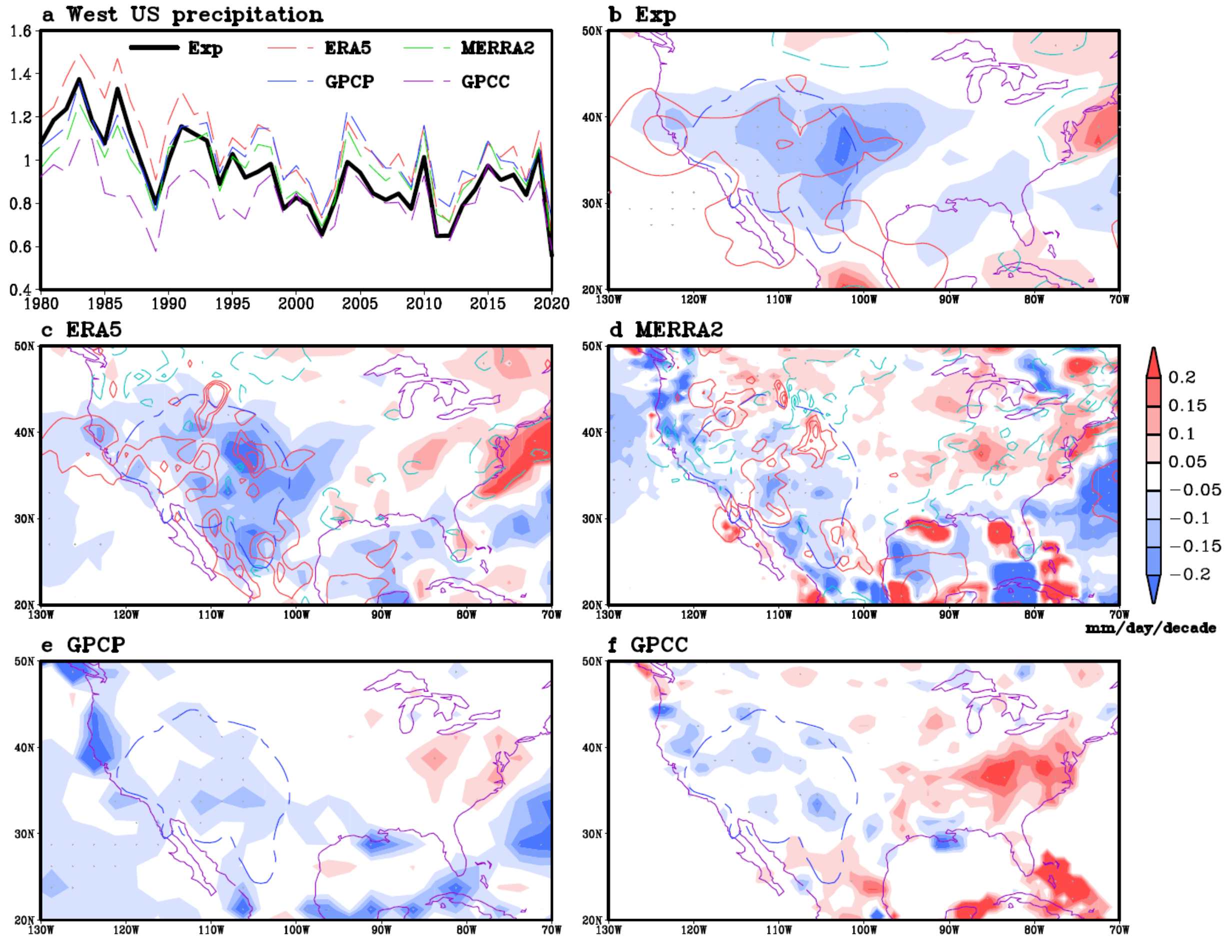

Supplementary Figure23: (a) Time series of annual mean precipitation averaged over the Western U.S. (blue dashed contour in (b)) from 1980 to 2020 in ERA5 (red), MERRA2 (green), GPCP (blue), GPCC (purple), and our global nudging experiment (black), along with spatial patterns (b–f) of their linear trends over 1980–2020. Contours in panels (b–d) indicate the linear trend of annual mean tropospheric vertical motion (averaged between 300 to 800 hPa, positive values indicate downward motion) from the corresponding dataset.

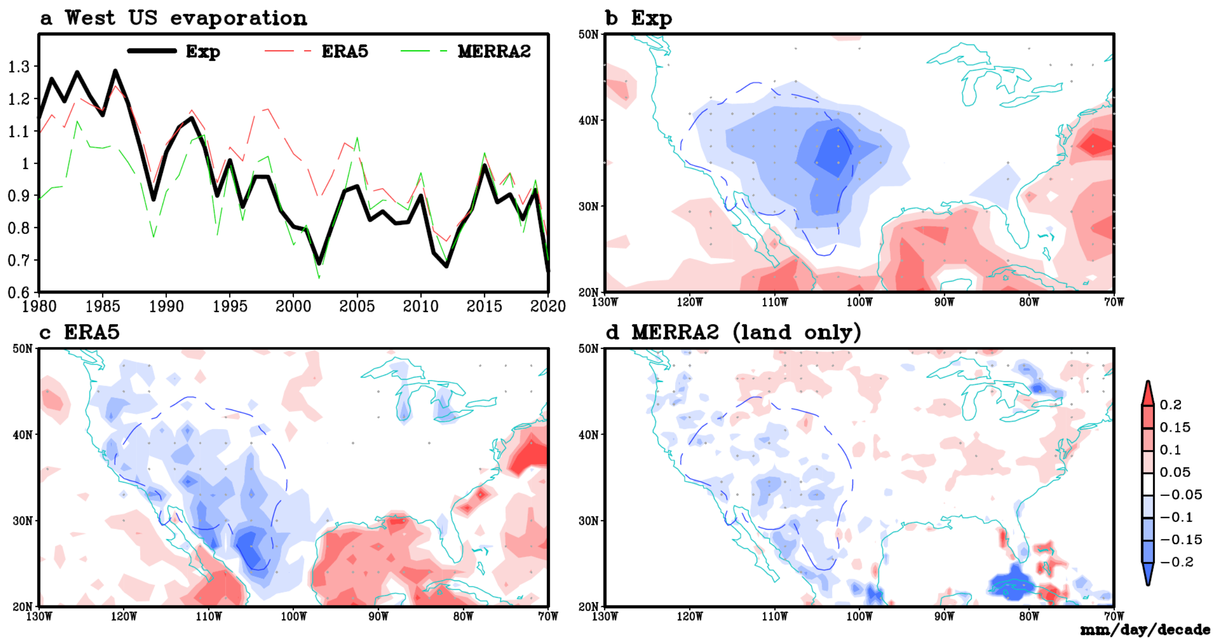

Supplementary Figure24: (a) Time series of annual mean evaporation averaged over the Western U.S. from 1980 to 2020 (blue dashed contour in (b)) from ERA5 (red), MERRA2 (green), and our global nudging experiment (black), along with spatial patterns (b–d) of their linear trends over 1980–2020.

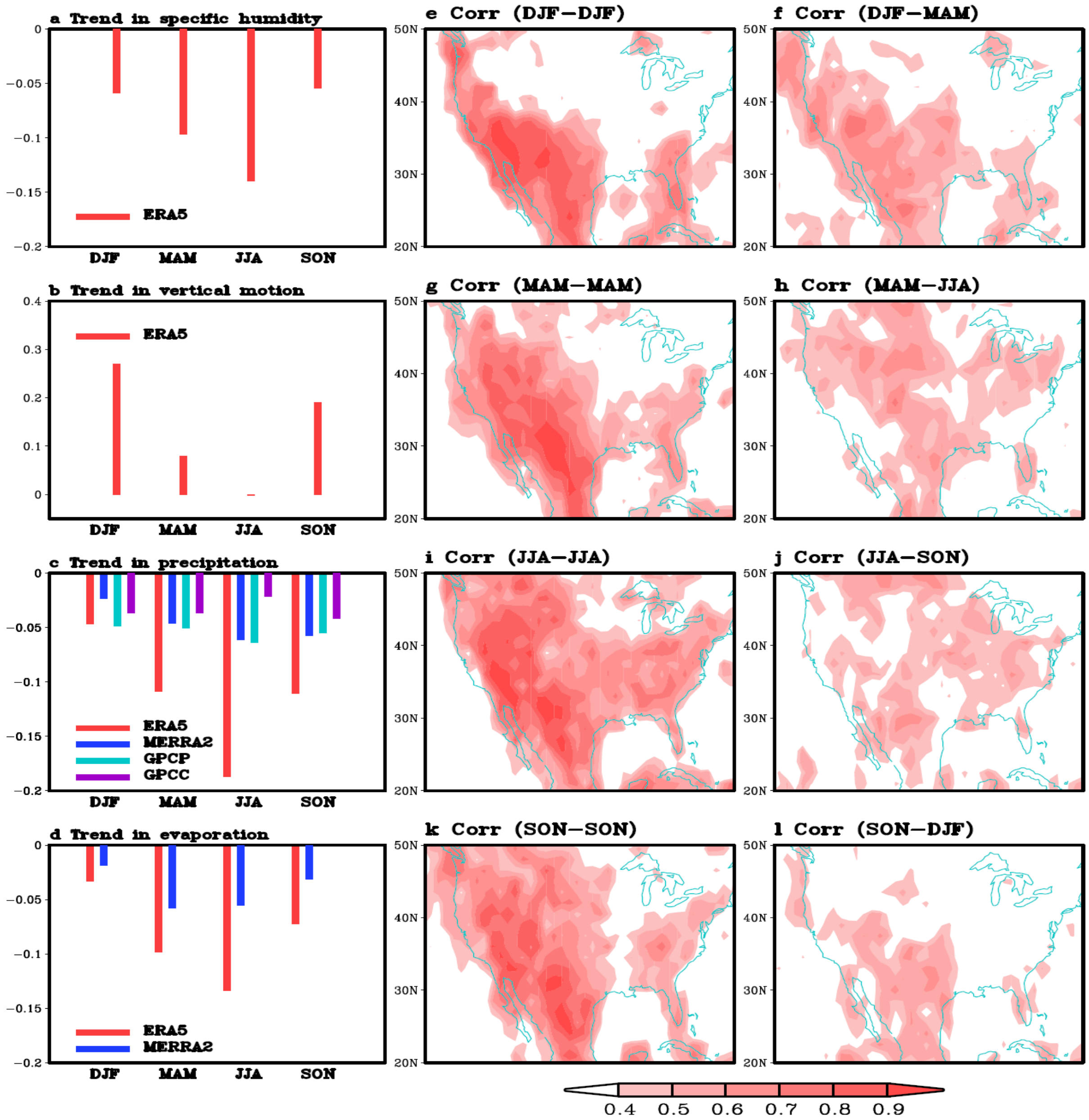

Supplementary Figure25: (a to d) Linear trends of key variables within the MH region for each season from ERA5 during 1980–2020. For precipitation and evaporation, multiple datasets are used to highlight the diverse trends across different sources. In the middle and right columns, grid-to-grid simultaneous correlations of precipitation and evaporation, as well as cross-seasonal correlations (precipitation leading evaporation by one season), are shown for each season. Because variables that covary locally may not be exactly co-located at each grid point, we calculate the correlation between precipitation at each grid point and evaporation within a 9-point neighborhood. In each grid, we display only the maximum values from this approach, which allows us to identify the strongest simultaneous or lead–lag correlation between the two variables at each grid point.

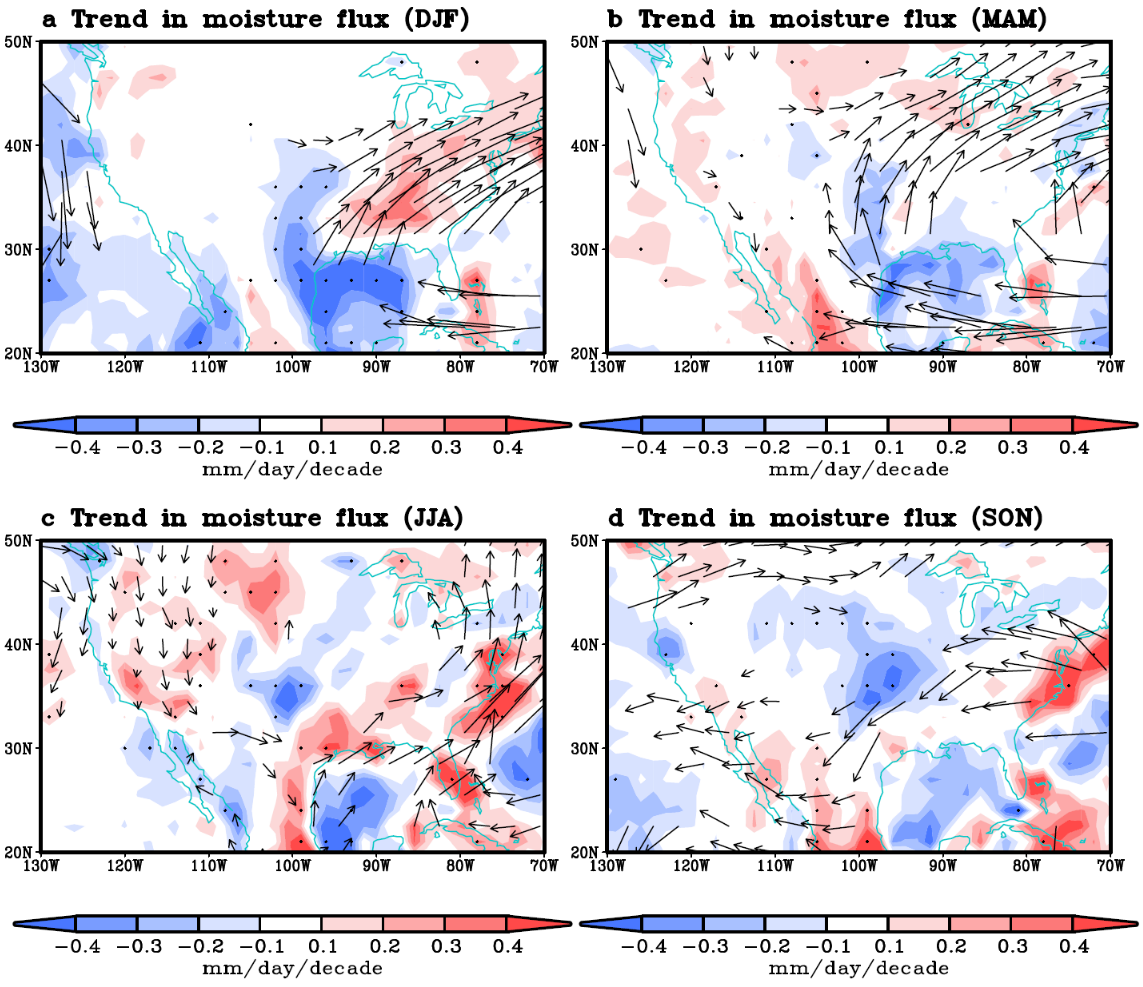

Supplementary Figure26: Linear trends of IVT and its divergence (mm/day/decade, positive shading indicates a convergence of atmospheric moisture) in each season from ERA5 during 1980–2020. Significant trends above the 95% confidence level are marked with dots.
