# Supplementary material for: Regional drying over the Western U.S. driven by enhanced atmospheric subsidence amid global moistening from 1980 to 2020
Source: Nat Commun. 2026 Apr 16;17:5270. doi: 10.1038/s41467-026-71818-w (PMC13265801; doi:10.1038/s41467-026-71818-w)
Supplement: Supplementary file 2 — Transparent Peer Review file [file 41467_2026_71818_MOESM2_ESM.pdf]

nature portfolio

Peer Review File

Regional drying over the Western U.S. driven by enhanced atmospheric subsidence amid global moistening from 1980 to 2020

Corresponding Author: Professor Qinghua Ding

This manuscript has been previously reviewed at another journal. This document only contains information relating to versions considered at Nature Communications. Mentions of the other journal have been redacted.

This file contains all reviewer reports in order by version, followed by all author rebuttals in order by version.

Version 0:

Reviewer comments:

Reviewer #1

(Remarks to the Author)
This study investigates the cause of the moistening hole–like change over the Western U.S. in the context of global warming. By analyzing a wide array of observational data, CMIP6 runs, CESM2-LE experiment, sea surface temperature pacemaker runs, and water-tagging enabled simulations, this article reveals that sea surface temperature cooling in the tropical eastern Pacific can induce a barotropic high-pressure circulation trend over the North Pacific, which in turn enhances atmospheric subsidence and reduces atmospheric moisture over the region. The conclusions drawn from the analysis are generally sound and suggest important implications. The story in this paper isn't particularly groundbreaking but is interesting.
The revision is clearer and better organized, and the authors have largely provided clear answers to the reviewers' questions. It is potentially publishable after the authors address several additional questions and comments listed below.

Major comments:
Although the difference between the PAC results and CESM2-LE ensemble mean indicates a drying trend over the western U.S., the tropical Pacific SST anomaly in this case represents the difference between the observed SST trend and an El Niño-like SST anomaly. Therefore, I am uncertain about the magnitude and significance of the observed SST changes' specific impact and contribution, as well as the degree of SST nonlinearity. Since the authors have already performed numerous experiments, there is no need for additional simulations. However, it would be helpful if the authors could clarify how they understand and explain this result. For example, could the authors further show the differences in composite linear annual-mean trends between the 15 coldest and the 15 weakest SST-trend members of CESM2-LE, as well as between the 15 warmest and the 15 weakest SST-trend members, in addition to Extended Data Fig. 13 ?

Minor comments:
1. Line 49, please change "represent" in "represents".
2. Lines 50-51, The word "droughts" in the original sentence is somewhat repetitive; consider revising the sentence "contributing to prolonged meteorological droughts and related droughts in the agricultural, hydrological, and socioeconomic sectors of the region" to "contributing to prolonged droughts across the meteorological, agricultural, hydrological, and socioeconomic sectors of the region".
3. Line 72-73: You could mention this paper about the impact of the West Pacific (WP) teleconnection. https://agupubs.onlinelibrary.wiley.com/doi/full/10.1029/2024EF004922
4. Line 86, please change "forcing" in "forcings".
5. Line 110. It seems that there is also a significant decrease in specific humidity over central-eastern Africa, north of the equator.
6. Lines 165-169. I agree that reduced evaporation may play a more direct role in atmospheric drying, but why does the decreasing trend in precipitation appear slightly stronger than that of evaporation over the western U.S.?
7. Lines 238-240. Circulation and SST trend patterns between the 15 coldest and 15 warmest SST-trend members of CESM2-LE are shown in Extended Data Fig. 13, rather than Extended Data Fig. 12. Please revise it.

8. Line 253, please change “dying” in “drying”.
9. Lines 266-269. The PAC run fails to reproduce the observed vertical motion trend over the central and eastern U.S. The authors attribute this mainly to the regions being relatively distant from the SST forcing. I think it might be helpful to briefly mention that the ERA5 trend in this region could also be influenced by other factors, such as Arctic sea-ice loss or other remote forcings.
10. Lines 320-321. Does the annual mean IVT also show weak trends over these two regions?
11. Line 581, please change “horizonal” in “horizontal”.
12. Line 737, please change “precious” in ”previous”.
13. References 13 and 77 refer to the same article. Please verify this and make the appropriate correction.

(Remarks on code availability)
I briefly reviewed the provided code and did not find any issues. However, I did not attempt to install or run the code.

Reviewer #2

(Remarks to the Author)
Review of "Regional drying over the Western U.S. driven by enhanced atmospheric subsidence amid global moistening from 1980 to 2020"

I thank the authors for their responses to my comments as Reviewer 1 from the [*REDACTED*] review process. I believe the revised work is interesting and important, but that it would be better placed in a long-form journal like Journal of Climate, within which the methodological choices could be discussed alongside with the results. In my mind, the primary contributions of the work are illuminating the dynamical links from tropical Pacific SSTs to the midlatitudes, as well as (potentially, see comments below) the results from the water tagging experiments. These will be of great interest to a climate audience if the paper is written such that the methods and results are more transparent and clear.

Major comments

- There are two large comments from my prior review that I do not believe were addressed. The first is about the combination of water tagging and nudging. Based on the prior response, my best guess is that, when humidity is added at a given point through the increments, that the humidity is "sourced" from that location because the authors seem to be interpreting the nudging as reflecting a local source. If this is correct (and it may not be, but my question was not fully answered before), then I am skeptical of the water tagging results. We know that CESM "wants" to get more humid. The increments will presumably reduce humidity in the US Southwest. If these increments are viewed as local sources, then it will appear that the local sources are reducing over time. This needs to be clarified in the paper. The authors also did not answer my question about the similarities between Settings 2 and 3, which I believed to be surprising given the likely strong physiological plant response to CO2 in the model.

- I appreciate the addition of the experiment where the nudging is only outside the western US. However, the authors large brush over the fact that the humidity response is much smaller (i.e. the trend less negative) in that simulation. This suggests that the large-scale forcing may not be playing as much of a role as has been argued elsewhere. It would be great to see further discussion of this in a longer-form paper.

- In their focus on the role of the tropical Pacific, the authors compare PAC minus CESM2 to the observations, which is confusing. As they note, PAC minus CESM2 (ideally) isolates the role of tropical Pacific SST forcing. But the observations result from both tropical Pacific SST forcing and the ongoing anthropogenic forcing, and the latter is removed when subtracted CESM2 from PAC. I think the text needs to be reworded to note that the tropical Pacific SST forcing induces a drying over the western US, but that the PAC ensemble as a whole still has positive trends in specific humidity everywhere. This then suggests that the damped / lack of warming in the eastern Pacific is playing a role in the lack of humidity increase, but it is not the whole story, because when anthropogenic forcing is included (in PAC), the region still moistens.

- Regarding code availability, the authors wrote: "Other analyses (e.g., composites, trend analysis, correlation calculations, and two-sample tests) are standard and therefore not included in the repository." I strongly suggest (and Nature Comms may require?) that all code is made available. I have experienced many times confusing wording in a manuscript in terms of methods that can be immediately clarified in code. Further, other researchers cannot easily build off your code and results if many of the relevant pieces are missing.

- Please improve the quality of the figures for clarity. The colorbars remain very coarse, and I don't see a reason to use a different number of color blocks per figure. Using 8-10 is a reasonable balance between providing interpretable figures and not getting too close to a continuous colorbar. In the line plots, the lines are very thin, and it is often difficult to even see the dashed or dotted lines. The figures all seem to be have been saved at low resolution.

Line-specific comments
- L64: "Alternatively... limited attention in previous research focused on this region." There is an enormous amount of research linking Pacific variability to hydroclimate in the US SW.
- L104: LOWER troposphere
- L118: Incorrect figure reference
- L193: Many comparisons of the observations to the ensemble mean have been modified, but some remain, including in this

paragraph.
- L229: Suggest different word than "fingerprint" which is typically associated with D&A/optimal fingerprinting. This is a composite analysis.
- L244: The composite analysis does not provide evidence that tropical Pacific SSTs "drive" the MH, but rather that they are related. The compositing on Pacific SST is more convincing, but still cannot be used to suggest causality.
- L259: As mentioned in my prior review, the CESM2-LE has different forcings for the first and second 50 members in terms of the biomass burning.
- L516: Based on the prior review discussion, I do not believe that the IGRA data being used are homogenized. Given the known, large inhomogeneties in radiosonde humidity data, this should be discussed explicitly. Also, the difficulty of homogenizing radiosonde data should be noted, so ERA5 may also be affected by inhomogeneities.

(Remarks on code availability)
The code does not allow for full reproduction of the results (also see my comment in the main review), and is in Fortran, which few scientists use now.

Version 1:

Reviewer comments:

Reviewer #1

(Remarks to the Author)
Review of "Regional drying over the Western U.S. driven by enhanced atmospheric subsidence amid global moistening from 1980 to 2020"
After two rounds of revision, I find that this study, based on analyses of reanalysis datasets and multiple numerical simulations, offers a convincing explanation for the emergence of the moistening hole–like signal over the western United States. In particular, the use of iCESM with water tagging provides a novel methodological approach for hydroclimate research over the western United States. Now, I have only three minor comments.

1. In Resp. Fig. 1, could the authors clarify whether the units are correct? If the figure shows differences between 30-year mean states, these quantities should represent mean differences rather than time-dependent changes (e.g., per decade).
2. For the CAM5 and ECHAM5 experiments, how large is the magnitude of the North Pacific circulation response? In particular, how does it compare with the observed changes over the past 41 years? Quantifying how much of the observed strengthening of the North Pacific circulation can be attributed to the imposed tropical cooling would help clarify the contribution and impact of tropical SST forcing.
3. The authors should add the availability of the generated datasets used in this work (e.g., the AMIP experiments) in the Data Availability.

(Remarks on code availability)

Reviewer #3

(Remarks to the Author)
The authors analyze the characteristics and potential mechanisms behind the observed "Moistening Hole" (MS) that has characterized large portions of the western US over the period 1980-2020. Their central thesis is that long-term cooling over the tropical eastern Pacific drives an atmospheric teleconnection pattern consisting of ridging over the North Pacific and subsequent descending motion on the eastern flank of this ridge over the western US. They conduct dedicated numerical experiments to explore this, as well as diagnostics with a large ensemble of global climate model simulations and multiple reanalysis and observational datasets.

Overall this manuscript will be a useful contribution to the literature. I have some detailed comments below. However, I am not sure that this is an ideal candidate for Nature Communications for two reasons. First, I am not convinced that the results "... represent important advances of significance to specialists within each field." The results are interesting ... but I am not sure I would call them "important advances". The idea that cold conditions in the eastern tropical Pacific drive aridity over the western US has been thoroughly explored and documented in the literature, so that aspect may not be an important advance. Secondly, when a manuscript includes 29 figures in the Extended Data section, I question if it is really suitable for a short format journal. There are many times in the manuscript when the reader is referred to the Extended Data section to support important ideas and concepts. For example, the results of the prescribed SST experiments - an important component of the argument - are briefly discussed in the main body (lines 264-272) but the map of the results is relegated to Extended Data Figure 14. Even a short paper should be able to largely stand on what is in the main paper, and I am not sure that is the case here. I feel this work would be much better suited for a longer format journal.

The science in the manuscript is sound but arguably more incremental than a major advance. Others may feel differently, and I would certainly respect those assessments, but it is my assessment that this manuscript would be an excellent contribution to a longer format journal, where material in the Extended Data section could be folded more naturally into the main body of the manuscript.

On another general note, I find the figure quality to be below average. One specific approach that could help (for example in Fig. 1) would be to only stipple or obscure areas that are not statistically significant, thus allowing a clearer visual inspection of areas that are significant and thus clear of any stippling. I also found the color of the coastlines to not stand out. Also, the text in the figure labels appears blurry. Overall, the figures are not as clear and crisp as I would hope for.

Specific questions/comments

1. For the water-tagging ... is it the case that the water is tagged from the location it first evaporates into the atmosphere until the point where it precipitates to the surface? Is the "memory" of the tagging lost after that point? For example, if there was evaporation over the Pacific which fell out as precipitation over California, but was then evaporated again and fell out as precipitation over Colorado ... would the "source" for that precipitation over Colorado be California (continental) rather than the Pacific (oceanic)? With multiple re-evaporation cycles possible for water parcels how useful is that for tracking longer range sources of moisture? Also, what assumptions are made about moisture parcel transport during any nonlinear atmospheric processes, such as convection?

2. Line 111 The units for the trend of specific humidity are listed here as "%/decade" whereas the figure caption (1b) lists "g/kg/decade". There should be consistency between the text and figure.

3. Line 126 Should "anomalies" be "positive linear trends:?

4. Line 133 The "wave train emanating from the tropical Eastern Pacific" is not obvious to me from Extended Data Fig. 3. Perhaps clarify that with some additional text.

5. Line 137 Do you mean no change in the trend, or no trend? Also, line 135 refers to "minimal SST warming trends" whereas line 139 refers to "SST cooling". Line 223 refers to "muted warming". Why the different terminology?

6. Line 178 Isn't it usually the case that enhanced downward motion is associated with reduced precipitation?

7. Line 234 Is it accurate to say "the same climate forcing" here since different models have different radiative responses to identically imposed changes in atmospheric composition?

8. Line 256 Since this discussion refers, I believe, to comparisons within members of the CESM2 ensemble, I don't think it is accurate to say at this point in the paper "driven by tropical Pacific SST cooling in the model" as that causality has not yet been shown ... that statement needs the fixed SST experiments discussed later.

9. Line 306 ... typo with extraneous left bracket.

10. Line 342 I was a little confused by the "two-way capacitor" analogy.

(Remarks on code availability)

Version 2:

Reviewer comments:

Reviewer #3

(Remarks to the Author)
I thank the authors for the revision. While the concerns I have previously expressed remain in place, I respect the judgement of the other reviewers and Editors and therefore am supportive of publication.

A few specific minor comments ...

1. Line 167 The text discusses the soil moisture trend in Fig. 3d, but the text along the top of the plot says evaporation. I suspect that should be "soil moisture".

2.Line 288 The text implies that we should be able to see from Fig 8a that zone 41 contributes 20% of the total atmospheric moisture over the Western US, but the figure shows the contribution to the trend. I believe this is only seen from Extended Data Fig. 13a.

3. I still o not find the water tagging experiments to be especially illuminating for the reasons I previously mentioned (the potential for a water parcel that is transported from a different region can be precipitated out and re-evaporated one or more times, thereby complicating the interpretation of the results).

(Remarks on code availability)

Open Access This Peer Review File is licensed under a Creative Commons Attribution 4.0 International License, which permits use, sharing, adaptation, distribution and reproduction in any medium or format, as long as you give appropriate credit to the original author(s) and the source, provide a link to the Creative Commons license, and indicate if changes were made.
In cases where reviewers are anonymous, credit should be given to 'Anonymous Referee' and the source.
The images or other third party material in this Peer Review File are included in the article's Creative Commons license, unless indicated otherwise in a credit line to the material. If material is not included in the article's Creative Commons license and your intended use is not permitted by statutory regulation or exceeds the permitted use, you will need to obtain permission directly from the copyright holder.
To view a copy of this license, visit https://creativecommons.org/licenses/by/4.0/

We greatly appreciate the continued review and the insightful comments provided by the two original reviewers. To address the remaining issues, we have made the following revisions to the manuscript:

1. In the main text, we replace the PAC minus CESM2-LE analysis with new AMIP simulations conducted using two AGCMs (CAM5 and ECHAM5). In these experiments, we examine the atmospheric response to a prescribed SST cooling pattern represented by an idealized oval-shaped tropical SST anomaly that resembles the observed eastern-tropical Pacific SST cooling over the past 41 years. The consistent responses across both models highlight the important role of tropical eastern Pacific SST cooling in shaping the Moistening Hole (MH) pattern. We believe these new experiments provide a more direct and cleaner assessment of tropical influences on the MH compared with our original PAC minus CESM2-LE comparison.

2. In the response below, we also clarify why nudging near-surface specific humidity (the model's lowest layer) strengthens our argument, while emphasizing that our main conclusions do not rely heavily on this nudging step. Our goal in the nudging and water-tagging experiments is to reproduce a hydrologic cycle that is more consistent with ERA5 meteorological fields over the past 41 years. Because the tagging scheme primarily reflects evaporation signals from source regions, improving the simulation of evaporation ensures that the water-tagging results are closer to reality. For this reason, we nudge model fields toward ERA5 as much as possible without disrupting the model's internal physical consistency. Since evaporation, based on the bulk formula, is sensitive to surface and near-surface specific humidity, nudging Q in the model's lowest layer provides a more realistic constraint on evaporation. Importantly, Q nudged from ERA5 is not treated as a moisture source in the tagging scheme. We also show in the following response that this Q nudging does not alter the simulated long-term MH trend. In both Settings 1 (with Q nudging) and 2 (without Q nudging), the MH trends exhibit very similar spatial patterns, although day-to-day (not shown) and year-to-year variability of specific humidity within the MH region differ somewhat.

3. We also include additional results in the response below showing that increasing CO_2 forcing in our nudged experiments (Setting 2 with increased CO_2 and no Q nudging compared with Setting 3 with constant CO_2 and no Q nudging) does exert an influence, but not over the western U.S. This weak impact of physiological plant processes on the MH in this region is primarily due to the limited leaf cover in the western U.S., which reduces CO_2-driven physiological effects. This clarification directly addresses the reviewer's concern regarding the potential role of CO_2 forcing in shaping the MH pattern through vegetation mechanisms.

4. We added a brief discussion in the main text, before the results section, regarding uncertainties in the datasets and model configurations, to let readers be aware of the caveats in our methodological framework before engaging with the detailed analyses. Finally, all figures have been updated to better illustrate the spatial patterns of the variables of interest, and all key codes used in this study have been made publicly available through our GitHub repository.

Below, we provide detailed point-by-point responses (highlighted in blue) to the reviewers' comments. We hope these revisions and additional analyses have fully addressed all concerns raised.

Reviewer #1 (Remarks to the Author):

This study investigates the cause of the moistening hole–like change over the Western U.S. in the context of global warming. By analyzing a wide array of observational data, CMIP6 runs, CESM2-LE experiment, sea surface temperature pacemaker runs, and water-tagging enabled simulations, this article reveals that sea surface temperature cooling in the tropical eastern Pacific can induce a barotropic high-pressure circulation trend over the North Pacific, which in turn enhances atmospheric subsidence and reduces atmospheric moisture over the region. The conclusions drawn from the analysis are generally sound and suggest important implications. The story in this paper isn't particularly groundbreaking but is interesting.

The revision is clearer and better organized, and the authors have largely provided clear answers to the reviewers' questions. It is potentially publishable after the authors address several additional questions and comments listed below.

We thank the reviewer for the positive comments and hope that our revisions will meet your standards.

Major comments:

Although the difference between the PAC results and CESM2-LE ensemble mean indicates a drying trend over the western U.S., the tropical Pacific SST anomaly in this case represents the difference between the observed SST trend and an El Niño-like SST anomaly. Therefore, I am uncertain about the magnitude and significance of the observed SST changes' specific impact and contribution, as well as the degree of SST nonlinearity. Since the authors have already performed numerous experiments, there is no need for additional simulations. However, it would be helpful if the authors could clarify how they understand and explain this result. For example, could the authors further show the differences in composite linear annual-mean trends between the 15 coldest and the 15 weakest SST-trend members of CESM2-LE, as well as between the 15 warmest and the 15 weakest SST-trend members, in addition to Extended Data Fig. 13？

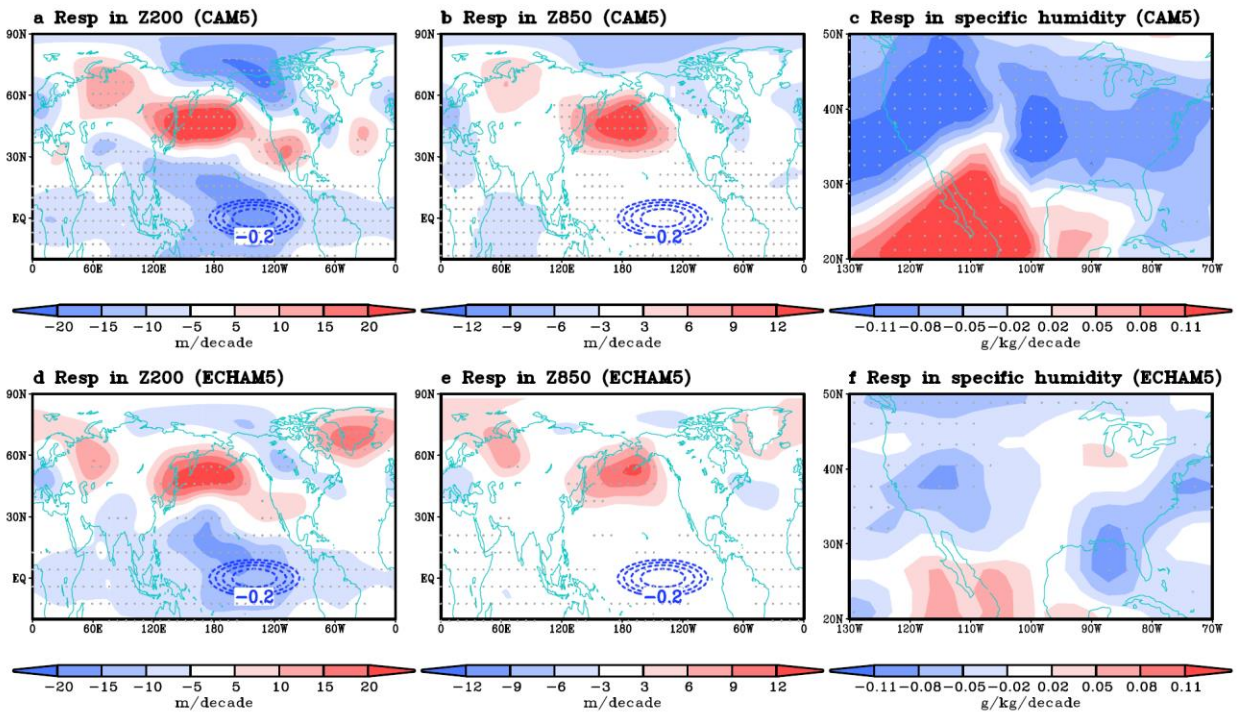

Resp. Fig 1 (also Extended Data Fig. 14). Differences between the 30-year averages of annual-mean (a, d) Z200, (b, e) Z850, and (c, f) lower-tropospheric specific humidity averaged between 650 and 1000 hPa for the sensitivity run relative to the control run, shown for CAM5 (upper panels) and ECHAM5 (lower panels). The prescribed SST cooling anomaly in the sensitivity run relative to the control run (contour interval: -0.2 °C) is shown by blue dashed contours. Regions where the differences between the two runs are significant at the 95% confidence level are indicated by dots, based on a two-sample t test.

We appreciate Reviewer 1's comment and fully agree that comparing PAC and CESM-LE is not an effective way to assess the role of tropical forcing in driving the MH feature. Therefore, in the revision, we remove that analysis and replace it with two new AMIP-type simulations using CAM5 and ECHAM5. For each model, we conduct two sets of experiments: (1) a control run with climatological SST and sea ice everywhere and fixed anthropogenic forcing, and (2) a sensitivity run with the same configuration (SST/sea ice, initial conditions, and anthropogenic forcing) but with an imposed tropical SST-cooling pattern added in the eastern tropical Pacific. We integrate both models for 30 years to evaluate whether the atmospheric responses are robust across two distinct model physics. The imposed cooling is an idealized pattern with a maximum amplitude of −1 °C at the center and zero cooling along the edges (blue contours in Resp. Fig.1 a&b&d&e), mimicking the observed SST cooling trends.

The differences between the sensitivity and control runs over the 30-year integrations (with significance assessed using a two-sample t test, Resp. Fig. 1) highlight the key role of tropical Pacific cooling in driving large-scale circulation changes in and around the western U.S., These simulations clearly show that the sensitivity-minus-control response produces an anomalous barotropic high pressure over the North Pacific, which favors an MH-like pattern over the western

U.S. Both models exhibit very similar wave-train patterns across the NH, although the MH feature is stronger in CAM5 than in ECHAM5. These experiments also support our physical interpretation summarized in the schematic diagram (Extended Data Fig. 19).

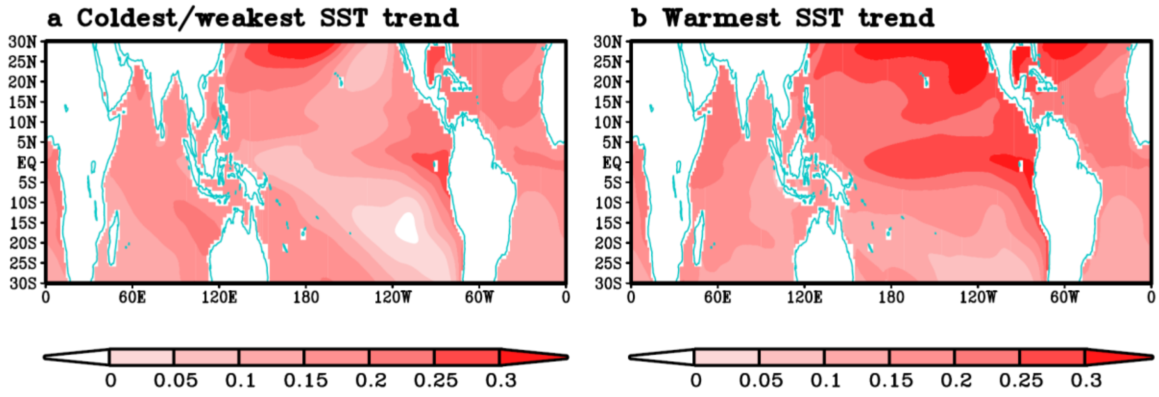

Resp. Fig. 2 Linear annual-mean SST trends (1980 to 2020) derived from the 15 coldest and 15 warmest SST-trend members of CESM2-LE.

Regarding the second concern, we find that almost all ensemble members in CESM2-LE show some degree of warming from 1980 to 2020, ranging from slight to strong warming, in the eastern tropical Pacific (Resp. Fig. 2). Thus, the "coolest" group corresponds simply to the members with the weakest warming trend. This indicates that, under CO2 forcing, the CESM2-LE configuration does not produce an SST change comparable to the observed cooling, even when considering internal variability across 100 members. Therefore, constructing a composite of 'weakest minus warmest' or 'weakest minus coldest" is not pertinent in this context. In lines 258, We emphasized that the 15 Pacific-cooling members are those defined relative to the ensemble-mean trend.

Minor comments:

1. Line 49, please change "represent" in "represents".

This typo has been corrected.

2. Lines 50-51, The word "droughts" in the original sentence is somewhat repetitive; consider revising the sentence "contributing to prolonged meteorological droughts and related droughts in the agricultural, hydrological, and socioeconomic sectors of the region" to "contributing to prolonged droughts across the meteorological, agricultural, hydrological, and socioeconomic sectors of the region".

Thanks for the wording suggestion. We have rewritten the sentence as suggested.

3. Line 72-73: You could mention this paper about the impact of the West Pacific (WP) teleconnection. https://agupubs.onlinelibrary.wiley.com/doi/full/10.1029/2024EF004922

Many thanks! This paper has been cited in the revision as reference 39.

4. Line 86, please change "forcing" in "forcings".

Done.

5. Line 110. It seems that there is also a significant decrease in specific humidity over central-eastern Africa, north of the equator.

We also rewritten this in the main text to make our discussion more accurate: ‘This drying trend in the lower troposphere is the most pronounced across the NH extratropics, both in magnitude and spatial extent.’ (at lines 114-116).

6. Lines 165-169. I agree that reduced evaporation may play a more direct role in atmospheric drying, but why does the decreasing trend in precipitation appear slightly stronger than that of evaporation over the western U.S.?

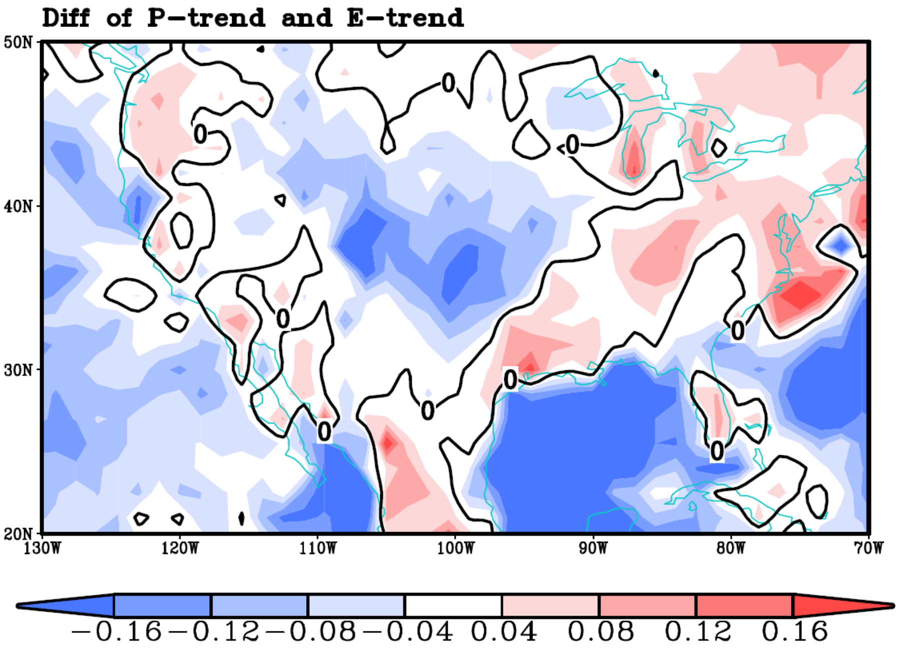

Resp. Fig.3 Linear trend differences in annual-mean precipitation and annual-mean evaporation.

If we understand this question correctly, the reviewer is asking how to reconcile the difference between the precipitation trend and the evaporation trend with the MH feature. The reviewer is correct that, over parts of the western US, the decreasing trend of precipitation is slightly stronger than that of evaporation (Resp. Fig. 3). In this case, the negative trend of P−E would imply a moistening effect for the in situ atmospheric specific humidity. Combined with the fact that the moisture-flux divergence shows no trend in our analysis, one would expect a moistening trend in local specific humidity, which contrasts with the observed MH pattern. This is a reasonable concern.

As we emphasize in the paper (lines171-175), the moisture budget involving long-term trends of these fields is difficult to close because the local specific humidity tendency is a very small residual compared with the magnitudes of precipitation and evaporation. As a result, its long-term trend is highly sensitive to even small changes or uncertainties in either precipitation or evaporation.

Specific humidity in reanalyses is strongly constrained by data assimilation (for example, radiosonde and satellite observations), whereas precipitation and evaporation are largely determined by the model physics rather than the assimilated data. Moisture fluxes are diagnosed from model winds and humidity fields, each carrying their own uncertainties. This mismatch is a limitation of reanalyses. This is also why we need to use an alternative approach (water tagging) to understand the role of evaporation in contributing to the MH feature.

7. Lines 238-240. Circulation and SST trend patterns between the 15 coldest and 15 warmest SST-trend members of CESM2-LE are shown in Extended Data Fig. 13, rather than Extended Data Fig. 12. Please revise it.

In that part, we still discuss the 15 driest members versus the 15 wettest members. Accordingly, Extended Data Figure 12 (correctly referenced) presents the composite of local hydroclimatic changes in the western U.S.

8. Line 253, please change “dying” in “drying”.

Sorry for the typo, which has been corrected.

9. Lines 266-269. The PAC run fails to reproduce the observed vertical motion trend over the central and eastern U.S. The authors attribute this mainly to the regions being relatively distant from the SST forcing. I think it might be helpful to briefly mention that the ERA5 trend in this region could also be influenced by other factors, such as Arctic sea-ice loss or other remote forcings.

Thanks for this suggestion. We have removed this part using the PAC runs.

10. Lines 320-321. Does the annual mean IVT also show weak trends over these two regions?

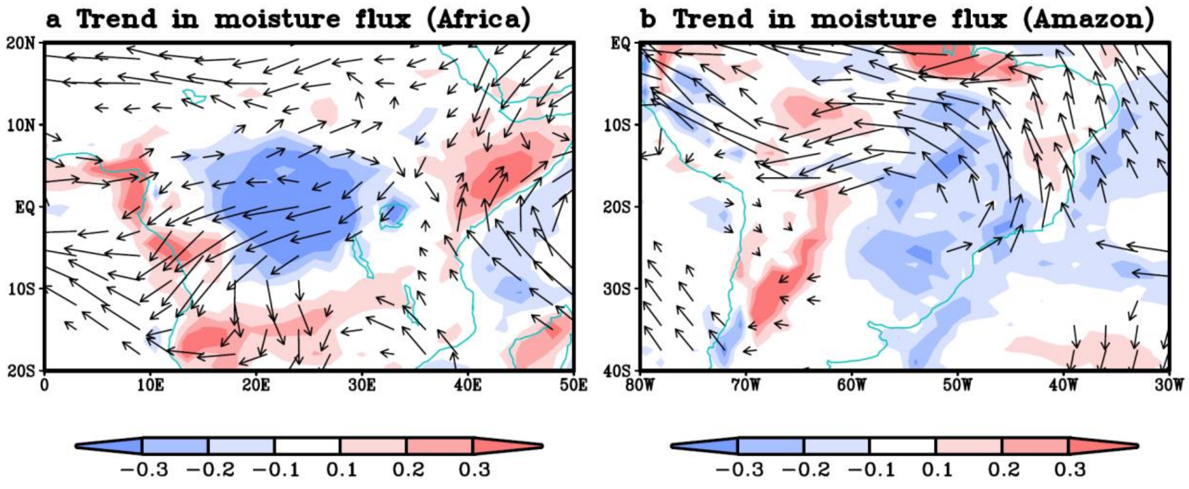

Resp. Fig. 4. Linear trends of annual-mean IVT and IVT divergence (IVT derived from hourly data). Positive shading indicates moisture convergence. Results are shown for ERA5 during 1980–2020 over a) Central Africa and b) the Amazon.

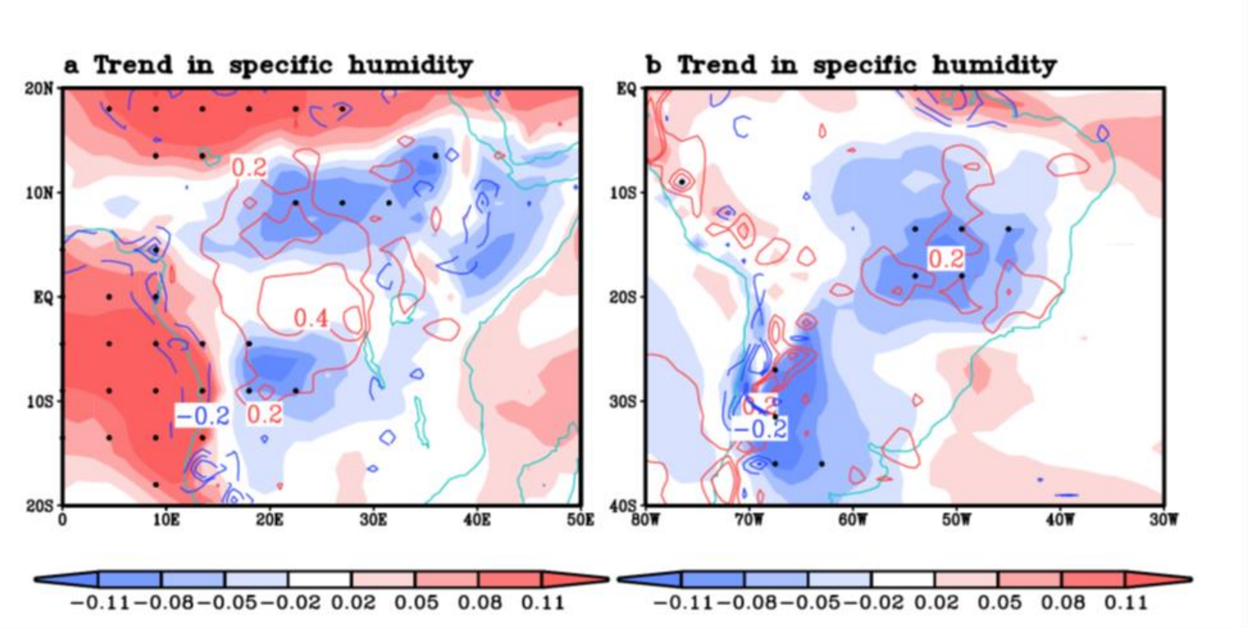

Resp. Fig. 5 (also Extended Data Figure20): Linear trends of annual-mean specific humidity (averaged between 650–1000 hPa, g/kg/decade, shading) and tropospheric vertical motion (averaged between 300–800 hPa, contours) over (a) Central Africa and (b) the Amazon from ERA5 during 1980–2020. Trends in specific humidity significant at the 95% confidence level are marked with dots.

We plotted these two fields (Resp. Fig. 4) along with the original figure showing the trend of specific humidity (Resp. Fig. 5) for your information. Over Central Africa, the trends in moisture flux and its divergence are significant but do not align with the spatial pattern of specific-humidity trends. In contrast, over the Amazon, a clear divergence trend pattern is co-located with the drying in specific humidity, indicating that the influence of moisture transport differs by region and is strongly region dependent.

11. Line 581, please change “horizonal” in “horizontal”.

Corrected.

12. Line 737, please change “precious” in ”previous”.

Corrected.

13. References 13 and 77 refer to the same article. Please verify this and make the appropriate correction.

Corrected. We have removed reference 77.

Reviewer #1 (Remarks on code availability):

I briefly reviewed the provided code and did not find any issues. However, I did not attempt to install or run the code.

We have added all key codes used in this study in our GitHub repository

Reviewer #2 (Remarks to the Author):

Review of "Regional drying over the Western U.S. driven by enhanced atmospheric subsidence amid global moistening from 1980 to 2020"

I thank the authors for their responses to my comments as Reviewer 1 from the [*REDACTED*] review process. I believe the revised work is interesting and important, but that it would be better placed in a long-form journal like Journal of Climate, within which the methodological choices could be discussed alongside with the results. In my mind, the primary contributions of the work are illuminating the dynamical links from tropical Pacific SSTs to the midlatitudes, as well as (potentially, see comments below) the results from the water tagging experiments. These will be of great interest to a climate audience if the paper is written such that the methods and results are more transparent and clear.

Thank you very much for your suggestion. We add a short summary after the introduction to raise readers' awareness of the limitations of our approach (lines 98-103). We also note that the MH feature may be overestimated in ERA5, and this biased signal could carry over to our nudging run. With this addition, readers can gain a clear big-picture understanding of both the strengths and limitations of our approach and methodology, before going through the full methodological details in the Methods section.

Major comments

- There are two large comments from my prior review that I do not believe were addressed. The first is about the combination of water tagging and nudging. Based on the prior response, my best guess is that, when humidity is added at a given point through the increments, that the humidity is "sourced" from that location because the authors seem to be interpreting the nudging as reflecting a local source. If this is correct (and it may not be, but my question was not fully answered before), then I am skeptical of the water tagging results. We know that CESM "wants" to get more humid. The increments will presumably reduce humidity in the US Southwest. If these increments are viewed as local sources, then it will appear that the local sources are reducing over time. This needs to be clarified in the paper. The authors also did not answer my question about the similarities between Settings 2 and 3, which I believed to be surprising given the likely strong physiological plant response to CO_2 in the model.

Thanks for pointing out these two issues that we did not fully address in the last round. Regarding the first point, we agree that some of the confusion arose from our insufficient explanation of the goals of the nudging and water-tagging experiments. Here, we clarify this more explicitly. The objectives of this research consist of two components: (1) to understand the dynamical mechanisms driving the MH, and (2) to understand the observed hydrological processes associated with the

MH. With the new AMIP experiments (see below), we are confident that the causal mechanisms behind the MH are now more clearly demonstrated in the revision.

The second objective is to use iCESM to reproduce a hydrologic cycle that is more consistent with ERA5 meteorological fields over the past 41 years. We do not use this iCESM approach as a sensitivity experiment to examine the large-scale forcing of the MH. That goal is primarily achieved through our AMIP experiments (originally using PAC minus CESM2-LE).

Because the tagging scheme primarily reflects evaporation signals from all source regions, improving the simulation of evaporation ensures that the water-tagging results are closer to reality. For this reason, we nudge the model fields toward ERA5 as much as possible without interfering with the model's physical parameterizations that govern precipitation processes. Following the CESM development team's recommendation

(https://ncar.github.io/CAM/doc/build/html/CAM6.0_users_guide/physics-modifications-via-the-namelist.html?utm_source=chatgpt.com), we nudge only the model's specific-humidity field in the bottom layer and not at higher levels. Since evaporation, according to the bulk formula, is highly sensitive to surface and near-surface specific humidity, nudging Q in the model's lowest layer provides an optimal constraint on evaporation.

We also emphasize that the specified Q derived from ERA5 is not treated as a moisture source in the tagging scheme. Its role is to help determine surface evaporation so that the evaporation process is more strongly constrained by ERA5. We also performed two additional tests (Settings 2 and 3) without Q nudging, and the simulated MH pattern remains very similar (Resp. Figs. 6 and 7). For example, the MH drying signals produced by the two settings are quite similar, with Setting 2 showing slightly stronger drying than Setting 1.

Furthermore, the tagged source region over the western U.S. (New Mexico, 30–40°N, 250–260°E), which is the most important source region in our finer tagging experiment, exhibits nearly identical contributions to MH drying regardless of whether Q is nudged. In Setting 1 (the configuration used in our paper), this region accounts for 45% of the MH drying, whereas in Setting 2 (without Q nudging), it increases to 47%. This indicates that the long-term drying trend is robust across the two setups and that the leading role of local evaporation is not sensitive to whether Q is nudged. Although the year-to-year variability differs somewhat between the Q-nudged run (Setting 1) and the non-Q nudged run (Setting 2), the long-term trend remains nearly identical (Resp. Figs. 6 and 7).

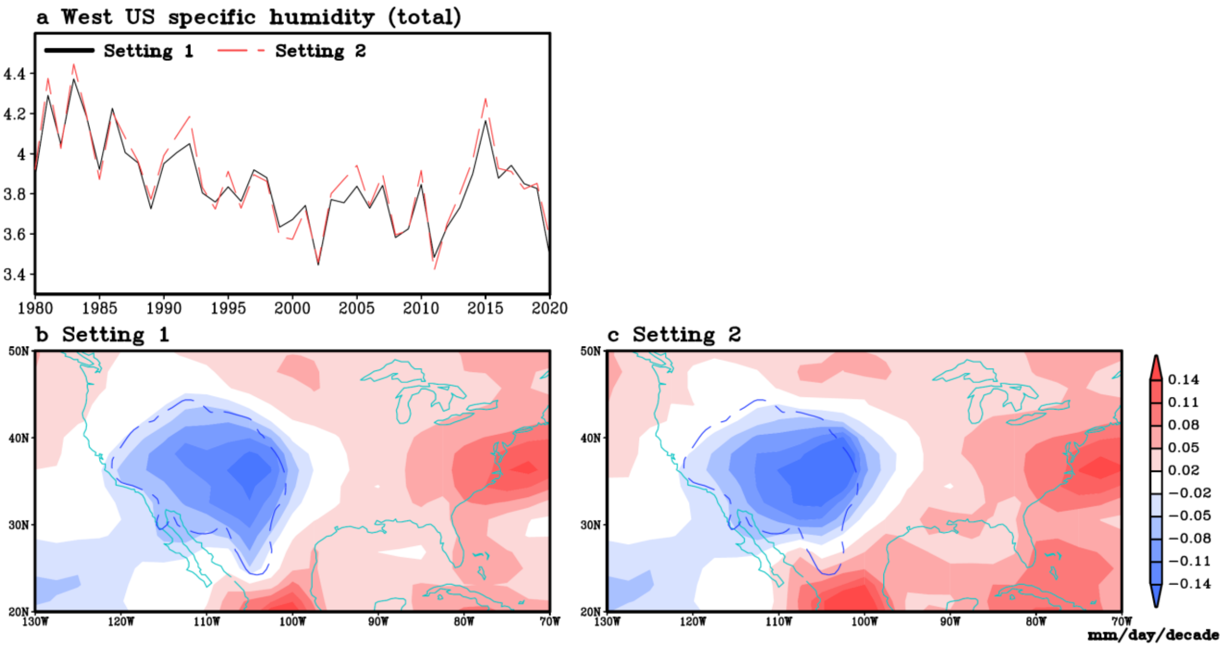

Resp. Fig. 6: (a) Time series of annual-mean lower-tropospheric specific humidity (averaged between 650 and 1000 hPa) over the western U.S. (within the MH contour in panel b) from 1980 to 2020 in our nudging experiments under Setting 1 (with Q nudging) and Setting 2 (no Q nudging), along with (b) the corresponding spatial patterns of linear trends in annual-mean lower-tropospheric specific humidity over 1980–2020.

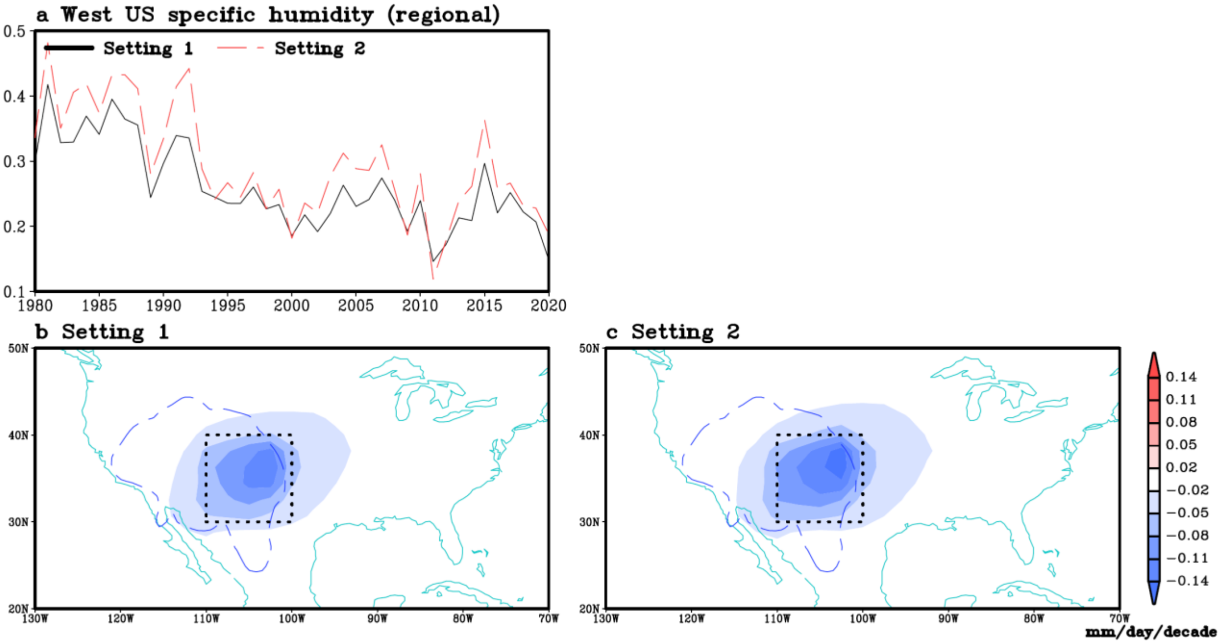

Resp. Fig. 7. (a) Time series of annual-mean lower-tropospheric specific humidity (averaged between 650 and 1000 hPa) over the western U.S. (within the dashed blue contour in panel b)

from 1980 to 2020, evaporated from the dashed box (New Mexico, 30-40N, 250E-260E) in Setting 1 and Setting 2, and (b) the corresponding spatial patterns of linear trends in annual-mean lower-tropospheric specific humidity over 1980–2020 evaporated from the source region (30-40N, 250E-260E, dashed box).

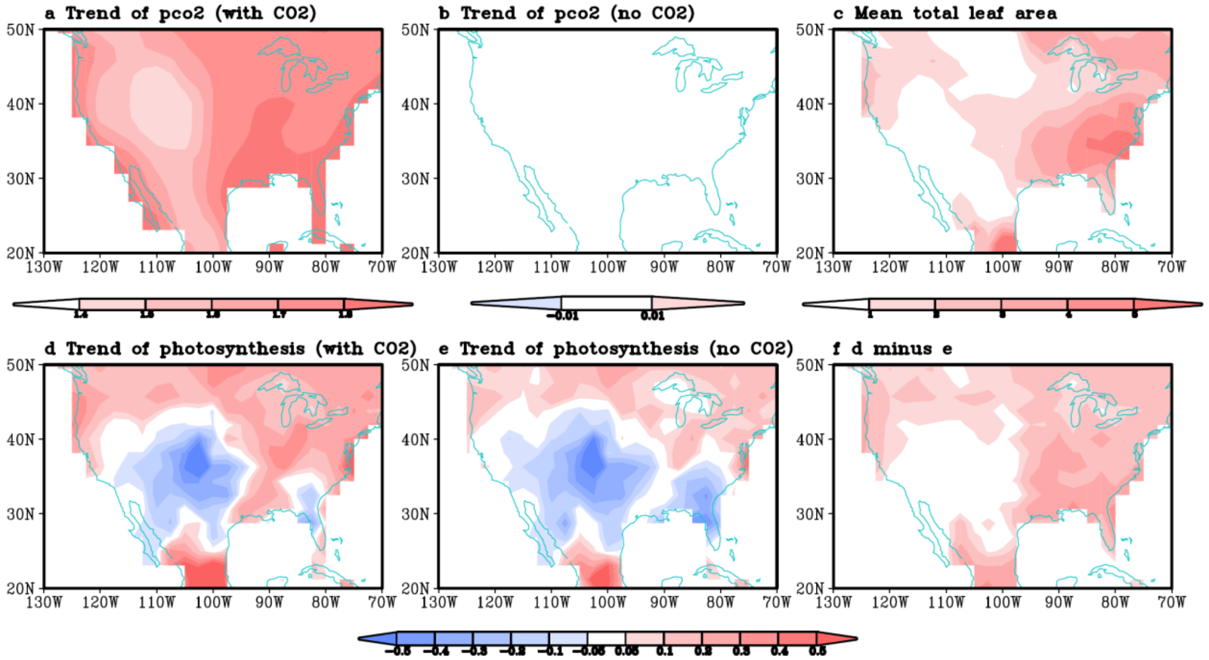

Resp. Fig. 8 a) Linear annual-mean trends of partial CO2 pressure (unit:Pa/decade) in Settings 2 (increasing CO_2) and 3 (constant CO2). (b) Long-term mean total leaf area index (TLAI) in Setting 2, indicating the vegetation cover used in the model. (c–e) Linear annual-mean trends of photosynthesis (μmol m^{-2} s^{-1}/decade) in Settings 2 and 3, and (f) the difference between the two trends from d and e. Positive values indicate enhanced photosynthesis driven by increasing CO2.

Regarding the second concern, we include Resp. Fig. 8 to illustrate this point more clearly. In Setting 2, CO2 increases at approximately 17 ppm per decade (from about ~336 ppm to ~411 ppm over the 41-year period), whereas CO2 is held constant in Setting 3. This forcing difference is reflected in the trend of partial CO2 pressure for leaf (an output of the land model in iCESM) in the two runs: about 1-2 Pa per decade in Setting 2 and zero in Setting 3.

The comparison between these two configurations (Setting 2: increasing CO2 without Q nudging; Setting 3: constant CO2 without Q nudging) further shows that the trends in photosynthesis rates over the western U.S. are very similar between the two settings. The main differences in photosynthesis trends occur over the eastern U.S., where the total leaf area index (TLAI) is much larger (Resp. Fig. 8c). In contrast, the physiological impact of CO2 forcing on the MH region in the western U.S. is very weak because vegetation cover there is extremely limited (Resp. Fig. 8c). The very small TLAI in the western U.S. implies that CO2-induced stomatal responses (not via temperature) contribute minimally in that region. We hope this clarification directly addresses the

reviewer's concern regarding the potential role of CO_2 forcing in shaping the MH pattern through physiological mechanisms.

In summary, our nudging experiments across all three settings are broadly consistent and all indicate a significant role of local evaporation in driving the MH. Nudging Q is not required, and omitting it actually makes the contribution of local processes even stronger. Because nudging Q in the boundary layer provides a better physical constraint on evaporation, we use Setting 1 in the main text (this point is emphasized in lines 687-689). Under this configuration, the 45% contribution from the small box (30–40°N, 250–260°E) and the 88% contribution from Region 41 to the full MH trend can be interpreted as lower-bound estimates of the local contribution. If we use Settings 2 or 3, these values increase.

- I appreciate the addition of the experiment where the nudging is only outside the western US. However, the authors large brush over the fact that the humidity response is much smaller (i.e. the trend less negative) in that simulation. This suggests that the large-scale forcing may not be playing as much of a role as has been argued elsewhere. It would be great to see further discussion of this in a longer-form paper.

Since our goal in using the nudging runs is to reproduce the hydrologic cycle driven by ERA5 meteorological fields, rather than to conduct a sensitivity experiment to test the large-scale forcing on the MH, it is natural to see a weaker MH feature when the nudging strength is relaxed. This weakening does not conflict with our conclusions, because the large-scale forcing in this run (without nudging in the box) does not fully reflect the realistic changes. To examine this point from a true sensitivity perspective, we use the AMIP run shown below to confirm that the tropical SST forcing helps maintain a more robust and spatially coherent MH pattern. In the revision, we have emphasized that the nudging result is sensitive to ERA5 data, which may overestimate the intensity of the MH trend compared with other reanalyses (lines 98-103, 701-702).

- In their focus on the role of the tropical Pacific, the authors compare PAC minus CESM2 to the observations, which is confusing. As they note, PAC minus CESM2 (ideally) isolates the role of tropical Pacific SST forcing. But the observations result from both tropical Pacific SST forcing and the ongoing anthropogenic forcing, and the latter is removed when subtracted CESM2 from PAC. I think the text needs to be reworded to note that the tropical Pacific SST forcing induces a drying over the western US, but that the PAC ensemble as a whole still has positive trends in specific humidity everywhere. This then suggests that the damped / lack of warming in the eastern Pacific is playing a role in the lack of humidity increase, but it is not the whole story, because when anthropogenic forcing is included (in PAC), the region still moistens.

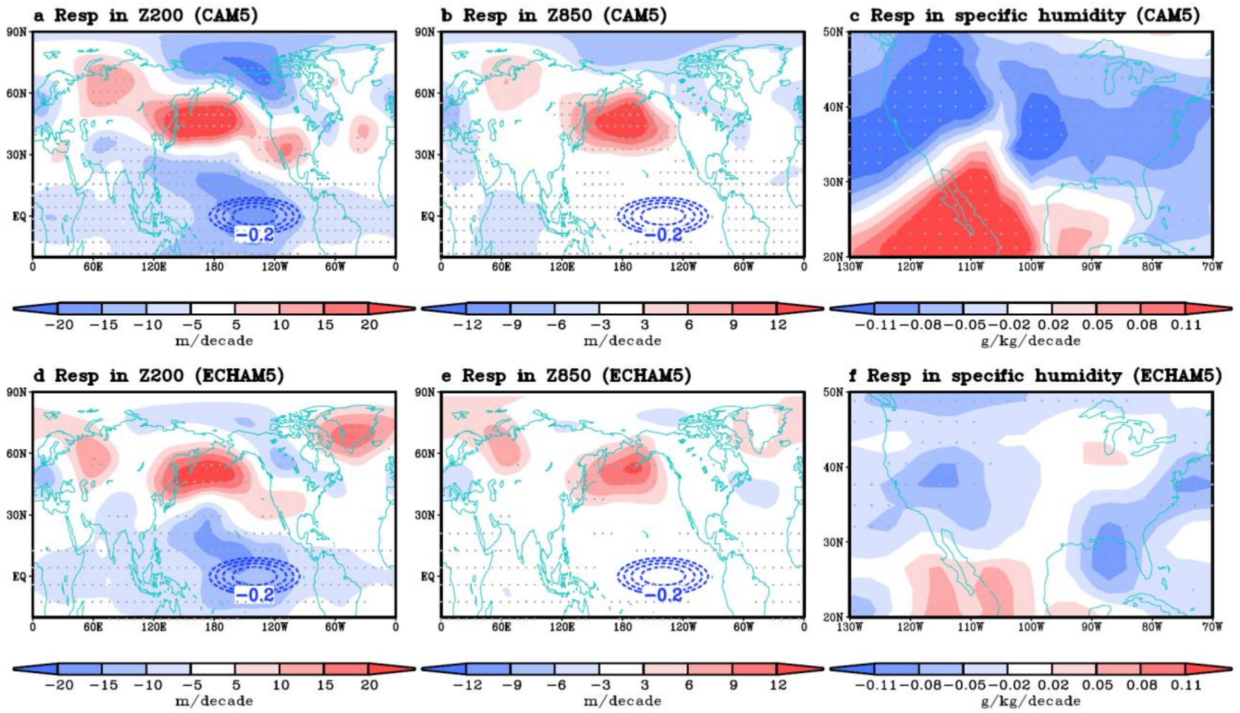

Resp. Fig 9 (also Extended Data Fig. 14). Differences between the 30-year averages of annual-mean (a, d) Z200, (b, e) Z850, and (c, f) lower-tropospheric specific humidity averaged between 650 and 1000 hPa for the sensitivity run relative to the control run, shown for CAM5 (upper panels) and ECHAM5 (lower panels). The prescribed SST cooling anomaly in the sensitivity run relative to the control run (contour interval: -0.2 °C) is shown by blue dashed contours. Regions where the differences between the two runs are significant at the 95% confidence level are indicated by dots, based on a two-sample t test.

This is the same concern raised by Reviewer 1. We greatly appreciate this point and fully agree that comparing PAC and CESM2-LE may not be an effective way to test the role of tropical forcing in driving the MH feature. Therefore, in the revision, we remove that analysis and replace it with two new AMIP-type simulations using CAM5 and ECHAM5 (Resp. Fig. 9). For each model, we performed two sets of runs: (1) a control run with climatological SST/sea ice and fixed anthropogenic forcing, and (2) a sensitivity run with the same configuration (SST/sea ice, initial conditions, and anthropogenic forcing) but with an imposed tropical SST-cooling pattern added in the tropical Pacific. We integrate these experiments for 30 years in both models to examine whether the responses are robust across two distinct model physics. The imposed cooling is an idealized pattern, with a maximum amplitude of −1 °C at the center and zero cooling along the edges (blue contours in Resp. Fig 9), resembling the recent observed SST cooling in the tropical Pacific.

The differences between the sensitivity and control runs over the 30-year integrations (with significance assessed using a two-sample t test) highlight the key role of tropical Pacific SST cooling in driving circulation changes in and around the western U.S. These simulations clearly show that the sensitivity-minus-control response produces anomalous barotropic high pressure

over the North Pacific, which favors an MH-like pattern over the western U.S. Both models exhibit similar large-scale circulation responses in the NH, although the MH feature is stronger in CAM5 than in ECHAM5.

- Regarding code availability, the authors wrote: "Other analyses (e.g., composites, trend analysis, correlation calculations, and two-sample tests) are standard and therefore not included in the repository." I strongly suggest (and Nature Comms may require?) that all code is made available. I have experienced many times confusing wording in a manuscript in terms of methods that can be immediately clarified in code. Further, other researchers cannot easily build off your code and results if many of the relevant pieces are missing.

As suggested by the reviewer, we have added all key codes used in this study to our GitHub repository.

- Please improve the quality of the figures for clarity. The colorbars remain very coarse, and I don't see a reason to use a different number of color blocks per figure. Using 8-10 is a reasonable balance between providing interpretable figures and not getting too close to a continuous colorbar. In the line plots, the lines are very thin, and it is often difficult to even see the dashed or dotted lines. The figures all seem to be have been saved at low resolution.

We also revised all figures to use an eight-color scale with clear lines and contours. We have tried our best to improve their overall quality.

Line-specific comments

- L64: "Alternatively... limited attention in previous research focused on this region." There is an enormous amount of research linking Pacific variability to hydroclimate in the US SW.

We agree. This statement has been rewritten as “a potential driver that has received sustained attention in recent research focused on this region” at line 66.

- L104: LOWER troposphere

Added. Thanks!

- L118: Incorrect figure reference

Corrected.

- L193: Many comparisons of the observations to the ensemble mean have been modified, but some remain, including in this paragraph.

We have rephrased some of our discussion in this paragraph as suggested "These ensemble-mean responses suggest that the observed sinking motion trend over the Western U.S. does not line up well with the model's mean response to global warming." at lines 208-210.

- L229: Suggest different word than "fingerprint" which is typically associated with D&A/optimal fingerprinting. This is a composite analysis.

We have replaced 'fingerprint' with 'composite

- L244: The composite analysis does not provide evidence that tropical Pacific SSTs "drive" the MH, but rather that they are related. The compositing on Pacific SST is more convincing, but still cannot be used to suggest causality.

We agree. Partly motivated by this comment, we have added the AMIP runs in the revision to further support this argument. We believe this addition clearly demonstrates the causality of the MH and attributes it to Pacific SST forcing.

- L259: As mentioned in my prior review, the CESM2-LE has different forcings for the first and second 50 members in terms of the biomass burning.

We have emphasized this difference in the revision at lines 490-494.

- L516: Based on the prior review discussion, I do not believe that the IGRA data being used are homogenized. Given the known, large inhomogeneties in radiosonde humidity data, this should be discussed explicitly. Also, the difficulty of homogenizing radiosonde data should be noted, so ERA5 may also be affected by inhomogeneities.

Thanks. We emphasized in the main text that ERA5 specific humidity is subject to substantial uncertainty in the original observational sources, which may affect our conclusions quantitatively but not qualitatively. (lines 98-103, 569-570,701-702)

Reviewer #2 (Remarks on code availability):

The code does not allow for full reproduction of the results (also see my comment in the main review), and is in Fortran, which few scientists use now.

As a long-time user of Fortran, all of my calculations were originally implemented in Fortran. I was able to convert the codes to Python using an online tool. However, as a beginner in Python, I am not confident that the converted version is completely bug-free. Therefore, I will upload only my original Fortran code and leave the full conversion to future users, as there are many tools available to complete this task reliably. Even for Python users, although my code cannot be used directly, the clearly added comments can still help them understand the calculation procedures.

Reviewer #1 (Remarks to the Author):

Review of "Regional drying over the Western U.S. driven by enhanced atmospheric subsidence amid global moistening from 1980 to 2020"

After two rounds of revision, I find that this study, based on analyses of reanalysis datasets and multiple numerical simulations, offers a convincing explanation for the emergence of the moistening hole–like signal over the western United States. In particular, the use of iCESM with water tagging provides a novel methodological approach for hydroclimate research over the western United States. Now, I have only three minor comments.

We greatly appreciate your assessment that our study is scientifically and technically valuable for improving understanding of long-term hydroclimate variability over the western US. Many of your comments have strengthened our presentation of the processes underlying the formation of the MH, including (i) the suggestion to use the simulated tropical SST trend as a selection criterion to identify a subset of CESM2 ensemble members and construct a composite, and (ii) the recommendation to add a schematic diagram. In the revised manuscript, we have moved the schematic diagram from the Supplementary Material to the main text, since Nature Communications allows more figures and Reviewer 2 also suggested improving the readability of the main text by reducing the number of supplementary figures.

1. In Resp. Fig. 1, could the authors clarify whether the units are correct? If the figure shows differences between 30-year mean states, these quantities should represent mean differences rather than time-dependent changes (e.g., per decade)

The reviewer is correct that the unit shown on the color bar should not be "per decade." We have removed "/decade" and corrected the color-bar label. The pattern shown is the raw difference between the 30-year mean of the sensitivity run and the 30-year mean of the control run. Therefore, the units are m for geopotential height and g kg^{-1} for specific humidity.

2. For the CAM5 and ECHAM5 experiments, how large is the magnitude of the North Pacific circulation response? In particular, how does it compare with the observed changes over the past 41 years? Quantifying how much of the observed strengthening of the North Pacific circulation can be attributed to the imposed tropical cooling would help clarify the contribution and impact of tropical SST forcing.

In our AMIP experiment, we prescribe an idealized, oval-shaped SST anomaly over the eastern Pacific, set to 0°C at the edges and −1°C at the center. The resulting area-mean anomaly over the forcing region is approximately −0.4°C, chosen to mimic the magnitude of eastern Pacific SST variability inferred from recent decades. In observations (Resp. Fig1) , the raw SST trend over the tropical eastern Pacific is small because it is embedded within a basin-wide tropical warming of about 0.5°C over the past 41 years (≈0.1°C per decade) across much of the tropical zone. After removing this tropical-wide warming at each grid point, the eastern Pacific exhibits an

apparent cooling of roughly −0.1 to −0.2°C per decade, equivalent to about −0.4 to −0.5°C over 41 years in an area-mean sense over the tropical eastern Pacific.

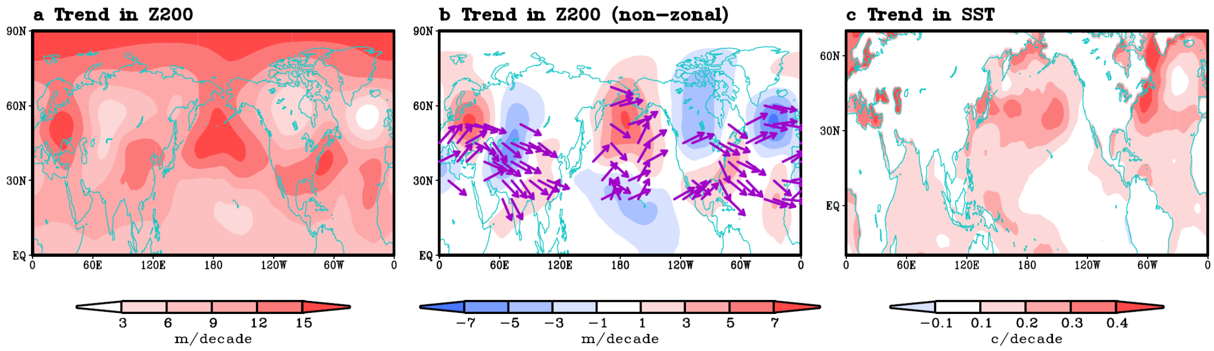

Resp Fig.1 (also Extended Data Figure3): Linear trends of (a) annual mean 200 hPa geopotential height (Z200, m/decade), (b) the non-zonal component of the annual mean Z200 trend, and (c) annual mean SST from ERA5 (ERSST5 for SST) during 1980–2020. In (b), vectors indicate the Plumb (1985) wave activity associated with the non-zonal wave pattern of the annual mean Z200 trend.

Although we could impose this observed SST pattern directly, we expect that eastern Pacific cooling, regardless of its exact spatial structure, could drive a similar large-scale teleconnection and thus contribute to the MH response. We use an idealized oval-shaped anomaly to test the circulation response in both models while ensuring a smooth transition from the boundary to the center. This setup yields an area-mean SST anomaly of about −0.4°C, comparable to the magnitude inferred from the 41-year SST record after removing tropical-wide warming. Thus, the difference between the 30-year sensitivity run and the 30-year control run can be interpreted as an estimate of how eastern Pacific cooling over the past 41 years (absent the influence of global warming) drives large-scale circulation changes.

In CAM5 (Resp. Fig.2 also Fig. 7 in the main text), the 30-year sensitivity-minus-control difference produces Z200 and Z850 anomalies of about 25 m and 12 m over the North Pacific (about 20 m and 10 m in ECHAM5), respectively. Direct comparison with observed trends should be made cautiously, because the observed changes (e.g., Z200 ≈ 15 m per decade, or ≈ 60 m over 41 years) reflect a combination of global warming, tropical SST cooling, and other forcings, whereas our experiment isolates the response to SST cooling alone. After removing the zonal-mean component of Z200, which we interpret as partially representing the influence of global warming, the observed Z200 trends are on the order of ~4–5 m per decade (Resp Fig.1, ≈ 20 m over 41 years), similar in magnitude to the simulated response in CAM5 and ECHAM5.

In contrast, it is difficult to separate other forcing signals from observed humidity trends, so only a qualitative comparison is possible. In ERA5, the MH drying is about −0.1 g kg^{-1} per decade (≈−0.4 g kg^{-1} over 41 years), whereas the simulated response is around −0.15 g kg^{-1} in CAM5 and weaker in ECHAM5 (≈−0.06 g kg^{-1}). Both models produce a weaker MH drying signal than ERA5 (Resp. Fig. 2).

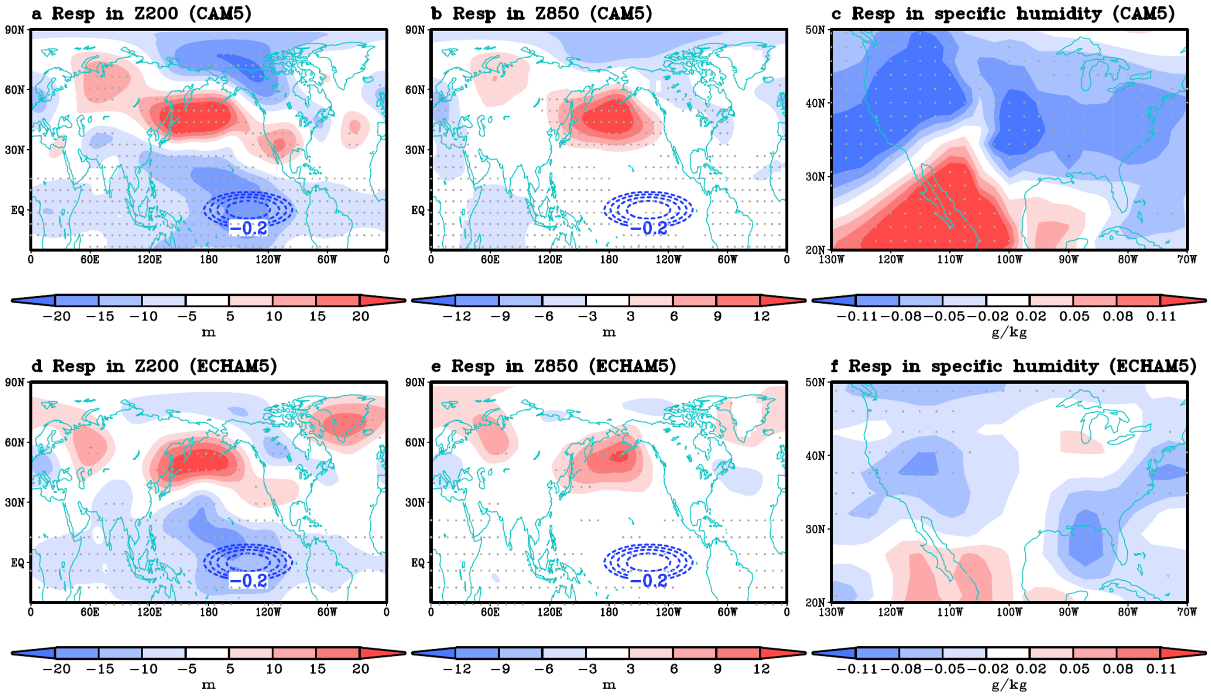

Resp. Fig 2 (also Figure 7): Differences between the 30-year averages of annual-mean (a, d) Z200, (b, e) Z850, and (c, f) lower-tropospheric specific humidity averaged between 650 and 1000 hPa for the sensitivity run relative to the control run, shown for CAM5 (upper panels) and ECHAM5 (lower panels). The prescribed SST cooling anomaly in the sensitivity run relative to the control run (contour interval: -0.2 °C) is shown by blue dashed contours. Regions where the differences between the two runs are significant at the 95% confidence level are indicated by dots, based on a two-sample t test.

We emphasize that this idealized experiment is not intended to reproduce the detailed observed evolution over the past 41 years, given differences between the observations and our AMIP setup, as well as the fact that the model response is evaluated from 30-year mean differences whereas the observed change is expressed as a long-term trend (as a single realization). Rather, the two-model experiment is designed as a conceptual test to demonstrate the plausibility of the proposed mechanism. We have clarified this point ("in a qualitative sense") in the discussion of the AMIP experiment.

3. The authors should add the availability of the generated datasets used in this work (e.g., the AMIP experiments) in the Data Availability.

The data have been deposited on Zenodo (https://zenodo.org/records/18827534). The NetCDF files include 30 years of annual mean fields (Z200, Z850, and specific humidity). We have added this information in the Data Availability.

Reviewer #3 (Remarks to the Author):

The authors analyze the characteristics and potential mechanisms behind the observed "Moistening Hole" (MS) that has characterized large portions of the western US over the period 1980-2020. Their central thesis is that long-term cooling over the tropical eastern Pacific drives an atmospheric teleconnection pattern consisting of ridging over the North Pacific and subsequent descending motion on the eastern flank of this ridge over the western US. They conduct dedicated numerical experiments to explore this, as well as diagnostics with a large ensemble of global climate model simulations and multiple reanalysis and observational datasets.

Overall this manuscript will be a useful contribution to the literature. I have some detailed comments below. However, I am not sure that this is an ideal candidate for Nature Communications for two reasons. First, I am not convinced that the results "... represent important advances of significance to specialists within each field." The results are interesting ... but I am not sure I would call them "important advances". The idea that cold conditions in the eastern tropical Pacific drive aridity over the western US has been thoroughly explored and documented in the literature, so that aspect may not be an important advance. Secondly, when a manuscript includes 29 figures in the Extended Data section, I question if it is really suitable for a short format journal. There are many times in the manuscript when the reader is referred to the Extended Data section to support important ideas and concepts. For example, the results of the prescribed SST experiments - an important component of the argument - are briefly discussed in the main body (lines 264-272) but the map of the results is relegated to Extended Data Figure 14. Even a short paper should be able to largely stand on what is in the main paper, and I am not sure that is the case here. I feel this work would be much better suited for a longer format journal.

Thanks for sharing your thoughts on our paper. In terms of physical analysis and understanding, we believe our work represents a meaningful step forward by revealing the detailed processes underlying the moistening cycle over the western US. To our knowledge, this is the first study to clearly demonstrate that the pathway linking tropical SST anomalies > large-scale circulation > vertical motion > precipitation > evaporation and atmospheric drying is central to transmitting remote tropical forcing to local drying. Second, we quantify the relative roles of different moisture sources in shaping drying over the western US. Third, we propose a new phenomenon, coined the “moistening hole”, to highlight the unusual decline in atmospheric moisture over the western US amid the broader, global moistening trend over the past ~40 years.

We also agree that the manuscript had become oversized, particularly because of the volume of Supplementary Material. Much of this content was added in response to requests from reviewers in earlier rounds. We also felt it was important to be transparent by presenting supporting figures rather than repeatedly referring to “figures not shown.” Together, these factors contributed to the length of the Supplementary Information.

To achieve a better balance, as you suggested, we have moved three key figures from the Supplementary Information to the main text: the new AMIP experiment, the schematic diagram, and the sea-ice index derived from CESM2-LE, taking advantage of the journal’s allowance of up to 10 display items in the main text. The remaining supplementary figures primarily provide

additional sensitivity tests and supporting evidence. In its current form, we believe the logical flow is much stronger, and readers can get the main messages of the work even without the Supplementary Information.

The science in the manuscript is sound but arguably more incremental than a major advance. Others may feel differently, and I would certainly respect those assessments, but it is my assessment that this manuscript would be an excellent contribution to a longer format journal, where material in the Extended Data section could be folded more naturally into the main body of the manuscript.

We appreciate your comment about streamlining the supplementary material and integrating key content into the main text. We have moved three key figures from the Supplementary Information to the main text, and we hope these changes improve the flow and readability of the manuscript. Scientifically, as emphasized above, the three main contributions of this study, diagnostic analysis of the dynamical pathway, the moisture-tagging attribution, and the finding of the moistening hole, are all proposed here for the first time, and we believe they provide substantial new insight.

On another general note, I find the figure quality to be below average. One specific approach that could help (for example in Fig. 1) would be to only stipple or obscure areas that are not statistically significant, thus allowing a clearer visual inspection of areas that are significant and thus clear of any stippling. I also found the color of the coastlines to not stand out. Also, the text in the figure labels appears blurry. Overall, the figures are not as clear and crisp as I would hope for.

The issue is likely that the online submission portal converts the Word file and compresses the figures. This time, we convert the document to PDF locally and submitted the PDF directly. We hope this version is much clearer than the previous one.

Specific questions/comments

1. For the water-tagging ... is it the case that the water is tagged from the location it first evaporates into the atmosphere until the point where it precipitates to the surface? Is the "memory" of the tagging lost after that point? For example, if there was evaporation over the Pacific which fell out as precipitation over California, but was then evaporated again and fell out as precipitation over Colorado ... would the "source" for that precipitation over Colorado be California (continental) rather than the Pacific (oceanic)? With multiple re-evaporation cycles possible for water parcels how useful is that for tracking longer range sources of moisture? Also, what assumptions are made about moisture parcel transport during any nonlinear atmospheric processes, such as convection?

Yes. There is no “memory effect” in the current water-tagging approach. In your case, the tagged water over Colorado indicates only that its immediate moisture source is California, not the Pacific. As we know, all precipitation over land ultimately originates from the ocean, regardless of how many times water has been recycled. In our tagging framework, however, we track only the immediate, direct contribution that supplies moisture for the most recent precipitation event. Our focus is therefore on how this immediate hydroclimate pathway changes over time, and we find that the local contribution plays an important role in the formation of the MH. Tracking multiple rounds of recycling is not feasible in iCESM and, to the best of our knowledge, in any other available modeling framework: once water returns to the ocean (or is stored on land), iCESM does not have code infrastructure to track its subsequent pathways through reservoirs such as soil moisture, vegetation, or groundwater or determine when (or where) it will re-evaporate into the atmosphere and contribute to precipitation again.

We do not make any additional assumptions in our water tagging. The implementation follows the isotope-enabled CAM framework in iCESM (CAM5), in which water tagging is done by reusing the existing isotope moisture-tracer infrastructure. Each tag is treated like an isotope tracer, but without isotope fractionation. The model simulates all moist processes (convection, cloud microphysics, condensation, precipitation formation, and re-evaporation) identically to the standard CAM for each tagged component in water vapor, cloud liquid/ice, and precipitation. This moisture-tagging framework has been extensively validated and applied (e.g., Singh et al., 2016). In validation tests, the sum of precipitation from the individual tags nearly perfectly reproduces the bulk precipitation simulated by CAM (with error < 2%).

Singh, H. A., C. M. Bitz, J. Nusbaumer, and D. C. Noone (2016), A mathematical framework for analysis of water tracers: Part 1: Development of theory and application to the preindustrial mean state, J. Adv. Model. Earth Syst., 8, 991–1013, doi:10.1002/2016MS000649.

2. Line 111 The units for the trend of specific humidity are listed here as "%/decade" whereas the figure caption (1b) lists "g/kg/decade". There should be consistency between the text and figure.

This is intended for comparison with the 1980–1990 period. This number reflects the rate of change over the later interval relative to the first ten years, so we present it in a relative sense.

3. Line 126 Should "anomalies" be "positive linear trends:?

“Anomalies” has been rephrased as “trends.”

4. Line 133 The "wave train emanating from the tropical Eastern Pacific" is not obvious to me from Extended Data Fig. 3. Perhaps clarify that with some additional text.

The wave-activity flux arrows have been enlarged to improve clarity. We also added text there noting that the wave-activity flux indicates a wave train emanating from the tropical eastern Pacific toward the western US.

5. Line 137 Do you mean no change in the trend, or no trend? Also, line 135 refers to "minimal SST warming trends" whereas line 139 refers to "SST cooling". Line 223 refers to "muted warming". Why the different terminology?

What we meant is “no SST trend” in the raw sense. When we refer to “no SST trend,” “minimal SST warming” (line 135), or “muted warming” (line 233), we mean that the raw SST change over the tropical eastern Pacific is small because it is embedded within a basin-wide tropical warming signal associated with global warming. If this tropical-wide warming signal is removed, a slight cooling emerges (line 139). We have rewritten “minimal SST warming trends” and “muted warming” as “little to no SST trend” and “lack of warming…”, respectively.

6. Line 178 Isn't it usually the case that enhanced downward motion is associated with reduced precipitation?

The statement “downward motion leads to less precipitation” is a good first-order expectation. Ascent increases the likelihood of saturation, but precipitation is also constrained by moisture availability and microphysical processes. We have added “to first order” to that sentence.

7. Line 234 Is it accurate to say "the same climate forcing" here since different models have different radiative responses to identically imposed changes in atmospheric composition?

Agree. “the same climate forcing” has been rephrased to “the climate forcing specified by the CMIP6 experimental protocol.”

8. Line 256 Since this discussion refers, I believe, to comparisons within members of the CESM2 ensemble, I don't think it is accurate to say at this point in the paper "driven by tropical Pacific SST cooling in the model" as that causality has not yet been shown ... that statement needs the fixed SST experiments discussed later.

Agree. “Driven by” has been changed to “ linked to “

9. Line 306 ... typo with extraneous left bracket.

Corrected

10. Line 342 I was a little confused by the "two-way capacitor" analogy.

We have removed those two words, since “capability” already captures the sense of resilience and reservoir function, which is the main point we want to convey about the role of soil in connecting precipitation and evaporation.

Reviewer #3 (Remarks to the Author):

I thank the authors for the revision. While the concerns I have previously expressed remain in place, I respect the judgement of the other reviewers and Editors and therefore am supportive of publication.

Thank you for your support of this publication. The remaining concerns seem to center on the tagging method, which is relatively new and may therefore feel unfamiliar. Even so, I believe it is a powerful tool. Although it has limitations, it provides important insight into this topic. Before our analysis, no study had clearly shown or quantified that the drying is driven by local processes. This highlights the value of the approach and the new understanding it provides.

A few specific minor comments ...

1. Line 167 The text discusses the soil moisture trend in Fig. 3d, but the text along the top of the plot says evaporation. I suspect that should be "soil moisture".

Agreed. ‘Soil moisture’ has been added to the title of Fig. 3d.

2.Line 288 The text implies that we should be able to see from Fig 8a that zone 41 contributes 20% of the total atmospheric moisture over the Western US, but the figure shows the contribution to the trend. I believe this is only seen from Extended Data Fig. 13a.

Agreed. Fig. 8 is no longer referenced there.

3. I still o not find the water tagging experiments to be especially illuminating for the reasons I previously mentioned (the potential for a water parcel that is transported from a different region can be precipitated out and re-evaporated one or more times, thereby complicating the interpretation of the results).

We have added a sentence in the Methods section to acknowledge this limitation of the method.

Lines 687-690: “It should also be noted that, because tracking multiple rounds of recycling is not feasible in iCESM1.2, this tagging approach tracks only the immediate, direct moisture contribution to the most recent precipitation event, rather than moisture that has undergone multiple cycles of precipitation and re-evaporation.”

However, we would like to emphasize that this is not a major limitation. For example, if a moisture parcel evaporates from region A, precipitates over land region B, and then re-evaporates from B before finally precipitating over region C, the current framework can identify only the contribution from B to C. If one is interested in the full pathway from A to C, as the reviewer suggests in the context of multiple rounds of precipitation and re-evaporation, this information can still be inferred from our approach with additional analysis. By shifting the focus to region B and examining how rainfall changes there and what drives those changes, one can further assess A’s contribution to precipitation over B. We used this approach in our published study of multiple-round moisture transport to the Arctic (https://www.nature.com/articles/s43247-025-03000-x), which showed that recent Arctic moistening is associated with enhanced land evaporation from Eurasia, where the

increased evaporation is driven by more local precipitation and more remote moisture sources from the tropical Atlantic.
